# Photocatalytic Lignin Depolymerization and Cross‐Coupling With Alcohols to Produce Unsymmetric Aromatic Diols

**DOI:** 10.1002/anie.2045478

**Published:** 2026-06-09

**Authors:** Hongji Li, Jialing Ma, Xiaoling Wan, Yumei Liu, Chaofeng Zhang, Xiaoqin Si, Xiaojun Shen, Rajenahally V. Jagadeesh

**Affiliations:** ^1^ College of Chemistry Zhengzhou University Zhengzhou China; ^2^ Leibniz‐Institut Für Katalyse e.V. Rostock Germany; ^3^ College of Light Industry and Food Engineering Nanjing Forestry University Nanjing China; ^4^ School of Chemical Engineering Zhengzhou University Zhengzhou China; ^5^ Nanotechnology Centre Centre for Energy and Environmental Technologies VS ˇB‐Technical University of Ostrava Ostrava‐Poruba Czech Republic

**Keywords:** aromatic diols, biomass valorization, C‐C cross‐coupling, photocatalysis, tandem lignin depolymerization and functionalization

## Abstract

Conversion of lignin into chemical products typically involves two separate steps, such as depolymerization and subsequent upgrading of monomers, which often require different catalytic systems or varying reaction conditions. Notably, the direct one‐pot conversion of lignin into valuable compounds is crucial for advancing biomass utilization, aiming to streamline production by combining depolymerization and functionalization steps to maximize process‐, atom‐, and cost‐efficiency. Here we report the photocatalytic approach for synchronized depolymerization and C–C coupling processes for the direct conversion of lignin and its derivatives into aromatic diols using CdZnS (Cd_0.3_Zn_0.7_S) catalyst. By coupling hole‐driven radical generation with electron‐driven regeneration of overoxidized benzylic intermediates, this strategy overcomes the intrinsic preference for homocoupling and enables selective cross‐coupling between benzylic and aliphatic alcohol derivatives. This CdZnS‐based photocatalytic process enables simultaneous hydrogen transfer, hydrogenolysis and cross‐coupling of various lignin model compounds with alcohols to obtain unsymmetrical aromatic diols. In the case of real lignin, this depolymerization‐cross‐coupling protocol gives corresponding aromatic diols up to 10 wt% monomer yield. The resulting diol products are directly applicable to the synthesis of thermoplastic polyurethanes (TPUs) as well as fused polycyclic frameworks, functional materials, and bioactive molecules.

## Graphical abstract

A photocatalytic approach for efficient one‐pot direct lignin depolymerization and subsequent cross‐coupling with alcohols for the production of unsymmetrical aromatic diols has been demonstrated.

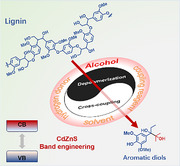

## 1 Introduction

**FIGURE 1:**
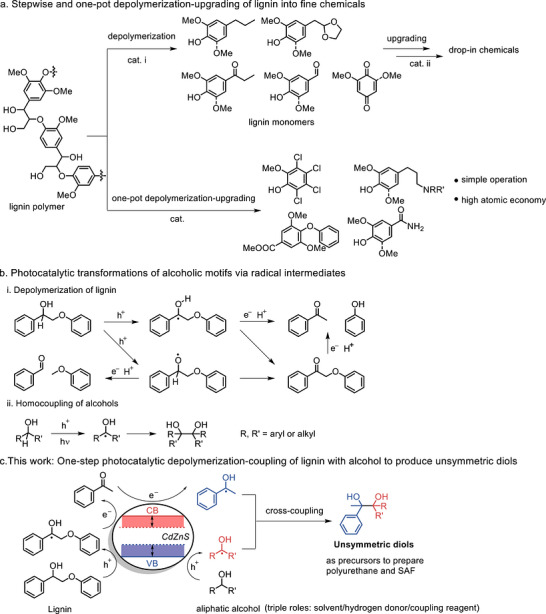
Photocatalytic transformation of lignin and alcohol via C–H bond activation, and our findings in photocatalytic cross‐coupling of alcohols and depolymerization‐coupling of lignin to deliver aromatic diol products.

The valorization of lignin, a naturally occurring biopolymer, offers a sustainable and renewable pathway for the production of valuable aromatic functional compounds as well as cycloaliphatic compounds‐based biofuels [1, 2]. Catalytic cleavage of C‐C and C–O bonds enables the lignin depolymerization into aromatic monomers [3, 4, 5, 6, 7], yet these products often exhibit limited added value and therefore require further upgrading to access essential chemical compounds with broader application scenarios (Figure 1a) [8, 9, 10]. Owing to the heterogeneous polymeric nature of native lignin and the substantial structural diversity ‐lignin depolymerization and upgrading of its derived monomers/compounds are commonly performed in two separate reaction steps using different catalytic systems. The integration of the individual steps in lignin valorization is often complicated by the need to isolate and purify intermediate monomers, leading to expensive processes, high energy consumption, and low atom economy [11]. In recent years, one‐pot lignin depolymerization‐upgrading strategies have emerged as an attractive alternative protocols, enabling the direct synthesis of functional compounds including aryl chlorides [12], diaryl ethers [13], aryl propylamines [14], aromatic amides [15], azaheterocyclic compounds [16], and other high‐value products (Figure 1a). These tandem processes inspired for further possibility for lignin valorization‐upgrading to produce other valuable compounds. In this context, we focused on the direct lignin conversion and upgrading to produce diols via synchronized depolymerization and C–C cross‐coupling reactions.

Diols represent an important class of platform molecules that are widely used in the manufacture of fine and bulk chemicals, including polymers, pharmaceuticals, cosmetics, and others [17, 18]. Due to their utility and diverse applications, the sustainable and cost‐effective production of diols from abundant and inexpensive starting materials is of central importance. In particular their production from renewable resources, such as biomass is gaining increasing importance. Although lignin inherently contains diol groups, these structures are inevitably destroyed during depolymerization. Therefore, the direct conversion of lignin into diol products would open new opportunities for practical lignin valorization to high‐value platform chemicals. A straightforward route to the production of diols is the C–C coupling of alcohols by radical reaction in the presence of a suitable photocatalyst, which has proven to be a promising and sustainable method [19]. In lignin chemistry, single electron oxidation of alcoholic motifs in β‐O‐4 linkages can generate carbon‐ or oxygen‐centered radicals, which induce β‐scission (C–O or C–C bonds cleavage) or form ketone intermediates that readily undergo C–O cleavage (Figure 1b) [20, 21, 22, 23, 24, 25, 26, 27, 28]. On the other hand, photocatalytic dehydrogenative coupling of alcohols proceeds via the selective oxidation of C_α–_H bonds in alcohols by photogenerated holes to form carbon‐centered radicals for subsequent C–C bond coupling [29, 30, 31, 32, 33, 34, 35, 36, 37, 38, 39, 40, 41, 42]. Nevertheless, these coupling reactions mainly give homocoupling products, affording symmetric diols with limited structural diversity. In contrast, cross‐coupling of two alcohols to provide unsymmetric diols with more tunable structures and broader applications is of potential importance [43].

Considering the widespread applications of aliphatic alcohols in the depolymerization and upgrading of lignin (e.g., as solvents, hydrogen donors, alkylating agents, and hydroxyl protecting reagents) and their role as important building blocks for diol synthesis, we hereby report a sustainable photocatalytic methodology for lignin depolymerization in aliphatic alcohols with subsequent cross‐coupling to prepare unsymmetrical diol monomers. In this protocol, the aliphatic alcohols serve a dual role as a hydrogen donors for lignin depolymerization and as a building blocks for diols formation. Notably, this tandem process enables synchronized depolymerization and upgrading in one‐pot using a single catalyst system, resulting in improved step‐, cost‐, and atom‐ economy. However, a key challenge in such a tandem reaction is to achieve a selective cross‐coupling between benzylic fragments derived from lignin and aliphatic alcohols. Benzyl alcohol moieties in lignin are more readily undergo C_α–_H activation than aliphatic alcohols, leading to preferential generation of benzyl alcohol radicals. In contrast, the stronger oxidation process required to generate aliphatic alcohol radicals would simultaneously drive benzylic substrates toward overoxidation to carbonyl intermediates. Therefore, it is difficult to sustain both radicals simultaneously through oxidation alone, and the system intrinsically favors homocoupling over cross‐coupling. To address this challenge, we propose a band‐engineered photo‐redox radical co‐matching strategy (Figure 1c). In this design, photogenerated holes activate both benzylic and aliphatic alcohols to generate two distinct carbon radicals, while photogenerated electrons reduce the over‐oxidized aromatic ketone intermediates back to the benzyl alcohol radicals. Such redox cooperation enables the coexistence of the two radicals and thereby promotes radical cross‐coupling between benzylic and aliphatic alcohols to afford unsymmetric diol products.

Based on this concept, we designed and applied CdZnS photocatalysts with tunable band structures to regulate the generation and fate of radical intermediates for the cross‐coupling of lignin‐derived aromatic alcohols with aliphatic alcohols to produce unsymmetrical diols. This catalytic system enables selective switching among benzylic alcohol oxidative dehydrogenation, homocoupling, and cross‐coupling, and further realizes tandem C–O bond cleavage and C–C cross‐coupling of lignin β‐O‐4 model compounds. More importantly, the photocatalytic method is successfully extended to native lignin depolymerization and in situ cross‐coupling with aliphatic alcohols to afford aromatic diol monomers. The resulting diol products not only serve as promising monomers for polyurethane synthesis, but can also be further upgraded to fuel‐range cycloalkanes and other functional compounds.

## 2 Results and Discussion

### 2.1 Photocatalyst Design

**FIGURE 2:**
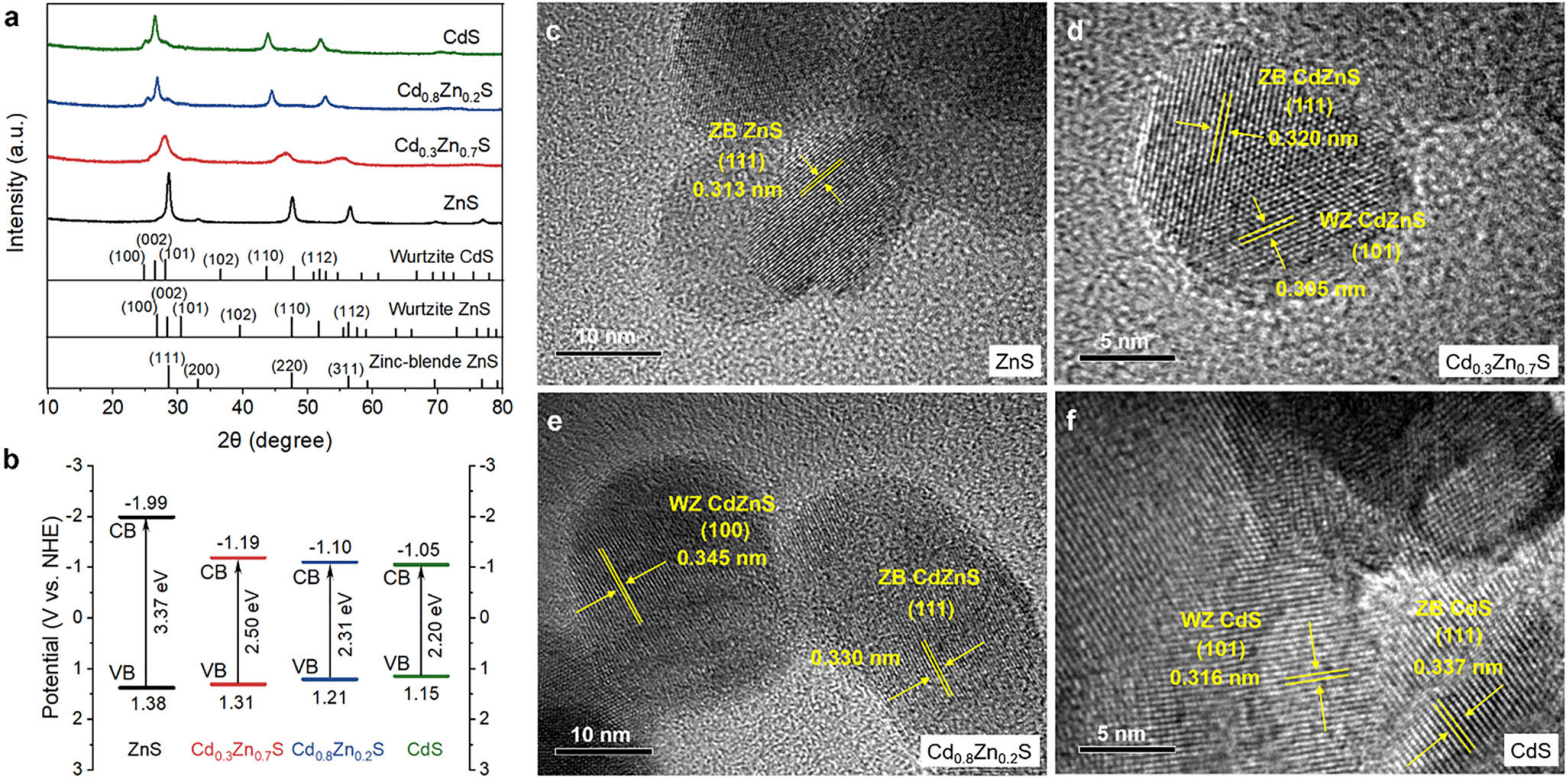
The characterizations of prepared Cd_x_Zn_1‐x_S photocatalysts. (a) XRD patterns. (b) Band structures. HRTEM images of ZnS (c), Cd_0.3_Zn_0.7_S (d), Cd_0.8_Zn_0.2_S (e), and CdS (f).

ZnS and CdS semiconductors are widely known for their potential to form mixed crystals that exhibit a large difference in band structure [44]. Consequently, the band positions of CdZnS materials can be precisely modulated over a wide bandgap range by varying the molar ratio of Cd^2+^ to Zn^2+^ [45, 46, 47, 48, 49]. In this work, four sulfide semiconductors with different Cd/Zn ratios (ZnS, Cd_0.3_Zn_0.7_S, Cd_0.8_Zn_0.2_S, and CdS) were synthesized via a hydrothermal method to screen the optimal catalyst for the cross‐coupling of 1‐phenyl‐1‐propanol (PPA) as an example of benzylic alcohol and isopropanol (IPA), an aliphatic alcohol. The structure and physicochemical properties of the prepared four sulfide semiconductors were systematically characterized (Figure 2). XRD patterns revealed that ZnS existed predominantly in the zinc blende (ZB) phase (Figure 2a). After the incorporation of Cd, a new crystalline phase was formed alongside the zinc blende phase, namely the wurtzite phase (WZ). Moreover, the diffraction peaks shifted to lower angles with increasing Cd content, indicating an increase in the lattice parameters due to the larger ionic radius of Cd^2+^ compared to Zn^2+^. SEM (Figure S1) and TEM images (Figure S2) showed that all samples consisted of nanoparticles with sizes in the range of 10 to 30 nm. The exposed crystal facets of these nanoparticles were identified by HRTEM (Figure 2c–f). In ZnS, the ZB(111) surface was predominantly present, and the interplanar distance of this surface gradually increased from 0.313 to 0.337 nm with Cd doping. Meanwhile, the appearance of the WZ(101) and WZ(100) facets further verifies the formation of a heterophase junction in CdZnS, which is consistent with the XRD results. Energy dispersive X‐ray spectrometry (EDS) mapping of Cd_0.3_Zn_0.7_S and Cd_0.8_Zn_0.2_S showed that Cd, Zn, and S elements were uniformly distributed (Figure S3), demonstrating the formation of CdZnS solid solutions. UV–vis diffuse reflectance spectra demonstrated that, the absorption edge of CdZnS continuously redshifted from 368 to 564 nm with increasing Cd content (Figure S5), leading to a gradual narrowing of the bandgap. The band structure of each catalyst was further determined based on the valence band positions measured by VB XPS (Figure S6). As the Cd/Zn ratio increased, both the valence band and conduction band potentials of CdZnS gradually shifted toward 0 V (Figure 2b), confirming the continuously tunable redox capability by adjusting the Cd/Zn ratio. The continuously tunable band structure of CdZnS is therefore expected not only to tune photocatalytic activity, but also to regulate the generation and fate of alcohol‐derived radicals, thereby determining whether the reaction proceeds through oxidative dehydrogenation, homocoupling, or cross‐coupling.(Figure 1)

### 2.2 Photocatalytic Radical Cross‐Coupling Between Benzylic Alcohols and Aliphatic Alcohols

**FIGURE 3:**
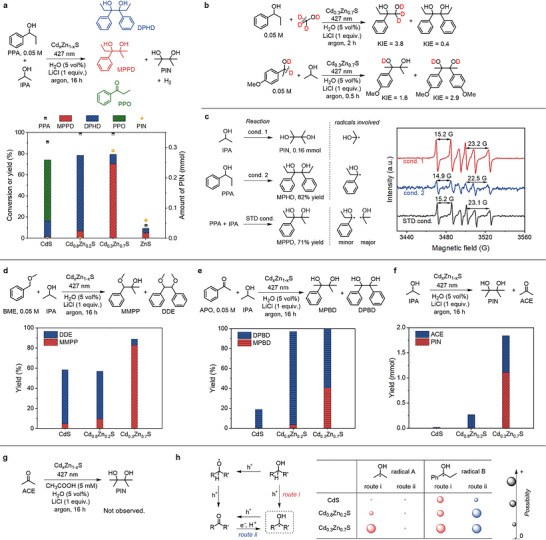
Photocatalytic dehydrogenative cross‐coupling between 3‐phenyl‐1‐propanol (PPA) and isopropanol (IPA). (a) The photocatalytic performance of different Cd_x_Zn_1‐x_S samples. (b) KIE studies. (c) Radical capturing experiments using DMPO as the trapping agent. Condition 1: Cd_0.3_Zn_0.7_S (10 mg), IPA (400 µL), H_2_O (40 µL), 427 nm LEDs, argon, 16 h. Condition 2: Cd_0.3_Zn_0.7_S (10 mg), PPA (0.3 mmol), *t*BuOH (400 µL), IPA (40 µL), H_2_O (40 µL), LiCl (2 mg), 427 nm LEDs, argon, 16 h. Standard condition: Cd_0.3_Zn_0.7_S (10 mg), PPA (0.1 mmol), IPA (2 mL), H_2_O (100 µL), LiCl (0.1 mmol), 427 nm LEDs, argon, 16 h. The amount of DMPO in all conditions was 100 µL. (d) Photocatalytic coupling between benzyl methyl ether (BME) and IPA. (e) Photocatalytic coupling between acetophenone (APO) and IPA. (f) Control experiments using IPA as the sole substrate. (g) Control experiments using acetone (ACE) as the sole substrate. (h) The possibility of different routes to generate the alcoholic radicals.

Since lignin contains abundant benzyl alcohol units, we first investigated the feasibility of alcohol cross‐coupling using small‐molecular 3‐phenyl‐1‐propanol (PPA) as a benzyl alcohol model substrate, with isopropanol (IPA) serving as the coupling partner, reactant and solvent, and LiCl and water as additives (Figure 3). The catalytic activity of Cd_x_Zn_1‐x_S series catalysts (*x* = 0, 0.1, 0.2, 0.3, 0.4, 0.5, 0.6, 0.7, 0.8, 1) was evaluated under 427 nm light irradiation (Figures 3a and S7). ZnS exhibited almost no activity under visible light irradiation. In contrast, CdS, Cd_0.8_Zn_0.2_S, and Cd_0.3_Zn_0.7_S, all enabled complete conversion of 3‐phenyl‐1‐propanol yet showed drastically distinct product selectivity. CdS mainly catalyzed the oxidative dehydrogenation of 3‐phenyl‐1‐propanol to propiophenone (PPO), accompanied by a small amount of the dehydrogenative homocoupling product DPHD, whereas the isopropanol homocoupling product PIN was not detected. This indicates that CdS with relatively weak redox ability is sufficient to oxidize benzylic alcohol, but exhibits poor selectivity toward benzylic radicals. Upon Zn incorporation, the reaction pathway shifted from oxidative dehydrogenation to radical coupling. At a Zn content of 20%, the homocoupling product was observed as the main product, with virtually no symmetrical pinacol being formed. This suggests that isopropanol is not activated to generate aliphatic alcohol radicals. With increasing the Zn content (>20%), the formation of the corresponding symmetrical pinacol gradually increases, which demonstrates the generation of carbon radicals from isopropanol. The optimal yield of the cross‐coupling product was achieved at a Zn content of 70%. However, upon further increasing the Zn content (>70%), the yield of cross‐coupling product decreased; this may be attributed to the fact that an excessively high Zn content widens the band gap of the catalyst and weakens its light absorption capacity. As a result, Cd_0.8_Zn_0.2_S mainly afforded the benzylic homocoupling product DPHD, together with a minor amount of cross‐coupling product, indicating efficient generation of benzylic alcohol radical equivalents, but only limited formation of isopropanol radicals. Further increasing the Zn content (Cd_0.3_Zn_0.7_S) markedly boosted the yield of PIN, indicating abundant isopropanol radicals were produced in the reaction system. Meanwhile, the cross‐coupling product MPPD became the dominant product with a yield of 71%, far exceeding that of the homocoupling product DPHD (9%). This result shows that selective cross‐coupling is achieved when the formation of aliphatic radicals becomes sufficiently competitive with that of benzylic alcohol radicals. Therefore, tuning the Cd/Zn ratio in the CdZnS system does not merely change catalytic activity, but regulates the relative generation and fate of benzyl alcohol radicals and isopropanol radicals, thereby switching the reaction pathway among oxidative dehydrogenation, homocoupling, and cross‐coupling. Among the prepared catalytic systems, Cd_0.3_Zn_0.7_S displayed the optimal performance for cross‐coupling to produce the desired diol product. During the photocatalytic conversion over Cd_0.3_Zn_0.7_S, hydrogen was mainly generated from isopropanol transformation, and its amount was nearly consistent with the total yield of acetone and PIN (Figure S8). Under optimal conditions, using Cd_0.3_Zn_0.7_S, about 2.3 mmol of hydrogen was produced in the model reaction with a maximum yield of MPPD.

To further elucidate the origin of the superior photocatalytic activity of Cd_0.3_Zn_0.7_S, the carrier separation efficiency of catalysts was characterized. With decreasing Cd/Zn ratio in CdZnS, the photocurrent density of the catalysts gradually decreased (Figure S9), while the photoluminescence intensity under light irradiation steadily increased (Figure S10), confirming a decrease in charge carrier separation efficiency. Notably, Cd_0.3_Zn_0.7_S exhibited inferior carrier separation efficiency, but the best cross‐coupling activity, demonstrating that carrier separation efficiency is not the dominant factor governing this reaction. Instead, product selectivity is primarily dictated by band‐position‐dependent control over radical generation and fate. Among the visible‐light‐responsive catalysts, Cd_0.3_Zn_0.7_S combines a more positive valence band and a more negative conduction band (Figure 2b), which is favorable for the activation of both aliphatic alcohol and benzyl alcohol as well as their radical regeneration, thereby leading to the highest cross‐coupling selectivity.

After identifying the optimal catalyst, the roles of LiCl and water as additives were systematically investigated (Figure S11). In the absence of any additives, Cd_0.3_Zn_0.7_S favored the formation of the homocoupling product with a high yield of 86%, whereas the cross‐coupling product was obtained in only 12% yield, accompanied by merely 4 µmol of PIN. This result indicates that, the generation of isopropanol radicals was severely suppressed under additive‐free conditions. The introduction of LiCl significantly promoted the formation of the cross‐coupling product, increasing the yield of the target product to 46%, which suggests that LiCl facilitates the activation of isopropanol, possibly by promoting its interaction with the catalyst surface. Other lithium salts and alkali metal chlorides showed inferior catalytic activity compared with LiCl, suggesting that both lithium ions and chloride ions synergistically contributed to enhanced performance. As reported in previous literature, water can stabilize alcohol radicals via hydrogen bonding interactions [32], thereby facilitating radical coupling and inhibiting oxidative dehydrogenation. When water alone was used as the additive, both the yield of PIN (220 µmol) and MPPD (55%) increased dramatically, demonstrating that water mainly stabilizes isopropanol radicals and promotes the formation of aromatic cross‐coupling products. The combination of water and LiCl exerted a synergistic effect on the activation of isopropanol, resulting in a PIN yield of 290 µmol and an increased target product yield of 71%. Thus, the combination of LiCl and H_2_O promotes the generation and stabilization of isopropanol radicals, thereby favors selective cross‐coupling.

To clarify the influence of substrate C–H bond activation on the reaction, kinetic isotope effect (KIE) studies were conducted (Figure 3b). When using deuterated aliphatic alcohol, the KIE value for the formation of cross‐coupling products reached 3.8, which is characteristic of a primary KIE. This result confirms that, the activation of C_α–_H bonds of aliphatic alcohols is the rate‐determining step in this cross‐coupling reaction. In contrast, when deuterated benzylic alcohol was used, the KIE value for homocoupling product formation was 2.9, implying that C–H bond activation in benzylic alcohol directly governs the generation of benzylic alcohol radicals. Notably, the KIE value for cross‐coupling product formation was only 1.6, revealing that the C–H bonds in benzylic alcohol are more readily activated than those in aliphatic alcohols. These results indicate that the key kinetic bottleneck for cross‐coupling is not the formation of benzylic alcohol radical, but the more demanding generation of aliphatic alcohol‐derived radicals.

Radical trapping experiments using DMPO were performed to probe the intermediates involved in the coupling reactions (Figure 3c). In the homocoupling system, both aliphatic alcohol radicals and benzylic alcohol radicals were detected, confirming that the coupling reaction proceeds via a radical pathway. In the cross‐coupling system, however, the dominant trapped radical species was the aliphatic alcohol radical (Figure 3c), indicating a higher concentration of isopropanol radicals that were formed compared to benzyl alcohol radicals in the reaction system. This result suggests that selective cross‐coupling is achieved not by suppressing benzylic alcohol radical formation, but by increasing the relative concentration of aliphatic radicals so that cross‐coupling outcompetes benzylic homocoupling [50].

After confirmation of the radical pathway, the formation mechanism of the corresponding alcohol radicals was further investigated. When Na_2_S_2_O_3_ was added as an electron sacrificial agent in the reaction of 3‐phenyl‐1‐propanol and isopropanol catalyzed by Cd_0.3_Zn_0.7_S, no substrate conversion was observed (Figure S12). In contrast, upon the addition of Na_2_S_2_O_8_ as a hole sacrificial agent, the benzylic alcohol substrate mainly underwent oxidative dehydrogenation (Figure S12). These results demonstrate that both photogenerated holes and photogenerated electrons play indispensable roles in the alcohol cross‐coupling reaction. Meanwhile, the addition of acids or bases led to a remarkable decrease in the yield of the diol product (Figure S13), suggesting that the generation of these two radicals likely proceeds via a proton‐coupled electron transfer (PCET) mechanism.

Previous studies have reported that alcohol radicals can be generated either via direct C–H bond activation or from the reductive conversion of oxidized alcohol intermediates (i.e., carbonyl compounds) [31, 39, 40]. To clarify the radical generation pathways, a series of substrate control experiments were performed. The generation pathway of benzylic alcohol radicals was first explored. Benzyl ether was used as a substrate (Figure 3d), which can only form benzyl radicals via C–H bond activation. All three catalysts efficiently converted benzyl ether into coupling products, confirming their excellent capability to activate benzylic C–H bonds. However, CdS and Cd_0.8_Zn_0.2_S failed to generate aliphatic alcohol radicals, only yielding benzyl ether homocoupling products. On the other hand, Cd_0.3_Zn_0.7_S was able to simultaneously activate both benzyl ether and aliphatic alcohols, generating cross‐coupling products. When aromatic ketones were used as substrates, benzylic alcohol radicals could only be obtained via one‐electron reduction (Figure 3e). CdS showed poor activity toward aromatic ketone reduction, consistent with its weak reductive capacity; in contrast, both Cd_0.8_Zn_0.2_S and Cd_0.3_Zn_0.7_S could reduce the carbonyl group of aromatic ketones to afford diol coupling products. These results indicate that benzylic alcohol radical equivalents can be supplied through two pathways: Direct benzylic C–H activation and electron‐mediated reduction of over‐oxidized ketone intermediates.

Then the generation pathway of aliphatic alcohol radicals was explored. When acetone was employed as a model substrate (Figure 3g), no reductive coupling of acetone to PIN was observed over any catalyst upon the introduction of external protons, indicating that CdZnS catalysts lack the ability to reduce ketones to form alcohol radicals. Therefore, the isopropanol radicals involved in PIN formation during isopropanol conversion are exclusively generated via direct C_α_–H bond activation. Consistently, CdS showed no activity toward aliphatic alcohol conversion (Figure 3f), whereas Cd_0.8_Zn_0.2_S could oxidize isopropanol to acetone. These results demonstrate that direct generation of aliphatic radicals is a critical requirement for selective cross‐coupling. Notably, Cd_0.3_Zn_0.7_S exhibited high oxidation activity toward isopropanol and effectively stabilized aliphatic alcohol radicals, affording PIN as the main product (Figure 3f).

Based on the results above, the radical generation pathways of aliphatic alcohols and benzylic alcohols over CdZnS catalysts were summarized, which rationalized the distinct product selectivities in the cross‐coupling of aromatic and aliphatic alcohols. Cyclic voltammetry (CV) measurements also revealed that benzylic alcohol possesses a lower oxidation potential than isopropanol, while aromatic ketones exhibit a more positive reduction potential than acetone (Figure S14). Thus, the redox capability of CdZnS determines not only which radical species are generated, but also whether they arise from direct oxidation or from reductive regeneration pathways (Figure 3h). The Cd_0.3_Zn_0.7_S possesses sufficiently strong oxidation and reduction ability to generate aliphatic alcohol radicals through direct C–H activation while sustaining benzylic alcohol radical equivalents through both direct benzylic activation and aromatic ketone reduction, thereby enabling selective cross‐coupling. Cd_0.8_Zn_0.2_S possesses moderate redox capability with weak activity for direct C_α_–H bond activation in isopropanol, but can generate benzylic alcohol radicals via two pathways, thus affording benzylic alcohol homocoupling products as the main product and cross‐coupling products as byproducts. CdS has the weakest redox ability; it can only activate benzylic C–H bonds of benzylic alcohol, but shows poor reductive capacity for aromatic ketones, failing to stabilize benzylic alcohol radicals and thus yielding oxidative dehydrogenation products as the primary species. Taken together, these results support a dual radical‐supply network in which selective cross‐coupling becomes possible only when direct aliphatic radical generation and benzyl alcohol radical regeneration operate cooperatively. The quantum yield of the target product MPPD over the optimal catalytic system was calculated and is found to be in the range of 0.14%–0.21% under optimal conditions (see details in the Supporting Information).

**FIGURE 4:**
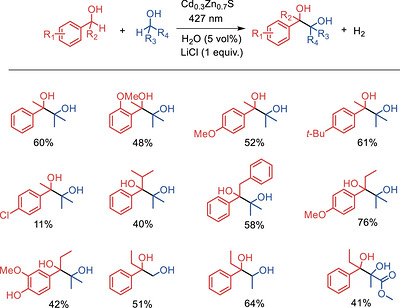
The substrate scope of the developed photocatalytic dehydrogenative cross‐coupling system using Cd_0.3_Zn_0.7_S. Reaction conditions: Benzylic alcohol (0.1 mmol), aliphatic alcohol (2 mL), Cd_0.3_Zn_0.7_S (10 mg), water (100 µL), LiCl (0.1 mmol), blue LEDs (427 nm, 40 W), argon, 16 h.

After having designed the optimal catalyst and reaction conditions, the cross‐coupling of different benzylic alcohols and aliphatic alcohols was performed (Figure 4). Various benzylic alcohols can undergo dehydrogenative cross‐coupling with isopropanol to afford unsymmetric diol products, in yields up to 76%. The increase of steric hindrance around the benzylic hydroxyl group led to a significant decrease in cross‐coupling product yield. In addition, methanol and ethanol can also undergo efficient coupling with benzylic alcohol, affording diol products in 51%–64% yields. Further, functionalized aliphatic alcohols with ester, amide, and halogen groups have been tested (Figure S15). Among these, methyl lactate was coupled with benzyl alcohol, yielding the corresponding symmetrical diol product in a yield of 41%. In contrast, the coupling of trichloro ethanol, trifluoroethanol, and glycolamide did not proceed to provide the desired unsymmetrical diols. Catalyst recycling tests show that the photocatalyst maintains 85% activity after three cycles, demonstrating good stability (Figure S16).

### 2.3 Photocatalytic Depolymerization and Cross‐Coupling of Lignin Models With Aliphatic Alcohols

**FIGURE 5:**
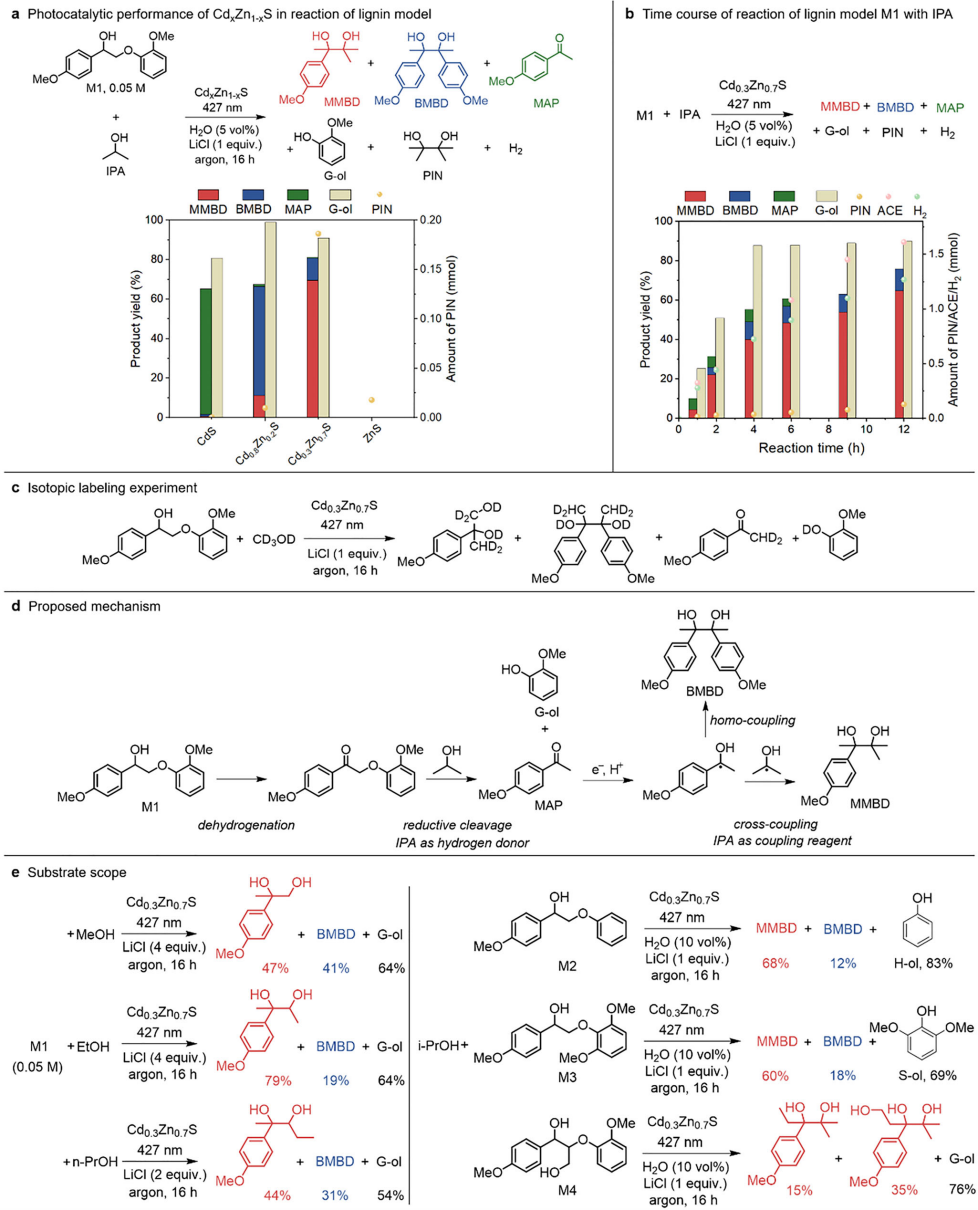
Photocatalytic cleavage and coupling of lignin models in aliphatic alcohols using Cd_0.3_Zn_0.7_S. (a) The photocatalytic performance of different Cd_x_Zn_1‐x_S samples. Conditions: Cd_x_Zn_1‐x_S (10 mg), PPA (0.1 mmol), IPA (2 mL). (b) Time course of reaction between lignin model and isopropanol using Cd_0.3_Zn_0.7_S as the photocatalyst. (c) Isotopic labelling experiments using deuterated alcohol. (d) A proposed mechanism for cleavage‐coupling of β‐O‐4 linkage. (e) The scope of alcohols and lignin models.

Based on the successful cross‐coupling between benzyl alcohol and aliphatic alcohols, the added value of lignin model compounds in aliphatic alcohols was further investigated (Figure 5). Notably, Cd_0.3_Zn_0.7_S simultaneously catalyzed the cleavage of β‐O‐4 linkages and the cross‐coupling of the resulting aromatic intermediates with aliphatic alcohols. Model compound **1** underwent cleavage to afford guaiacol in 91% yield, the unsymmetric diol in 70% yield, and the symmetric diol in 11% yield (Figure 5a).

The CdS and the Cd_0.8_Zn_0.2_S also enabled the efficient cleavage of β‐O‐4 linkages to generate phenols in high yields, whereas the other aromatic products gave ketones and symmetric diols, respectively, consistent with the product distribution observed in the corresponding alcohol cross‐coupling reactions. The ZnS remained inactive toward this transformation. These results indicate that, the tandem cleavage‐coupling of lignin model compounds follows the same band‐structure‐dependent radical chemistry as that identified in the benzylic/aliphatic alcohol cross‐coupling system. Control experiments (Figure S17) suggested that LiCl and water served as essential additives to generate the cross‐coupling product with high selectivity.

Time‐course monitoring of the reaction revealed that the formation rate of guaiacol was significantly faster than that of the diol products (Figure 5b), indicating that β‐O‐4 linkage cleavage precedes the coupling reaction rather than occurring after prior alcohol coupling. In addition, the amount of H_2_ produced during the reaction was lower than the total yield of acetone and PIN, indicating that part of the hydrogen equivalents generated from alcohol dehydrogenation was consumed in concurrent β‐O‐4 linkages hydrogenolysis, consistent with previous reports on sulfide‐semiconductor‐catalyzed lignin model cleavage [27]. When deuterated aliphatic alcohols were employed, deuterium incorporation was observed in both the coupling products and the aromatic ketone products (Figures 5c and S18), confirming that hydrogen species generated from aliphatic alcohol dehydrogenation participated in the hydrogenolysis of β‐O‐4 linkages. Accordingly, a mechanism for the transformation of β‐O‐4 linkages in isopropanol was proposed (Figure 5d): Isopropanol solvent, not only serves as the hydrogen source for the hydrogenolysis of β‐O‐4 linkages, but also acts as a C–C coupling reagent in the subsequent valorization of the cleaved products.

Besides isopropanol, the hydrogenolysis–coupling of model compound **M1** also proceeded in methanol, ethanol, and *n*‐propanol (Figure 5e), affording unsymmetric diols with varied carbon‐chain lengths in 44%–66% yields together with guaiacol in 54%–83% yields, accompanied by a minor amount of symmetric aromatic diols (19%–41% yields). Other simple model compounds **M2** and **M3** also underwent hydrogenolysis–coupling in isopropanol to produce unsymmetric diols along with phenol or 2,6‐dimethoxyphenol (Figure 5e). The more complex model compound **M4**, which better mimics the native β‐O‐4 linkages in lignin, was converted into phenol‐substituted hexanetriol and hexanediol products. The successful conversion of these model compounds suggests that the conversion of real lignin is also possible using the photocatalytic reaction strategy.

### 2.4 Photocatalytic Depolymerization and Cross‐Coupling of Lignin With Aliphatic Alcohols

After the cleavage and coupling of model compounds with aliphatic alcohols, we then focused on the real lignin valorization to produce diols. The balsa wood lignin was first extracted using 1,4‐dioxane to obtain unprotected lignin (UL). Acetaldehyde was added during the acid‐catalyzed extraction to protect the 1,3‐diol moiety in β‐O‐4 linkages, affording protected lignin (PL) according to literature procedures [51]. The protected lignin can undergo hydrolysis to produce normal lignin during reaction in the presence of water. Two‐dimensional heteronuclear single‐quantum coherence spectroscopy (2D HSQC) was employed to quantitatively analyze the ratio of aromatic units and the relative abundance of internal linkages (Figures S19 and S20). The S/G ratio of UL was 3.5, with a relative content of β‐O‐4 linkages of 46%. In contrast, the S/G ratio of PL remained almost unchanged (3.3), while the relative content of β‐O‐4 linkages with acetal‐protected structures increased to 58%. These structural differences make UL and PL suitable substrates to examine how lignin structure influences the tandem depolymerization‐coupling process.

**FIGURE 6:**
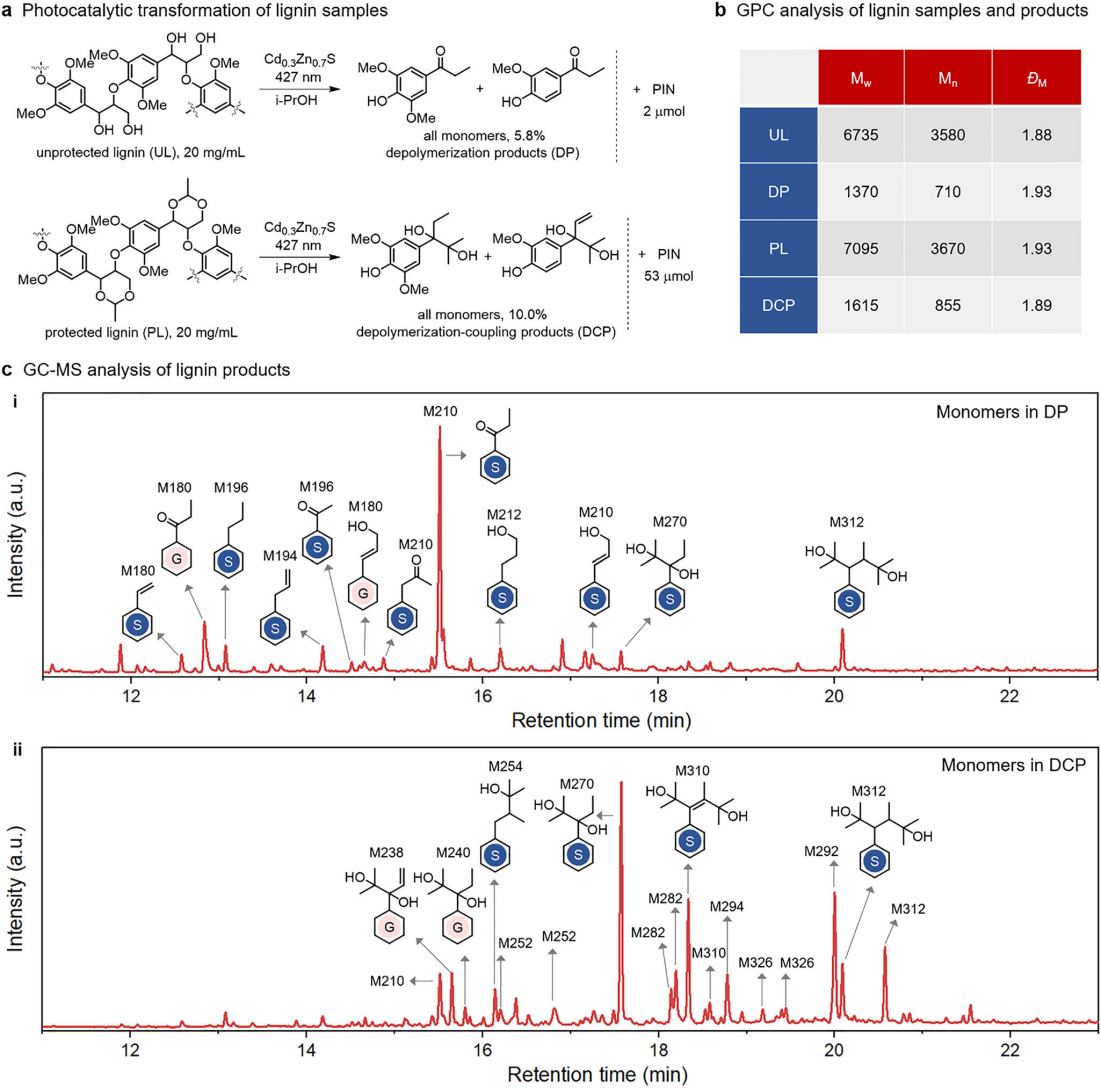
The photocatalytic depolymerization‐coupling of lignin in isopropanol using Cd_0.3_Zn_0.7_S catalytic system. (a) Monomer yields. (b) GPC analysis of lignin samples before and after transformation. (c) GC‐MS analysis of lignin monomers in DP and DCP.

Both lignin samples were subjected to the photocatalytic tandem depolymerization‐coupling transformation (Figure 6). The monomers obtained from UL depolymerization were mainly phenols with carbonyl or monohydroxyl group side chains (*m/z* = 180∼212), whereas only trace amounts of coupling products with isopropanol were detected. The total monomer yield reached 5.8%, and the molecular weight of the whole lignin decreased from 6735 to 1370 Da, indicating significant depolymerization. In contrast, the main monomers derived from PL depolymerization were coupling products between lignin aromatic monomers and isopropanol (*m/z* = 238∼326), featuring diol motifs on side chains, with an overall monomer yield of 10.0%. The molecular weight of all products after PL depolymerization was 1615 Da, which was also notably higher than that of the depolymerized products of UL, consistent with the incorporation of aliphatic alcohol fragments into the upgraded monomer products. These results show that PL not only undergoes effective depolymerization, but more importantly, enables the tandem depolymerization‐coupling sequence required for direct production of aromatic diols.

Notably, the reactivity of UL differs significantly from that of model compounds. This may be attributed to the dark red color of the unprotected lignin solution (Figure S21), which hinders the photocatalyst from absorbing visible light, whereas the model compound solution is colorless and imposes no interference on the catalyst's light absorption. After acetaldehyde protection, lignin appears pale yellow, which weakens the shielding effect on the catalyst's light absorption. Based on the reaction mechanism of model molecules, the activation of aliphatic alcohols is more challenging than the activation of benzyl alcohols and the cleavage of β‐O‐4 bonds, making them more susceptible to the photoresponsive performance of the photocatalyst. Indeed, only 2 µmol of PIN was detected during UL conversion (Figure 6a), suggesting that isopropanol radicals are barely generated under the applied reaction conditions. By contrast, 53 µmol of PIN was obtained during the PL transformation, demonstrating that successful lignin depolymerization‐coupling requires, not only benzylic alcohol activation and bond cleavage, but also sufficient photo response to sustain the aliphatic radical partner needed for cross‐coupling. In addition, hardwood, softwood, and herbaceous lignin were also used as raw materials to prepare aromatic diol products (Figure S22). Among these, birch and beech lignin exhibited favorable conversion behavior, yielding monomer yields of 6.4%–9.2%, while pine and corn cob lignin provided monomer yields of less than 3%, which was likely attributable to their low content of β‐O‐4 linkages. Further optimization of this reaction system is still needed to improve its applicability to diverse lignin species.

Compared with conventional aromatic monomers from direct lignin depolymerization, the unsymmetric aromatic diol monomers obtained in this work possess two distinctive features: An extended side‐chain carbon skeleton and dihydroxyl functional groups on the side chain. These features not only distinguish them from typical lignin‐derived monomers, but also make them attractive intermediates for downstream valorization. On this basis, the potential applications of lignin‐derived aromatic diols in the production of sustainable aviation fuels (SAF) and the synthesis of polyurethanes were separately investigated.

Lignin‐derived aromatic compounds serve as ideal feedstocks for producing cycloalkane and aromatic hydrocarbon components in SAF [52, 53]. However, monomers and dimers obtained from direct lignin depolymerization typically yield cycloalkanes below C_9_ or above C_16_ after hydrodeoxygenation, whereas the C_9–_C_16_ range required for aviation fuels often requires additional alkylation of aromatic monomers. In contrast, the present depolymerization‐cross‐coupling strategy directly furnishes side‐chain‐extended aromatic monomers through in situ coupling with aliphatic alcohols, making them more suitable precursors for producing fuel‐range C_10–_C_12_ cycloalkanes via hydrodeoxygenation. Preliminary experiments using NiMoO_x_ as the catalyst successfully afforded deoxygenated C_10–_C_12_ products at 220–250° C under 40 bar H_2_ (Figures S23–S25), accompanied by minor side products from dealkylation and alkyl migration. Although further optimization of this reaction or catalyst design is needed to improve selectivity, these results show that the lignin monomers obtained here are promising precursors for SAF.

**FIGURE 7:**
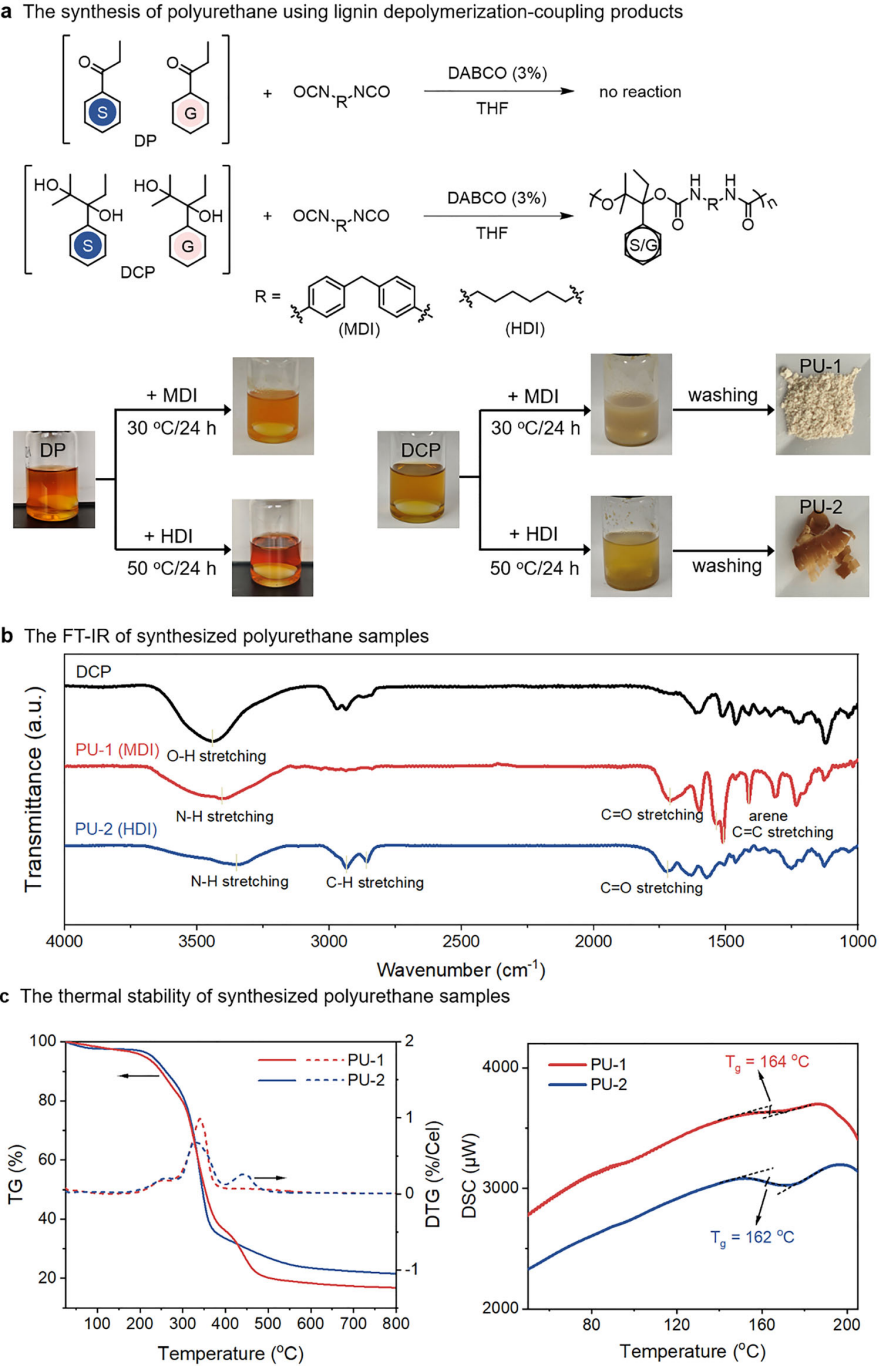
The preparation, structural characterization, and thermal stability of lignin‐based polyurethane samples. (a) The preparation process of polyurethane samples using DP and DCP. (b) FT‐IR spectra of PU‐1 and PU‐2. (c) The TG and DCS curves of PU‐1 and PU‐2.

Although numerous studies have been conducted on the synthesis of lignin‐based polyurethanes, lignin is usually used as a functional filler in most cases, requiring the additional introduction of polyols to react with isocyanates [54, 55]. By contrast, the products obtained from photocatalytic depolymerization‐cross‐coupling in this work are not merely lower‐molecular‐weight lignin fragments, but structurally upgraded, polyhydroxylated monomers that can serve directly as polyurethane precursors. The ^31^P NMR analysis (Figure S26) indicated that the relative content of aliphatic hydroxyl groups in depolymerization products (DP) remained higher than that of depolymerization‐coupling products (DCP), which may be attributed to the insufficient depolymerization of lignin, rendering the overall product composition close to that of pristine protected lignin. Despite the relatively low overall content of side‐chain hydroxyl groups in DCP, its monomeric products possess diol structures, which provide favorable prerequisites for polyurethane synthesis. Accordingly, DP and DCP were reacted with methylene diphenyl diisocyanate (MDI) and 1,6‐diisocyanatohexane (HDI), respectively, to prepare polyurethanes (Figure 7a). No polymeric product was obtained from the reaction of DP, whereas DCP reacted with MDI and HDI to form a powdery polymer PU‐1 and a sheet‐like polymer PU‐2, respectively. FT‐IR spectra confirmed the presence of abundant N–H bonds and carbonyl groups in both PU‐1 and PU‐2 (Figure 7b). Meanwhile, the signals of the aromatic rings in PU‐1 were greatly enhanced, which is consistent with the isocyanate monomer used and confirms the successful synthesis of polyurethanes.

The thermal properties of the resulting polyurethanes were further investigated using TG and DSC (Figure 7c). Both PU‐1 and PU‐2 were stable below 200 °C and showed significant decomposition above 280 °C, with maximum mass loss in the range of 335–340 °C. Despite their different structures, the two materials exhibited similar glass transition temperatures (*T*_g_ = 162–164° C), indicating comparably rigid polymer networks. These results further support the feasibility of using the aromatic diol monomers obtained in this work by one‐pot lignin depolymerization cross‐coupling as precursors for polyurethane synthesis.

## 3 Conclusion

In summary, we developed and applied a band‐engineered photo‐redox radical co‐matching strategy for integrated lignin‐depolymerization and in situ cross‐coupling reaction with aliphatic alcohols to produce unsymmetrical aromatic diols. By simultaneously generating aliphatic alcohol radicals and benzylic alcohol radicals, this strategy overcomes the intrinsic preference for homocoupling and enables selective cross‐coupling between reactions to produce unsymmetrical diols. Tuning the band structure of CdZnS photocatalytic system allows control over radical generation and fate, enabling one‐pot depolymerization and upgrading of native lignin to produce aromatic diols. The resulting aromatic diols can be directly utilized for polyurethane synthesis and further upgraded to fuel‐range cycloalkanes. Overall, this work identifies radical co‐matching as a useful approach for tandem lignin depolymerization upgrading and for the selective cross‐coupling of non‐equivalent, alcohol‐derived radicals.

## Author Contributions

**Hongji Li**: conceptualization, investigation, writing – original draft, methodology, formal analysis, supervision, resources, writing – review and editing, and project administration. **Jialing Ma**: conceptualization, investigation, writing – original draft, methodology, formal analysis, and writing – review and editing. **Xiaoling Wan**: conceptualization, investigation, writing – original draft, methodology, and writing – review and editing. **Yumei Liu**: conceptualization, investigation, writing – original draft, writing – review and editing, and formal analysis. **Chaofeng Zhang**: conceptualization, investigation, writing – original draft, methodology, visualization, writing – review and editing, and formal analysis. **Xiaoqin Si**: conceptualization, investigation, writing – original draft, validation, methodology, writing – review and editing, formal analysis, and supervision. **Xiaojun Shen**: conceptualization, investigation, writing – original draft, methodology, visualization, writing – review and editing, validation, formal analysis, supervision, and project administration. **Rajenahally V. Jagadeesh**: conceptualization, funding acquisition, writing – review and editing, and supervision.

## Conflicts of Interest

The authors declare no competing interest.

## Supporting information




**Supporting File**: More characterizations on catalysts, compounds, and other control experiments are provided in the Supporting Information.

## Data Availability

The data that support the findings of this study are available on request from the corresponding author. The data are not publicly available due to privacy or ethical restrictions.
