# Supplementary material for: Photocatalytic Lignin Depolymerization and Cross‐Coupling With Alcohols to Produce Unsymmetric Aromatic Diols
Source: Angew Chem Int Ed Engl. 2026 Jun 9;65(32):e2045478. doi: 10.1002/anie.2045478 (PMC13427220; doi:10.1002/anie.2045478)
Supplement: Supplementary file 1 — Supporting File: More characterizations on catalysts, compounds, and other control experiments are provided in the Supporting Information. [file ANIE-65-e2045478-s001.docx]

**Supporting Information for**

**Photocatalytic Lignin Depolymerization and Cross-Coupling with Alcohols to Produce Unsymmetric Aromatic Diols**

Hongji Li*^[a]^, Jialing Ma^[a]^, Xiaoling Wan^[a]^, Yumei Liu^[b]^, Chaofeng Zhang^[c]^, Xiaoqin Si*^[d]^, Xiaojun Shen*^[b]^, and Rajenahally V. Jagadeesh*^[b,e]^

[a] Prof. H. Li, J. Ma, X. Wan
College of Chemistry
Zhengzhou University
Zhengzhou 450001, China
E-mail: hongjili@zzu.edu.cn

[b] Dr. Y. Liu, Dr. X. Shen and Prof. R.V. Jagadeesh
Leibniz-Institut für Katalyse e.V.
Rostock, 18059, Germany
E-mail: xiaojun.shen@catalysis.de and jagadeesh.rajenahally@catalysis.de

[c] Prof. C. Zhang
College of Light Industry and Food Engineering.
Nanjing Forestry University
Nanjing 210037, China

[d] Prof. X. Si
School of Chemical Engineering.
Zhengzhou University
Zhengzhou, 450001, China
E-mail: sixiaoqin@zzu.edu.cn

[e] Prof. R.V. Jagadeesh
Nanotechnology Centre, Centre for Energy and Environmental Technologies.
VS ̌B-Technical University of Ostrava
Ostrava-Poruba, 70800, Czech Republic

**Materials**

Alcohols, lithium salts, cadmium acetate dihydrate, zinc acetate dihydrate, sodium sulfide, formaldehyde aqueous solution (37%), acetaldehyde aqueous solution (40%), phenol, guaiacol, 2,6-dimethoxyphenol, 2-bromo-4′-methoxyacetophenone, 5,5-dimethyl-1-pyrroline N-oxide, methanol-d_4_ and other solvents were of analytical grade and purchased from commercial suppliers such as Aladdin, Macklin, and Bidepharm without further purification. The synthesis of lignin models was performed according to the reported procedure.^[^[^1^](#_ENREF_1)^]^

**The** **preparation of Cd_x_Zn_1-x_S**

A series of Cd_x_Zn_1-x_S photocatalysts were synthesized by regulating the feed molar ratio of cadmium acetate dihydrate (Cd(CH_3_COO)_2_·2H_2_O) and zinc acetate dihydrate (Zn(CH_3_COO)_2_·2H_2_O). The detailed procedure for the hydrothermal preparation of Cd_0.3_Zn_0.7_S photocatalyst is as follows: 0.477 mmol of Cd(CH_3_COO)_2_·2H_2_O, 1.114 mmol of Zn(CH_3_COO)_2_·2H_2_O, and 1.591 mmol of sodium sulfide nonahydrate (Na_2_S·9H_2_O) were successively added into a beaker containing 30 mL of distilled water. After stirring for 1 h to form a homogeneous suspension, the mixture was transferred into a 100 mL Teflon-lined stainless steel autoclave and hydrothermally reacted at 160 °C for 12 h. Once the autoclave was naturally cooled to room temperature, the solid product was collected via filtration, washing and drying.

**The extraction of lignin samples**

**PL**. Acetaldehyde-protected balsa wood lignin was extracted according to the literature method.^[^[^2^](#_ENREF_2)^]^ Briefly, 5 g of balsa wood powder, 72 mL of 1,4-dioxane, 7.2 mL of acetaldehyde solution (40%), and 2.1 mL of HCl (35 wt%) were successively added into a 250 mL round-bottom flask. The reaction was carried out at 80 °C for 3 h under an argon atmosphere. After completion of the reaction, 2.1 g of NaHCO_3_ was added into the flask and stirred for 30 min to neutralize the excess hydrochloric acid. Subsequently, the mixture was filtered and washed with 1,4-dioxane to remove salts and wood powder residues, and the filtrate was collected. The filtrate was concentrated in vacuo at 50 °C, followed by dissolution with 20 mL of tetrahydrofuran to obtain a suspension. The suspension was subjected to centrifugation, and the supernatant was collected to remove a portion of the sugars. The collected liquid was added dropwise into a beaker containing 500 mL of deionized water under vigorous stirring to regenerate lignin precipitates. The product was filtered, air-dried, and collected, denoted as PL.

**UL**. The extraction procedure of unprotected lignin (UL) was the same as that for PL, except that 7.2 mL of acetaldehyde solution (40%) was replaced with 3 mL of pure water.

**Characterization methods of photocatalysts**

The XRD patterns of CdZnS catalysts were collected on Panalytical X'Pert3 Powder. The SEM images were collected on a German ZEISS Sigma 300. The HRTEM images were collected on FEI Tecnai F20. The ICP data was obtained on Agilent ICP-OES 511. The elemental and VB XPS were collected on Thermo Fisher ESCALAB 250Xi and the data were calibrated by C_1s_ (284.8 eV). The UV-vis spectra of four photocatalysts were collected on Shimadzu UV-2700i with BaSO_4_ as background. The photocurrent density measurements and cyclic voltammetry tests were performed on a CHI660E electrochemical workstation. As for the photocurrent tests, the three-electrode cell was composed of a working electrode (FTO coated by photocatalysts), a reference electrode (saturated calomel electrode, SCE) and a counter electrode (platinum sheet). The light source was a Kessil LED (λ_max_ = 427 nm) lamp and the test was run in an aqueous solution of 0.5 M Na_2_SO_4_ under an argon atmosphere. The fluorescence spectra were collected on Edinburgh FLS1000, and the excitation wavelength was 420 nm.

**Characterization methods of lignin samples and products**

The ^1^H-, ^13^C-, and 2D HSQC NMR spectra of compounds and lignin samples were measured on a Bruker AVIII 400 spectrometer (^1^H: 400 MHz, ^13^C: 101 MHz). The gel permeation chromatography (GPC) was measured on Agilent GPC 50. The FT-IR spectra of PU samples were collected on a Thermo Nicolet 380. The TG-DSC data of PU samples was measured on HITACHI STA200 (temperature scope, 30-800 °C; rate, 10 °C/min). The DSC data of PU samples was measured on HITACHI DSC200 (temperature range, −80-250 °C; rate, 10 °C/min).

**The procedure of the photocatalytic reaction**

In a typical procedure, a 10 mL quartz reactor equipped with a stir bar was loaded with substrate (0.05-0.1 mmol) or lignin (20 mg), photocatalyst (10 mg), metal salts (0-1 equiv.), H_2_O (0-200 μL) and aliphatic alcohol (2 mL). Then the atmosphere was switched to argon before sealing the reactor. This mixture was irradiated using Kessil PR160 LED lamps (λ_max_ = 427 nm, 40 W) for a certain time. After the reaction, the standard solution (naphthalene in isopropanol) was added. After filtration using a Nylon syringe ﬁlter, the solution was analyzed by gas chromatography (Shimadzu GC-2014C). The water displacement method was used to quantify the generated hydrogen gas.

Molar yields of products from the benzylic alcohol or lignin model reaction were calculated as

$\text{Yield }\left( \text{i} \right)\text{=}\frac{\text{n}\left( \text{i} \right)}{\text{n}\left( \text{s} \right)}\text{×100\%}$ (1)

where *n*(*i*) and *n*(*s*) are the moles of product *i* and the initial substrate, respectively. Mass yields of aromatic monomer products from lignin samples were calculated as

$\text{Yield }\left( \text{i} \right)\text{=}\frac{\text{m}\left( \text{i} \right)}{\text{m}\left( \text{l} \right)}\text{×100\%}$ (2)

where *m*(*i*) and *m*(*l*) are the mass of product *i* and the mass of initial lignin, respectively.

**The photocatalyst recycling procedure**

After the reaction, isopropanol in the system was first removed via vacuum rotary evaporation. Subsequently, dichloromethane was added to fully dissolve the organic components and uniformly disperse the catalyst. The mixture was then separated by centrifugation and washed repeatedly with dichloromethane to remove the organic substances. Finally, the catalyst was recovered after drying by vacuum rotary evaporation and used for the next cycle of reaction.

**The preparation of NiMoO_x_ catalyst for hydrodeoxygenation reactions**

NiMoO_x_ catalyst was prepared through the hydrothermal-reduction method, and the detailed process was shown below. Typically, Ni(NO_3_)_2_·6H_2_O and Na_2_MoO_4_·2H_2_O were dissolved in deionized water, which was then transferred into a sealed autoclave and heated at 160 °C for 6 h. After the hydrothermal treatment, the separated solid was washed with ethanol and water, and then it was calcined at 500 °C for 2 h in air with the heating rate of 5 °C min^-1^ to obtain the catalyst precursor. Further, this precursor was reduced at 300 °C for 2 h in H_2_ with the heating rate of 3 °C min^-1^ and then cooled down to the room temperature.

**The preparation of PU samples**

***Pretreatment****.* The lignin reaction solution was first centrifuged to collect the supernatant and remove the catalyst. The obtained supernatant was subjected to vacuum rotary evaporation to afford a powdery solid. Subsequently, the solid was washed repeatedly with deionized water via centrifugation to eliminate pinacol and inorganic salts, followed by vacuum rotary evaporation to obtain the product containing only lignin-related derivatives.

Polyurethane was synthesized according to the method described in the literature,^[^[^3^](#_ENREF_3)^]^ with the detailed procedure as follows. 120 mg of lignin reaction product was added into a 20 mL glass vial containing 10 mL of tetrahydrofuran (THF). After complete dissolution, 3.4 mg of DABCO was introduced, and the system was purged with argon. Then 0.5 mmol of diphenylmethane diisocyanate (MDI) (or hexamethylene diisocyanate, HDI) was added. The mixture was sealed and stirred at 30 °C for MDI (or 50 °C for HDI) for 24 h. After completion of the reaction, the mixture was separated by centrifugation, washed with methanol three times, dried via vacuum rotary evaporation, and the final product was collected.

**Catalyt charectrization**


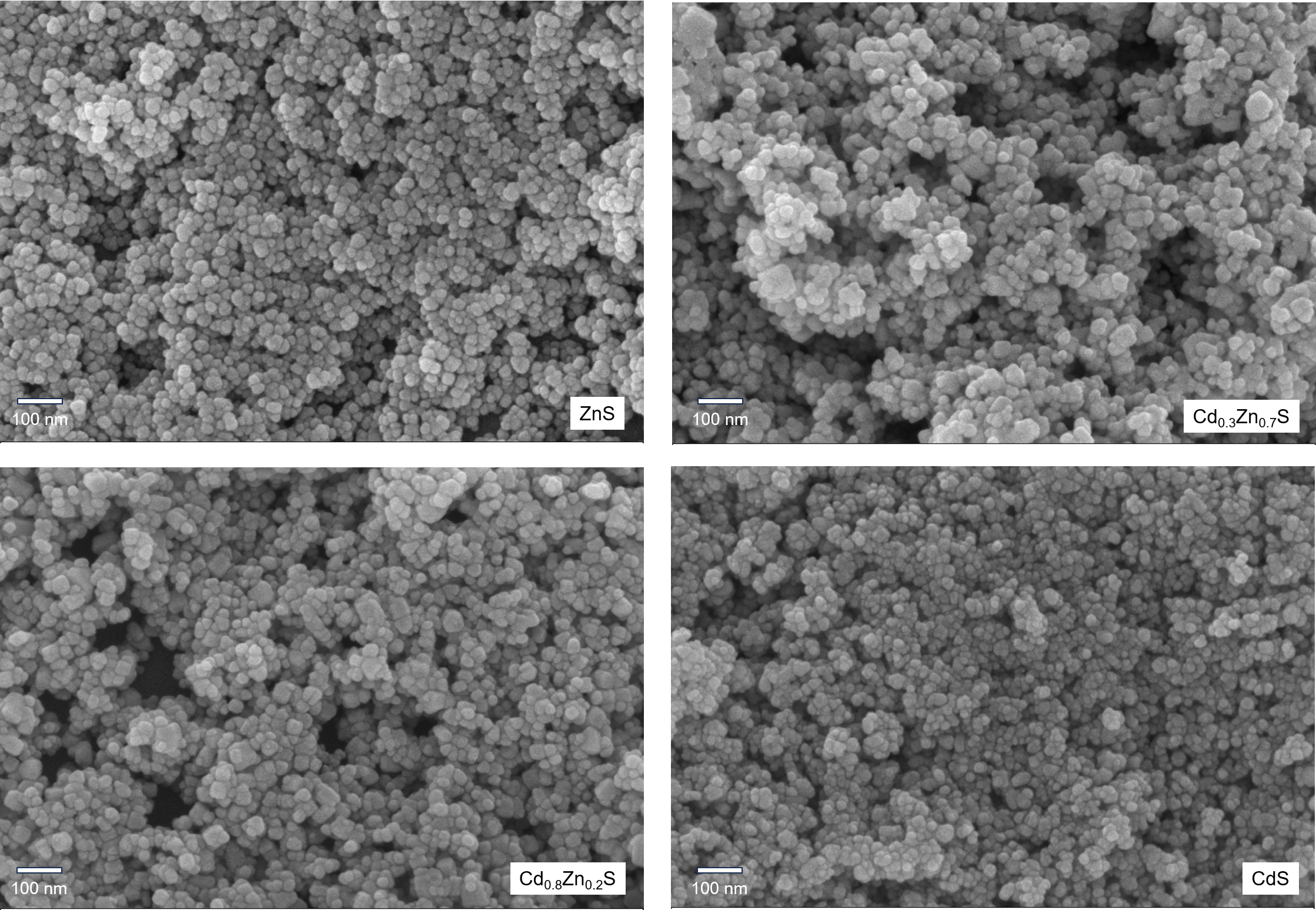


**Figure S1.** SEM images of prepared Cd_x_Zn_1-x_S catalysts.


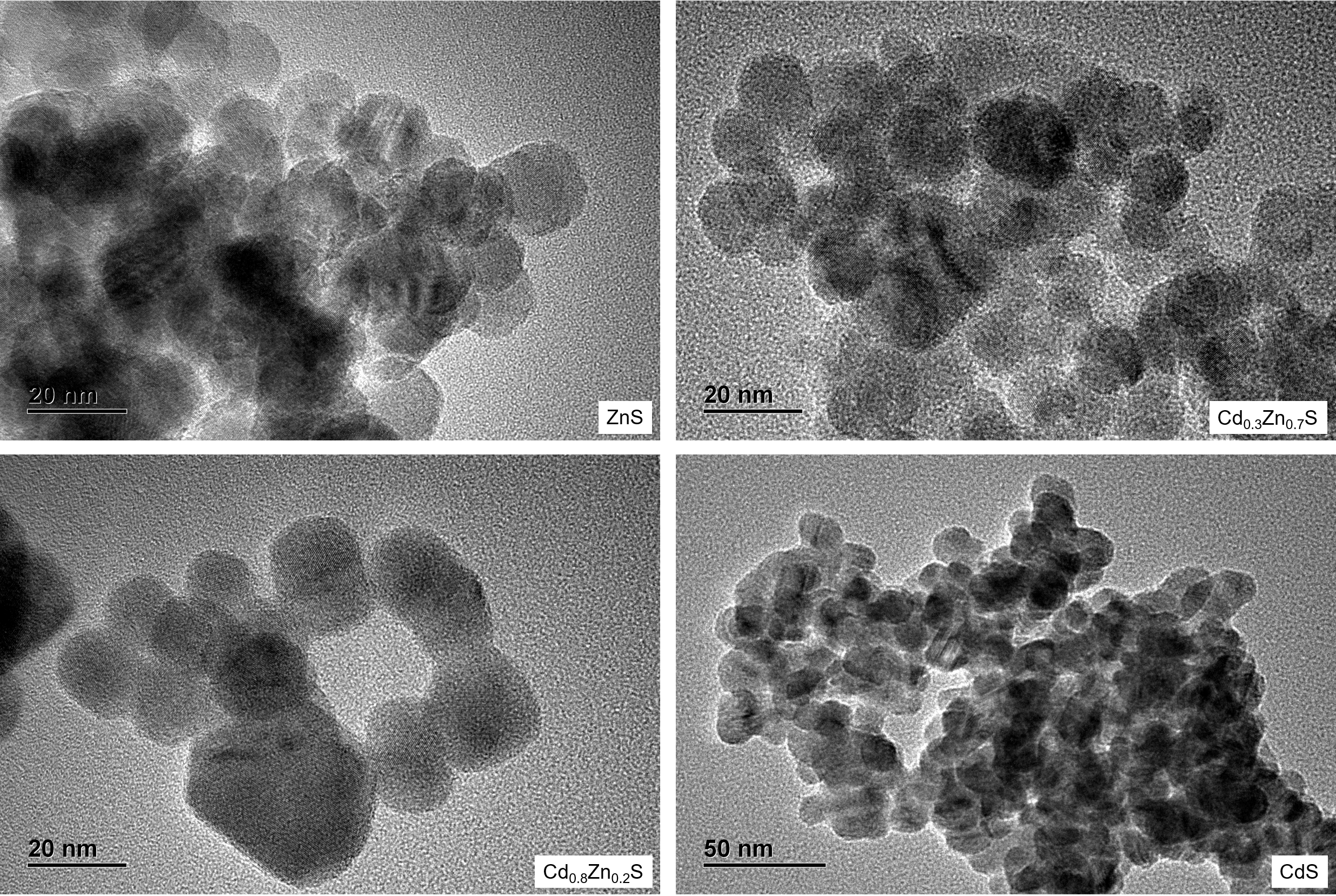


**Figure S2.** TEM images of prepared Cd_x_Zn_1-x_S catalysts.


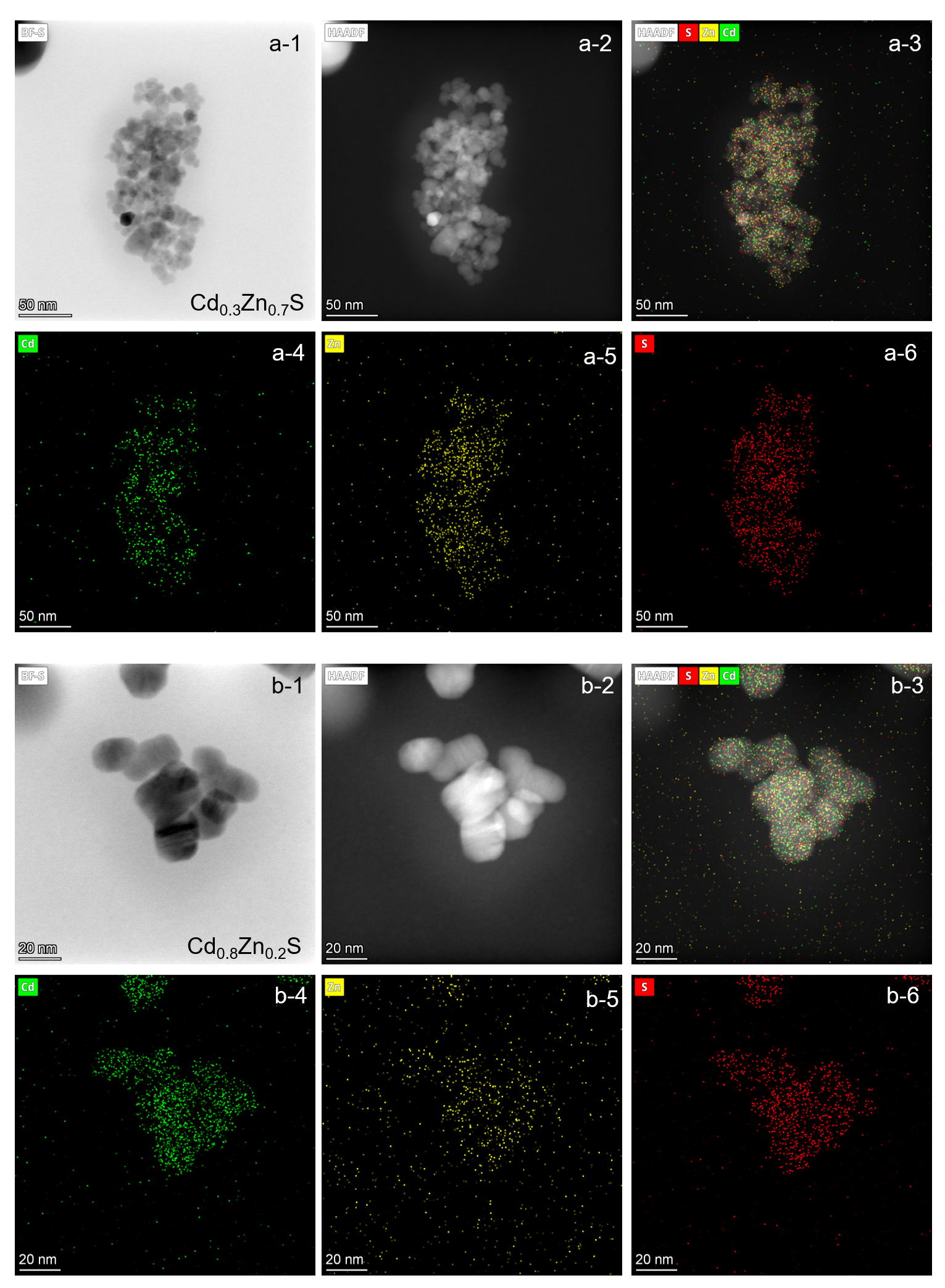


**Figure S3.** HAADF-STEM and EDS mapping of Cd_0.3_Zn_0.7_S and Cd_0.8_Zn_0.2_S.


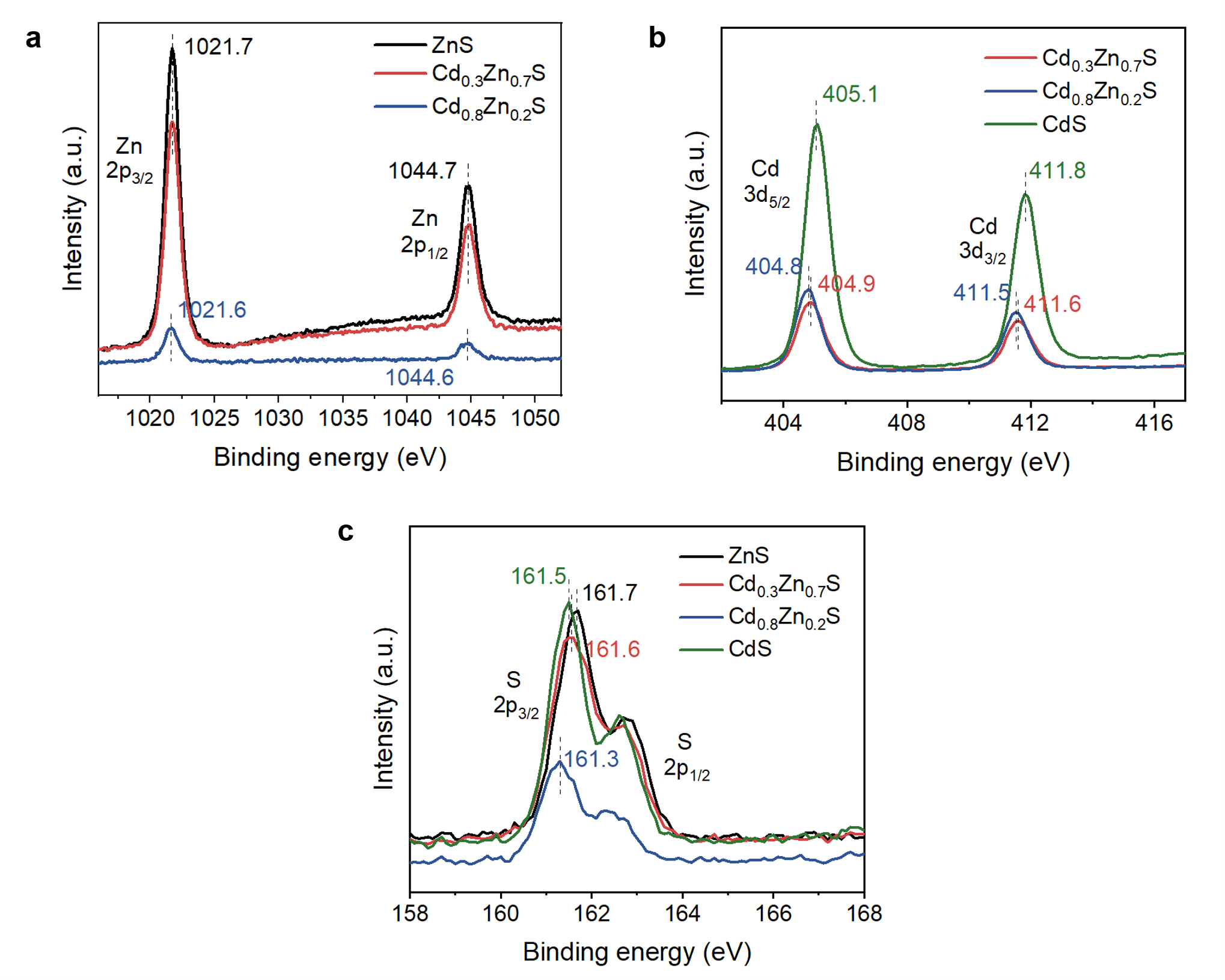


**Figure S4.** XPS analysis of Cd_x_Zn_1-x_S photocatalysts.


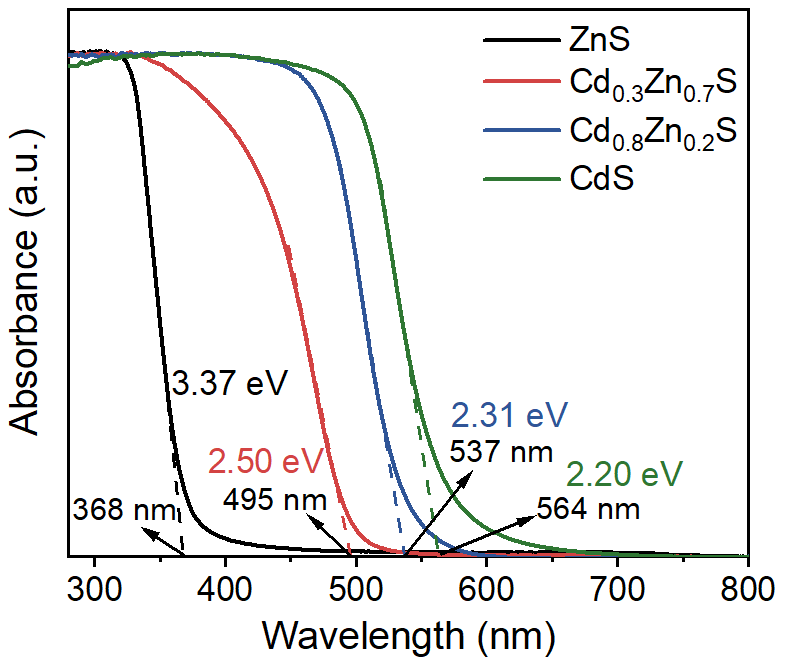


**Figure S5.** UV-vis absorption of the prepared Cd_x_Zn_1-x_S.


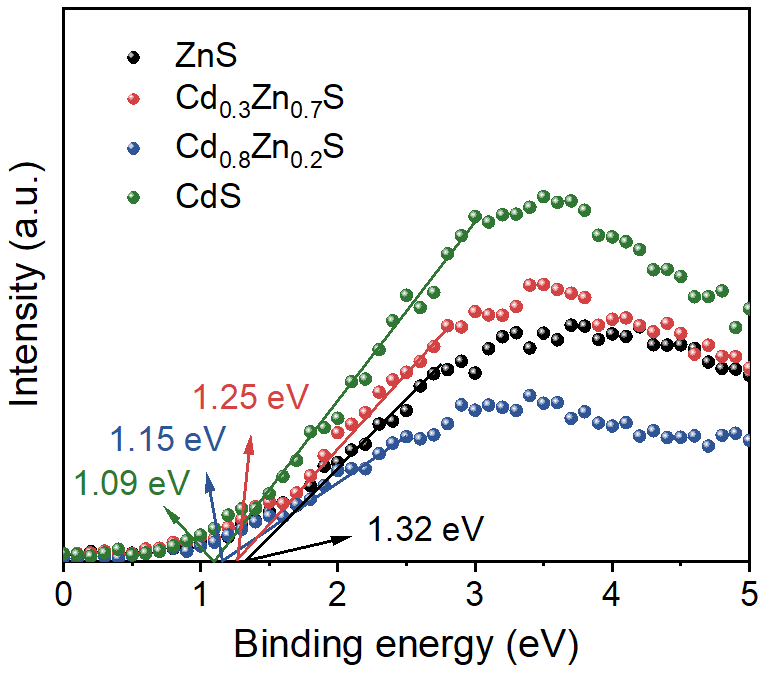


**Figure S6.** VB XPS analysis of the prepared Cd_x_Zn_1-x_S.

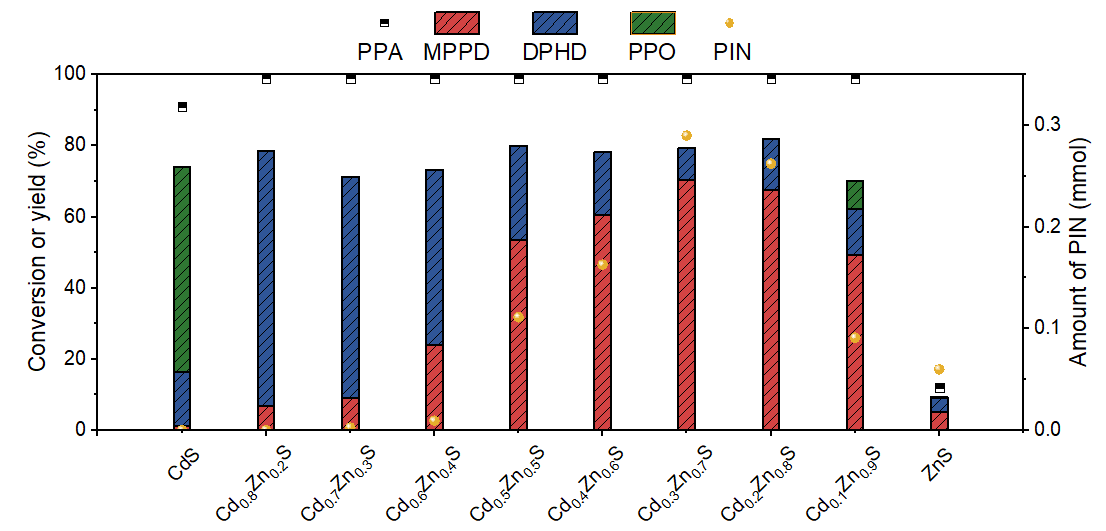


**Figure S7.** Effect of ratio of Cd/Zn on the reaction selectivity in the transformation of PPA in IPA.

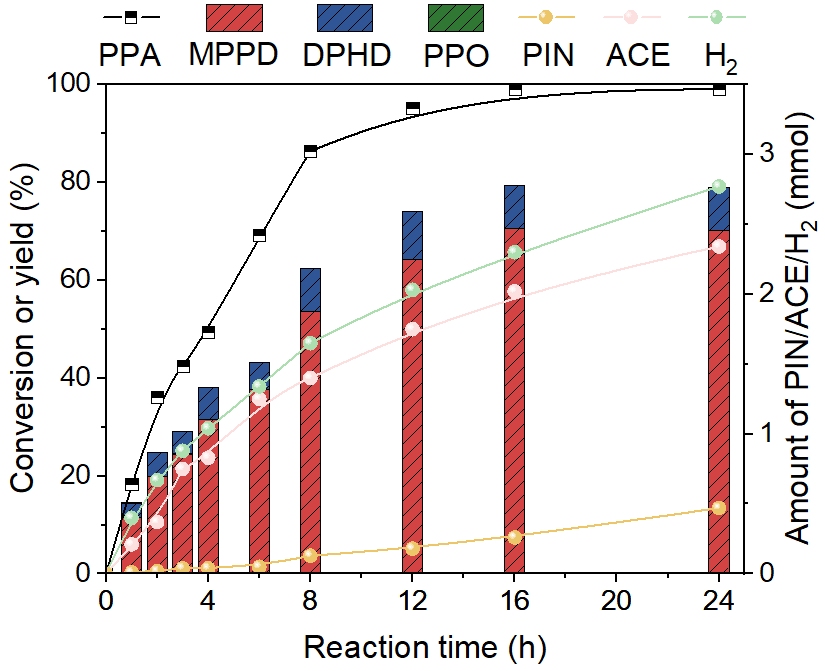


**Figure S8.** Time course of photocatalytic dehydrogenative coupling of 1-phenylpropanol with isopropanol on Cd_0.3_Zn_0.7_S.


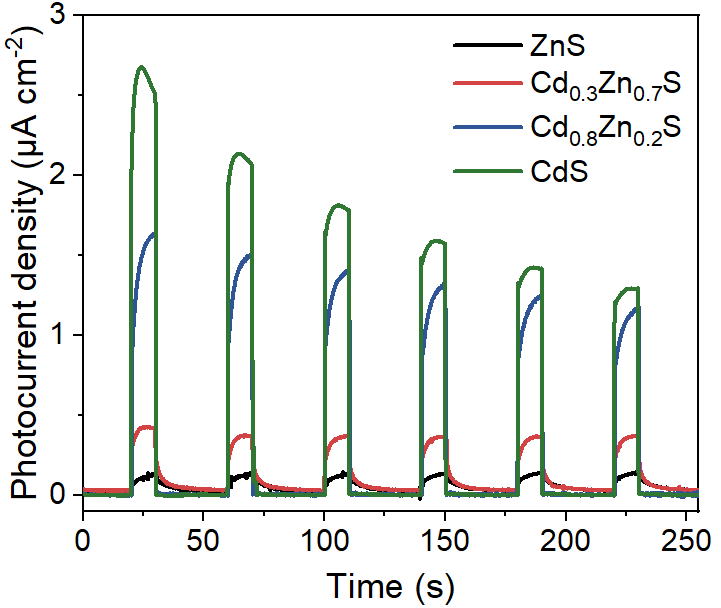


**Figure S9.** Transient photocurrent tests of prepared Cd_x_Zn_1-x_S under light irradiation (427 nm).


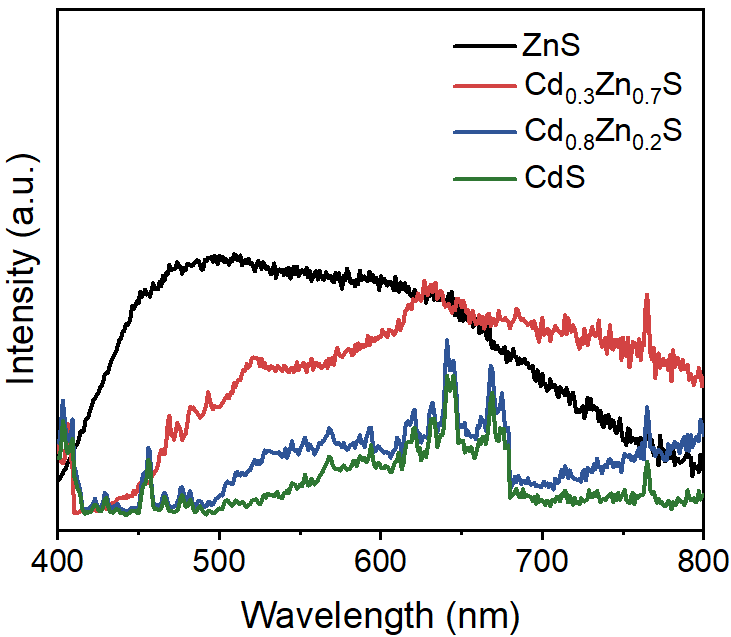


**Figure S10.** Photoluminescence steady-state fluorescence spectroscopy tests of the prepared Cd_x_Zn_1-x_S. Condition: λ_excitation_ = 370 nm.

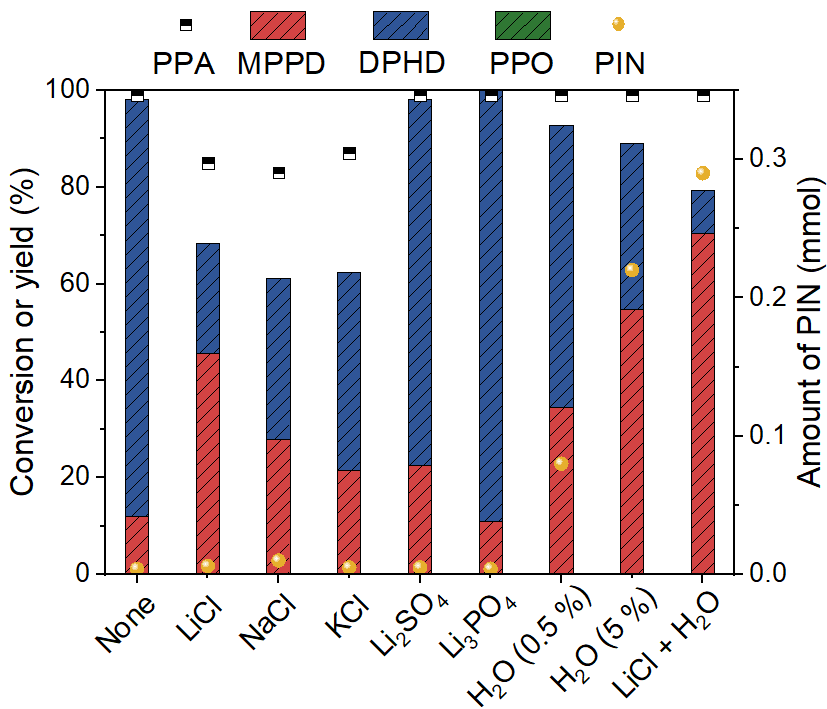


**Figure S11.** Effect of salt and water as additives on the photocatalytic performance of Cd_0.3_Zn_0.7_S in the cross-coupling reaction between PPA and IPA.

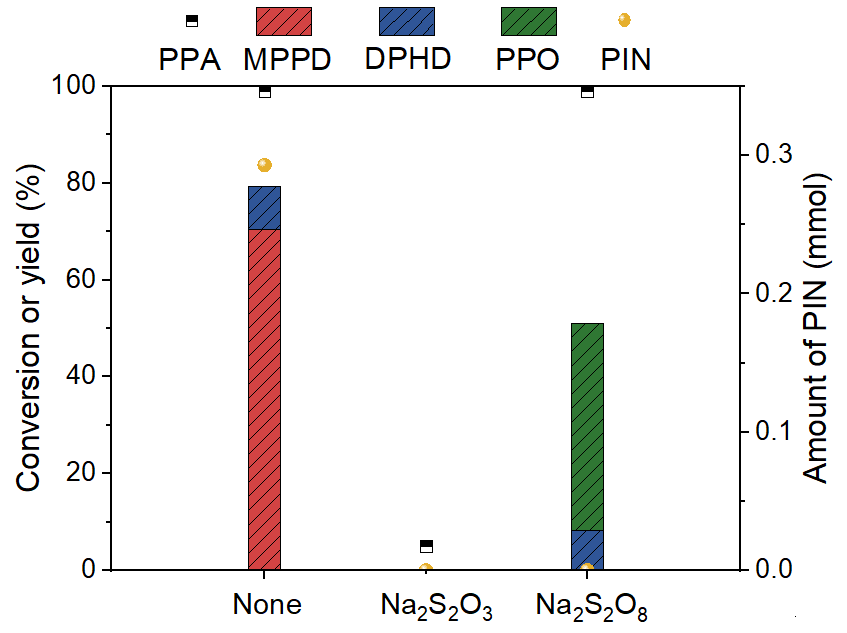


**Figure S12.** Effect of other additives on the cross-coupling reaction between PPA and IPA.

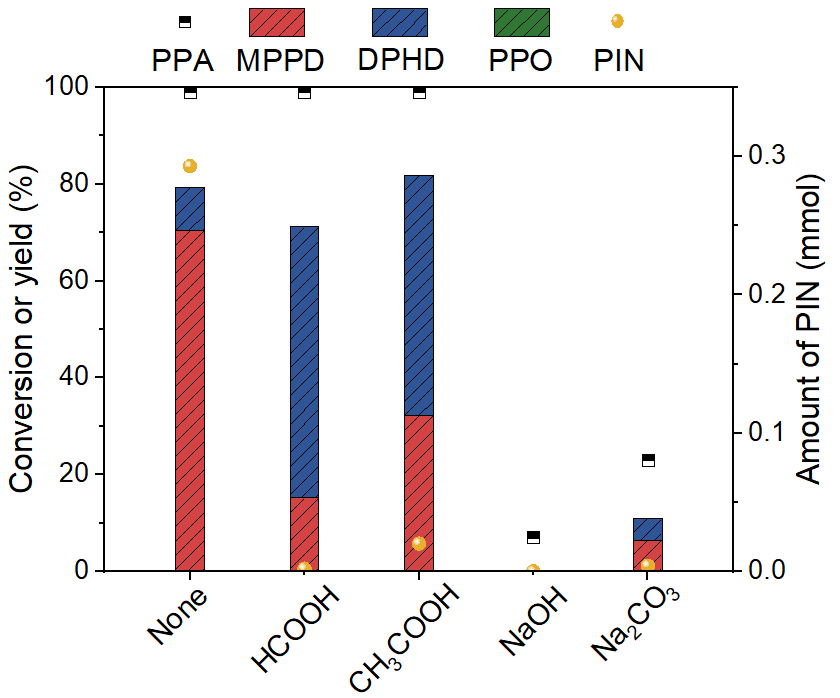


**Figure S13.** Effect of acid/base on the photocatalytic transformation of PPA in IPA.


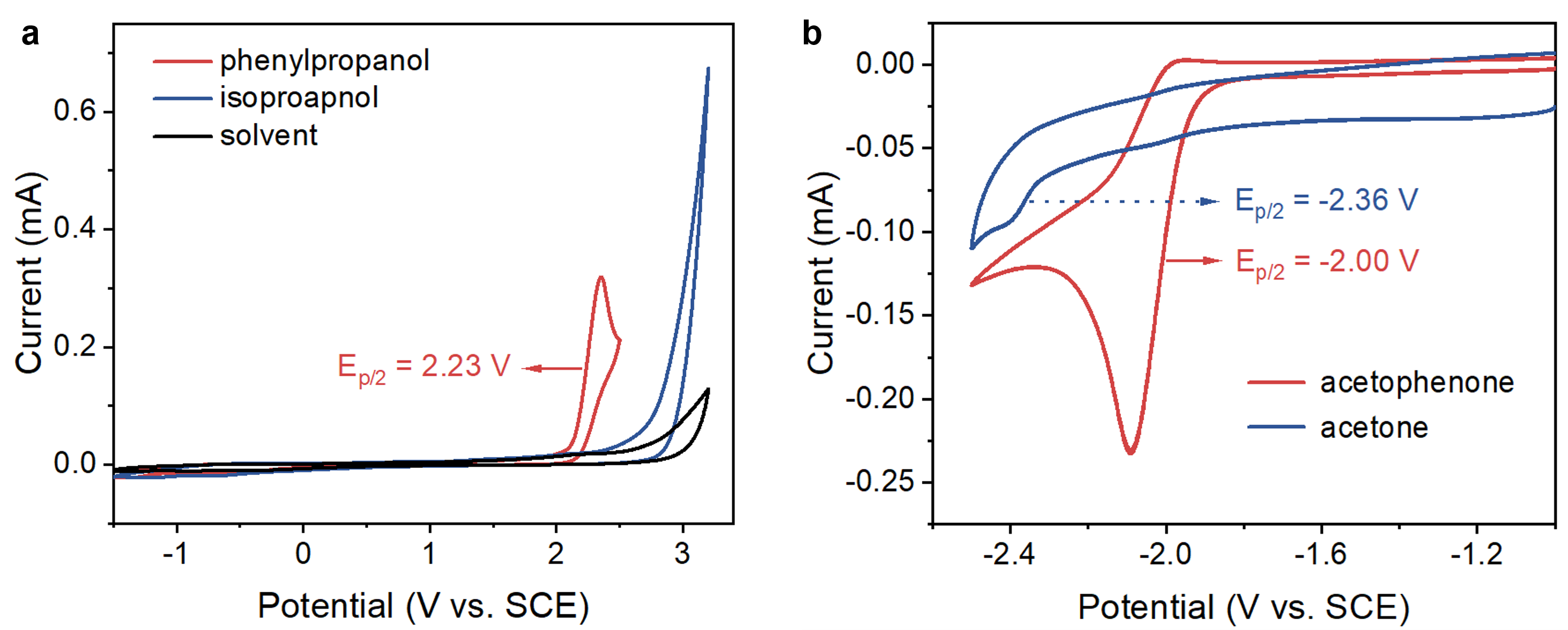


**Figure S14.** Cyclic voltammetry tests of different substrates. (a) The oxidation potentials of phenylpropanol and isopropanol. (b) The reduction potentials of acetophenone and acetone. Conditions: *n*Bu_4_NPF_6_ as the electrolyte (0.1 M), CH_3_CN as the solvent, glassy carbon as the working electrode, Pt sheet as the counter electrode, saturated calomel electrode as the reference electrode, argon atmosphere.

*Calculation details for the apparent quantum yield.*

*I*_0_ = P/*E*(427 nm)/*N*_A_ = Avg. Intensity × Area × *λ*/*h*c/*N*_A_ = (111 mW/cm^2^)×(4.38 cm^2^)/(6.626×10^-34^ J·s)/(299792458 m/s)×(427 ×10^-9^ m)/(6.022×10^23^ mol^-1^) = 1.74 ×10^-3^ mmol/s = 6.26 mmol/h

If all benzylic alcoholic radicals were generated through simple electron oxidation of benzylic alcohol, then one hole was needed for the formation of a radical.

ζ = 2n_(MPPD)_/(*I*_0_ × t)× 100% = 2× 0.07 mmol/(6.26 mmol/h × 16 h) × 100% =0.14%

If all benzylic alcoholic radicals were generated through simple electron reduction of benzylic alcohol, then two holes were needed for the formation of a radical.

ζ = 3n_(MPPD)_/(*I*_0_ × t)× 100% = 3× 0.07 mmol/(6.26 mmol/h × 16 h) × 100% =0.21%

The practical scenario is situated between these two extreme conditions. Therefore, the value of ζ is 0.14-0.21%.

**Figure S15.** The unsuccessful alcohols for cross-coupling with PPA.

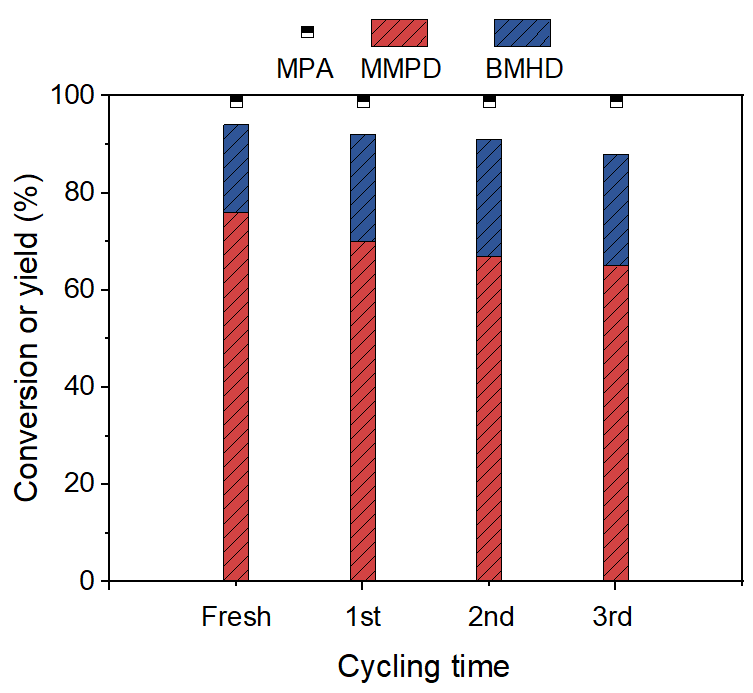


**Figure S16.** The catalyst cycling tests.

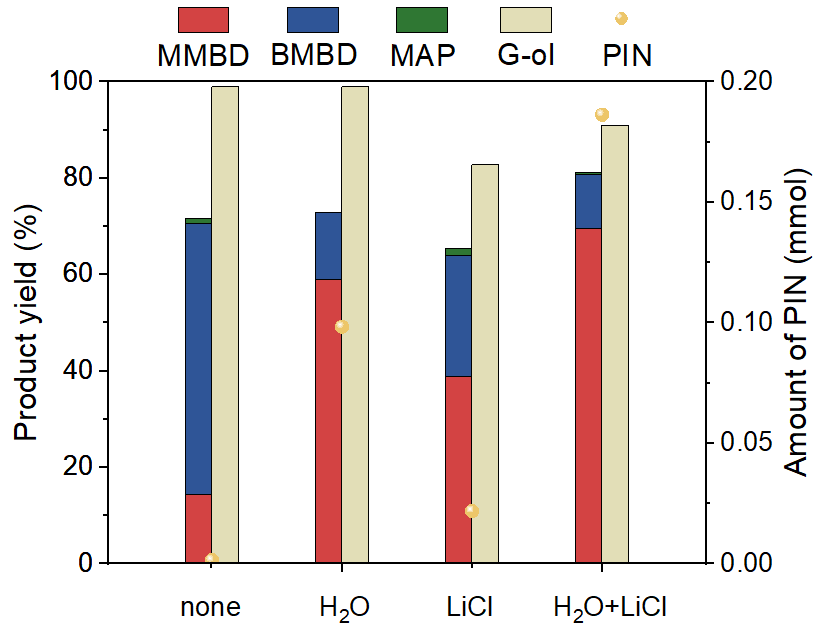


**Figure S17.** Effect of additives on photocatalytic transformation of lignin model in isopropanol.

i The GC-MS analysis of

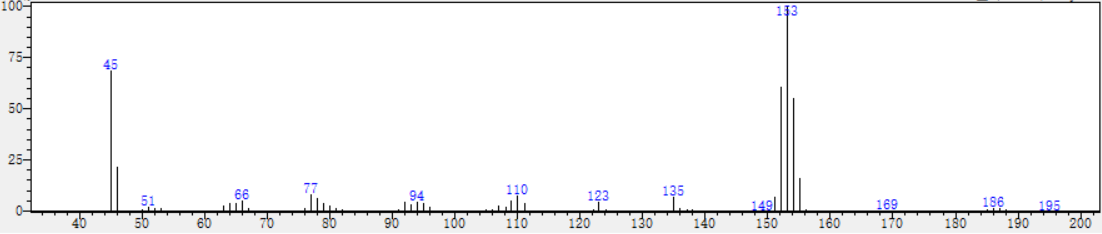


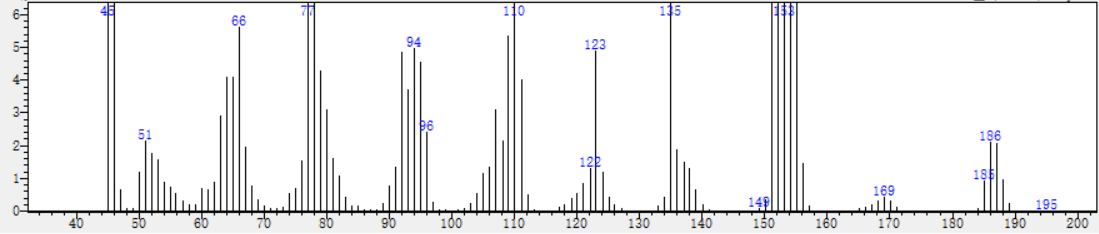


ii The GC-MS analysis of

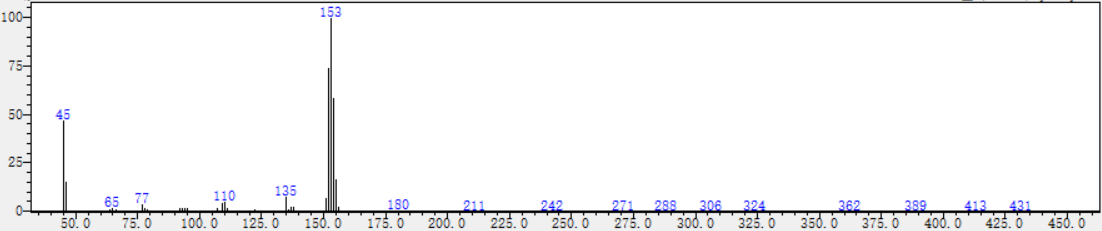


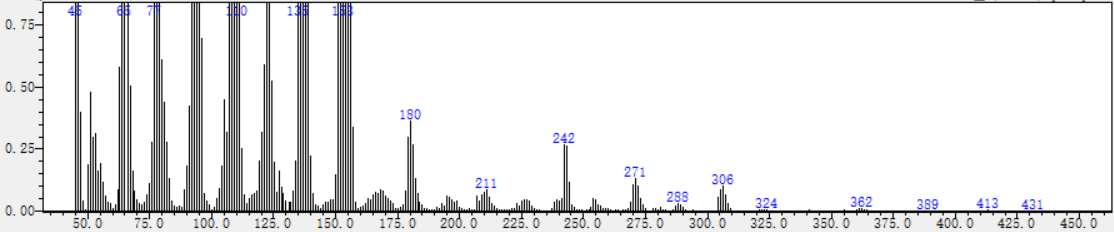


**Figure S18.** GC-MS analysis of the reaction of lignin model in Methanol-d_4_


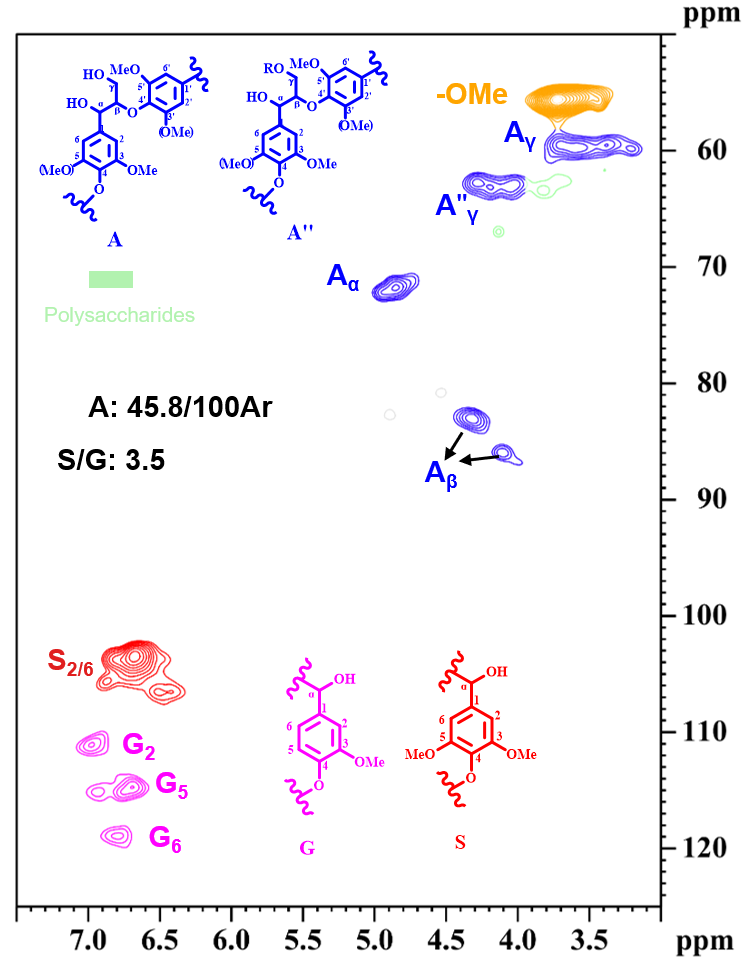


**Figure S19.** The 2D HSQC NMR spectrum of unprotected lignin.


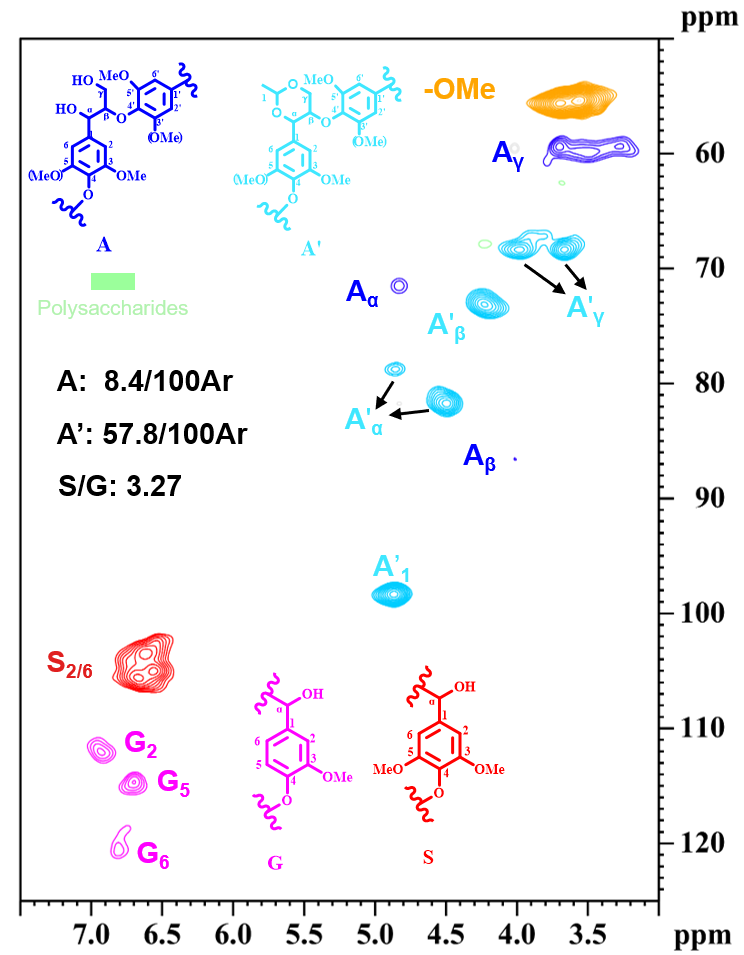


**Figure S20.** The 2D HSQC NMR spectrum of acetaldehyde-protected lignin.


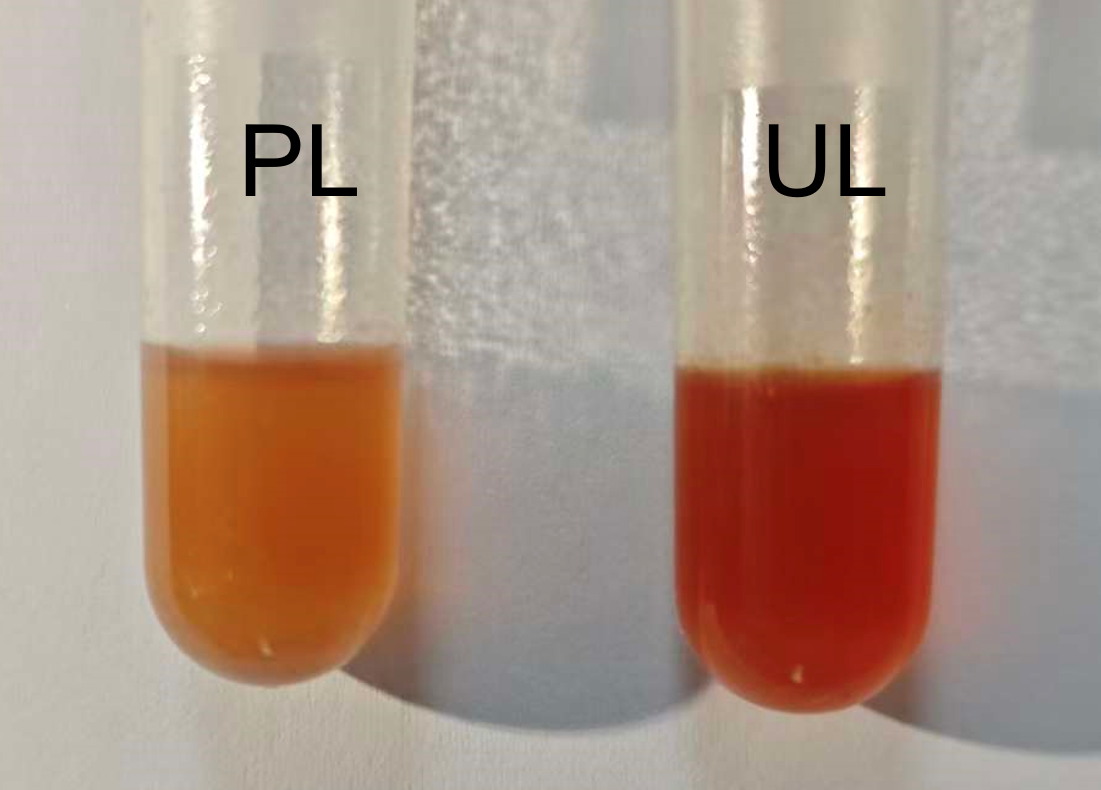


**Figure S21**. The digital photographs of PL and UL samples dissolved in isopropanol.

**Figure S22.** The photocatalytic transformation of other lignin samples in isopropanol.

The hydrodeoxygenation (HDO) of coupling products into cycloalkanes

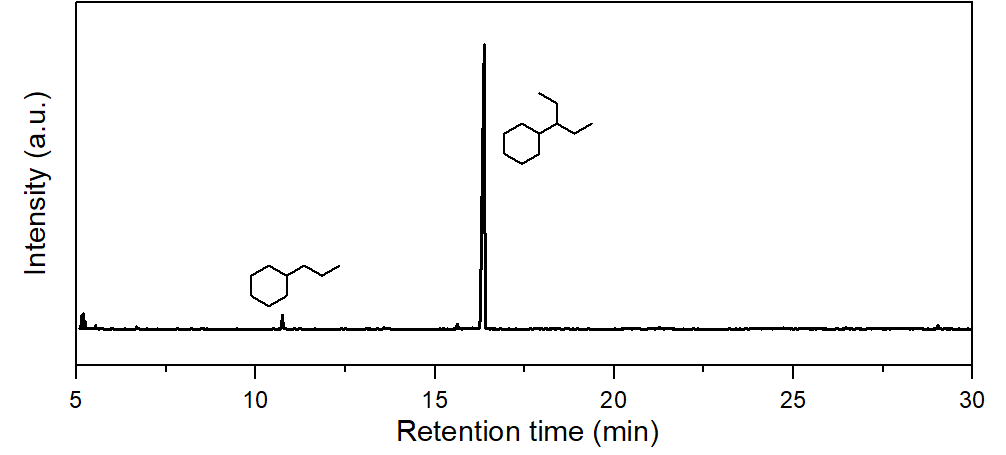


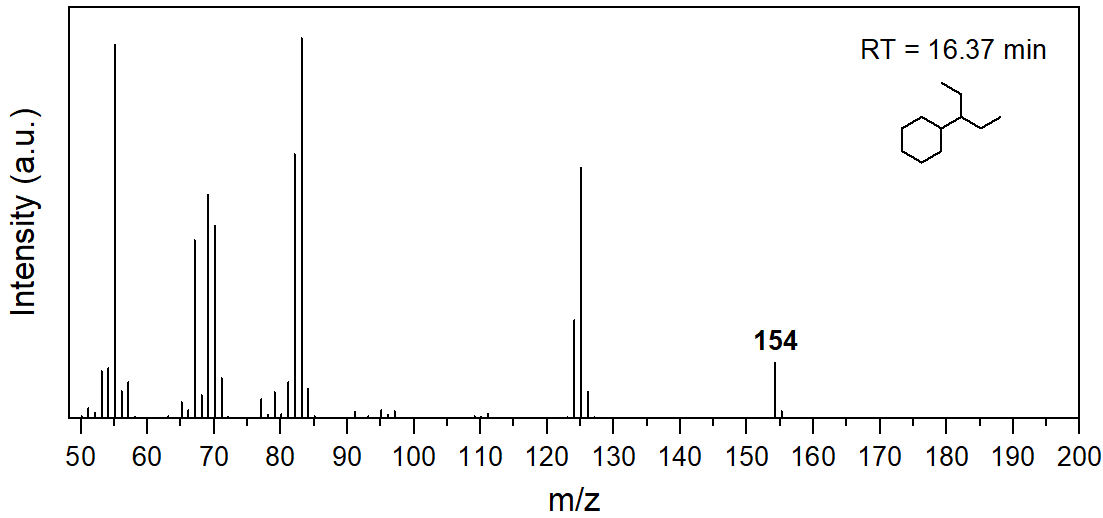


**Figure S23.** The HDO of C_11_ monomer and corresponding GC-MS analysis.

Conditions: 50 mg of substrate, 50 mg of catalyst, 10 mL cyclohexane, 220 ℃, 40 bar H_2_, 6 h.

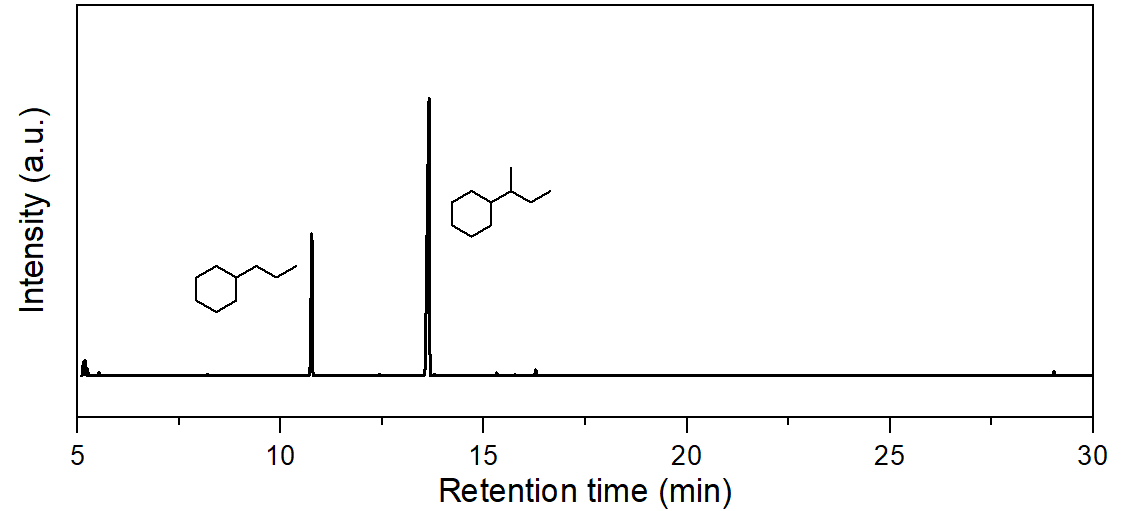


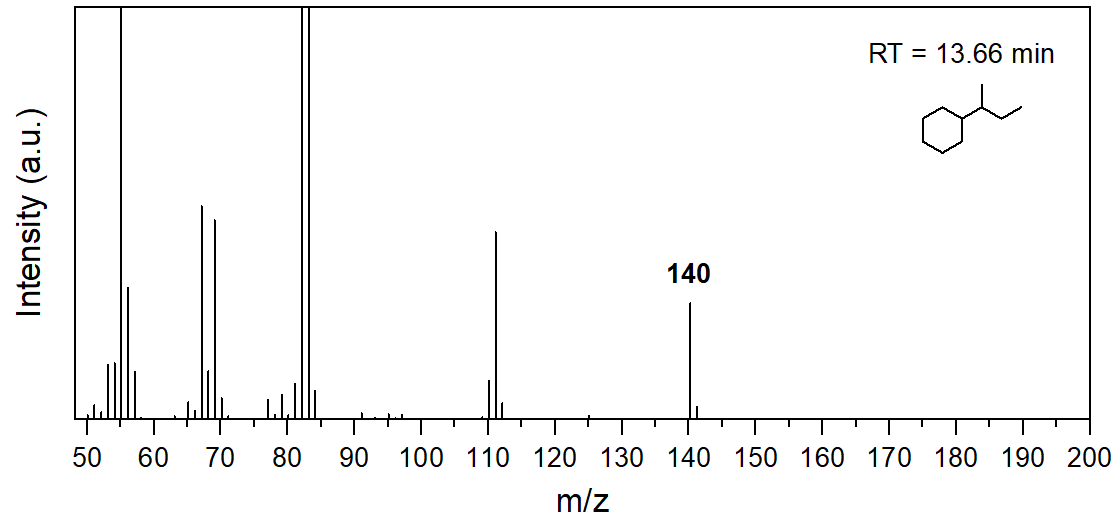


**Figure S24.** The HDO of C_10_ monomer and corresponding GC-MS analysis.

Conditions: 50 mg of substrate, 50 mg of catalyst, 10 mL cyclohexane, 220 ℃, 40 bar H_2_, 6 h.

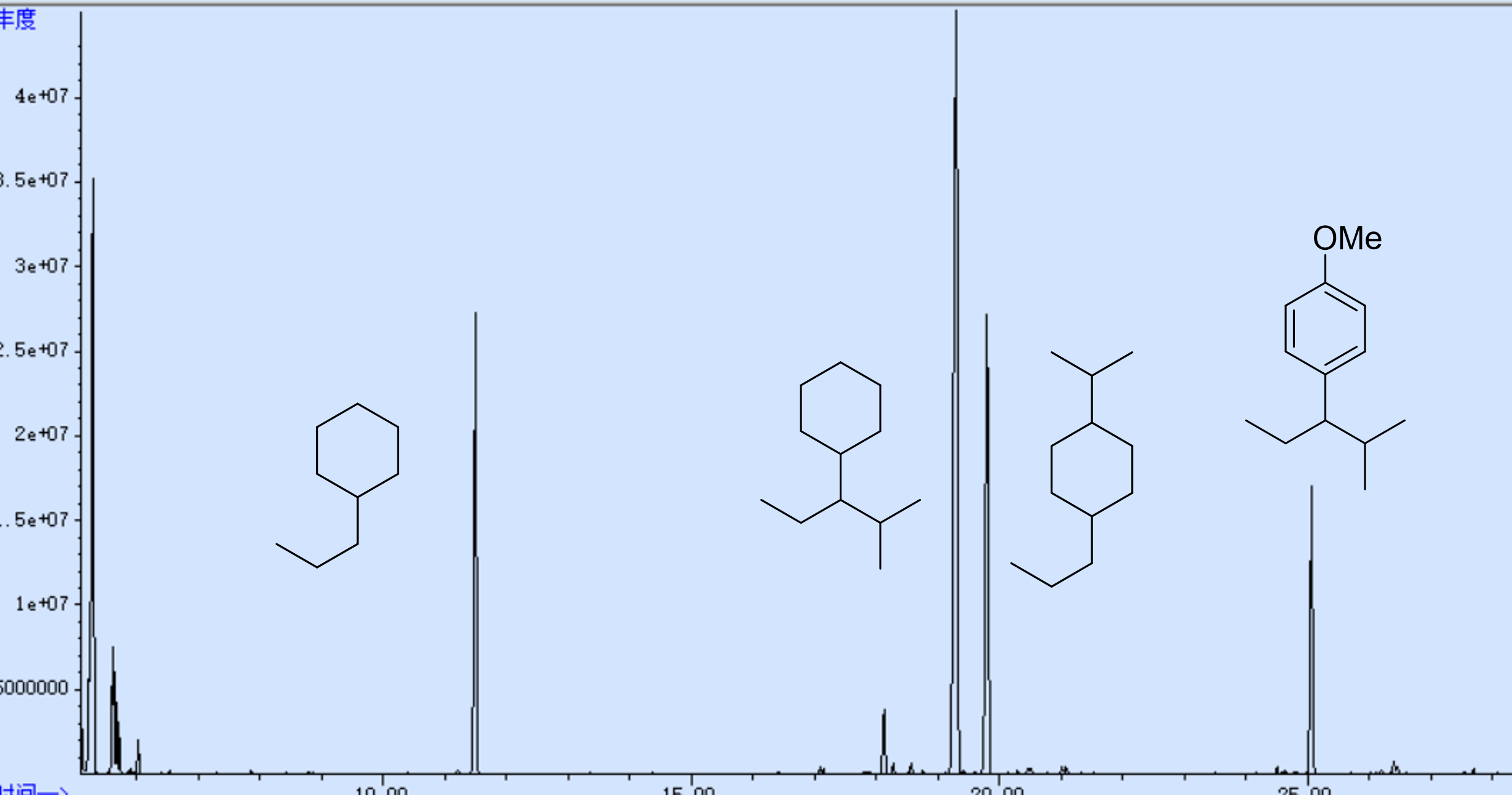


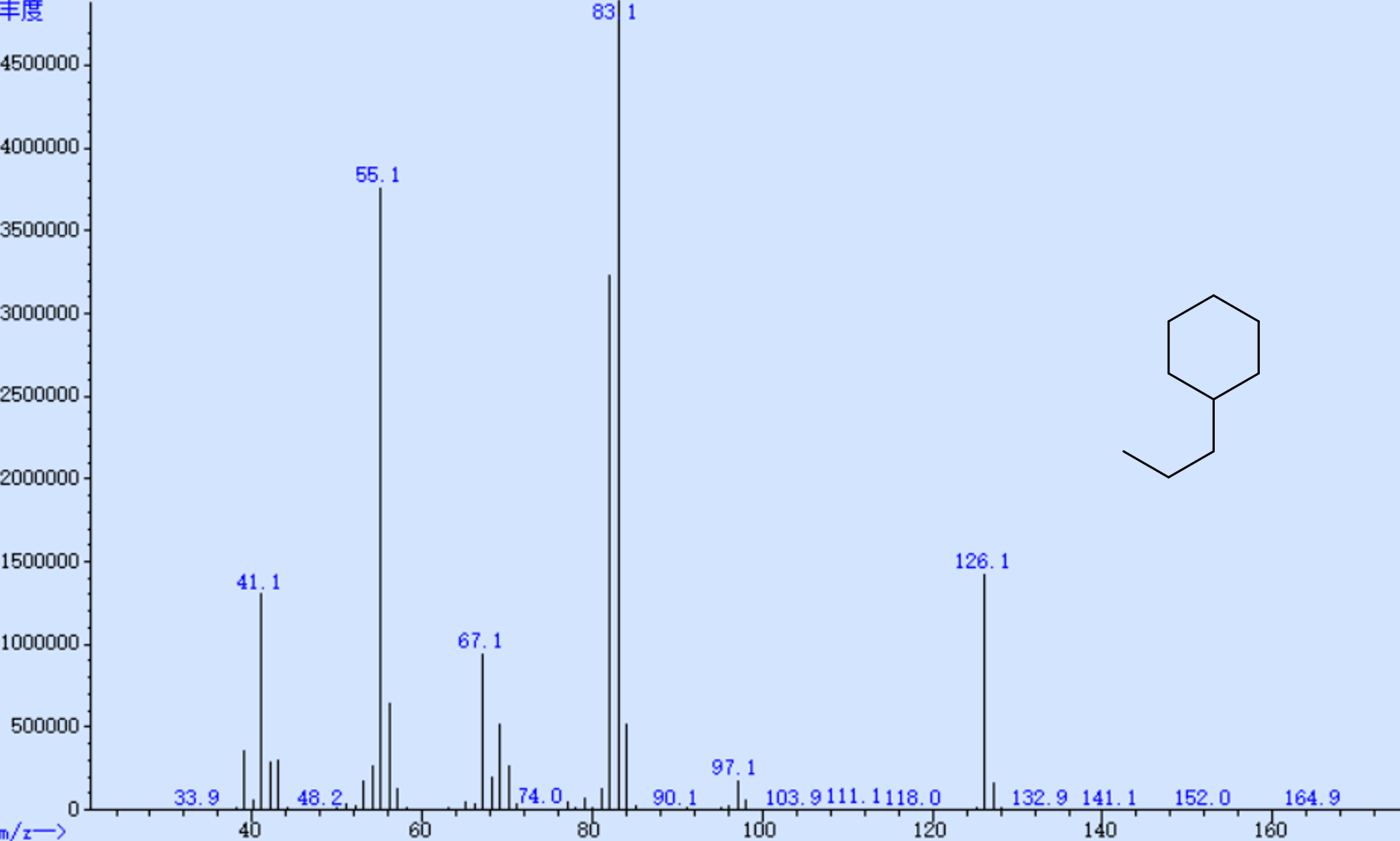


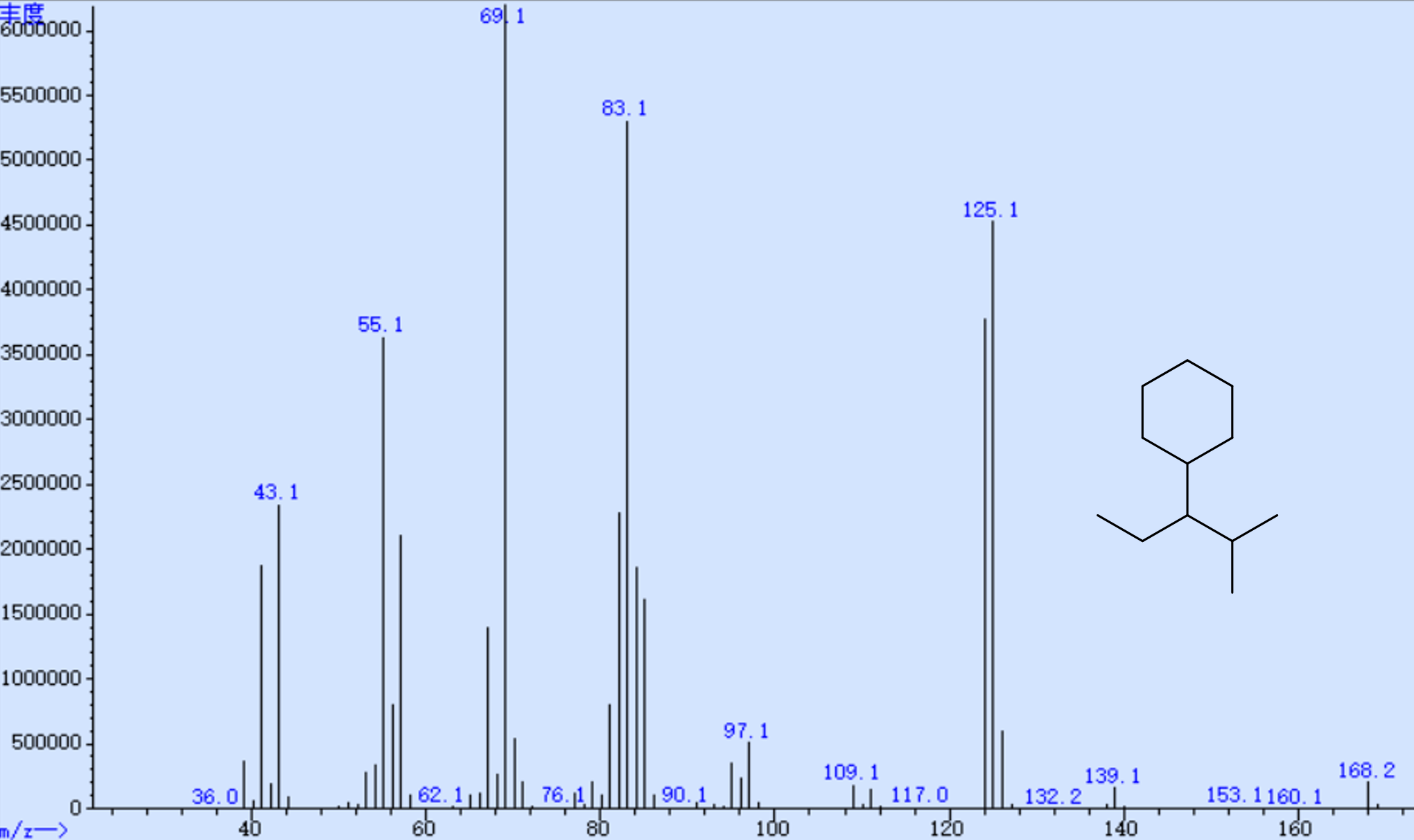


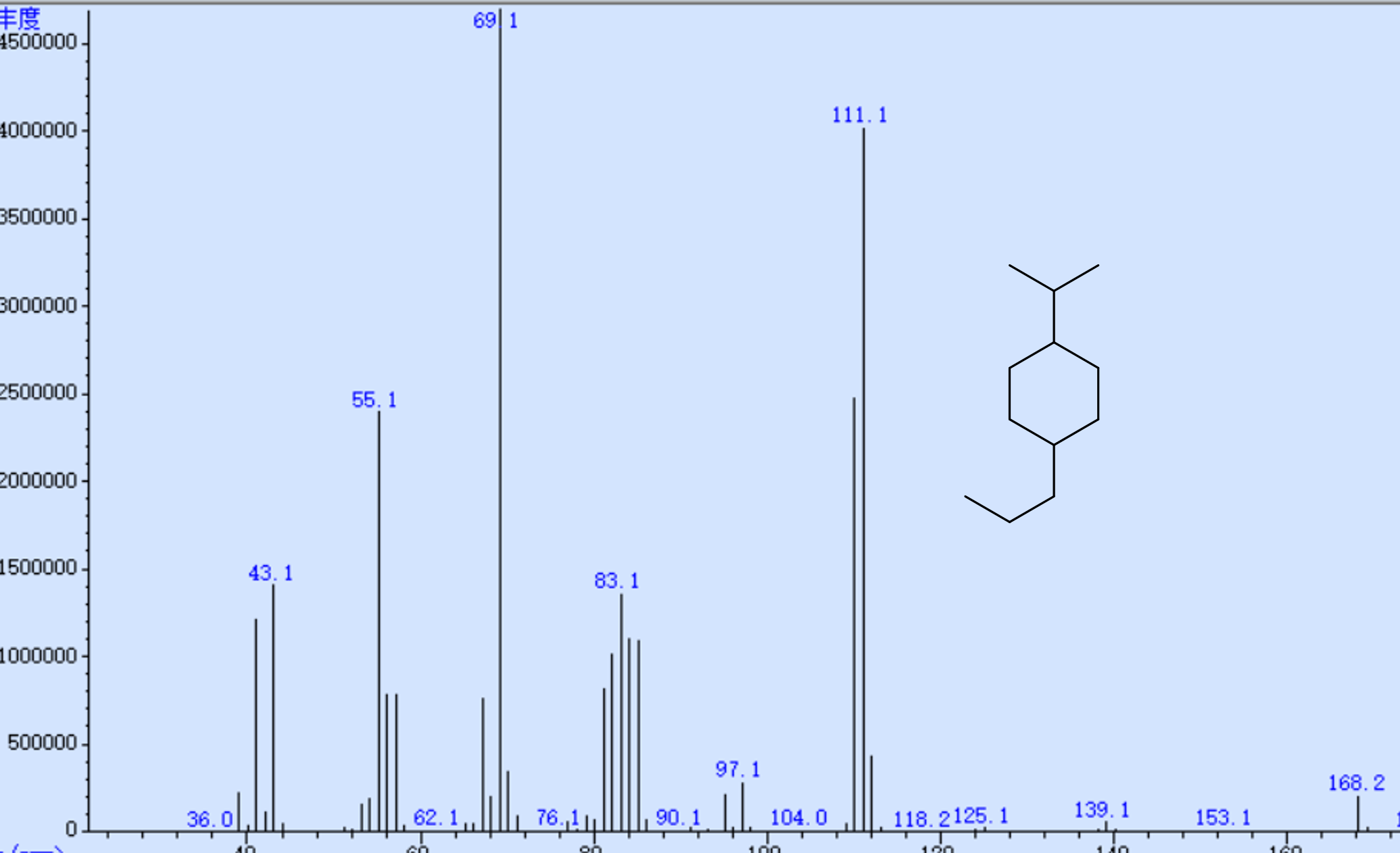


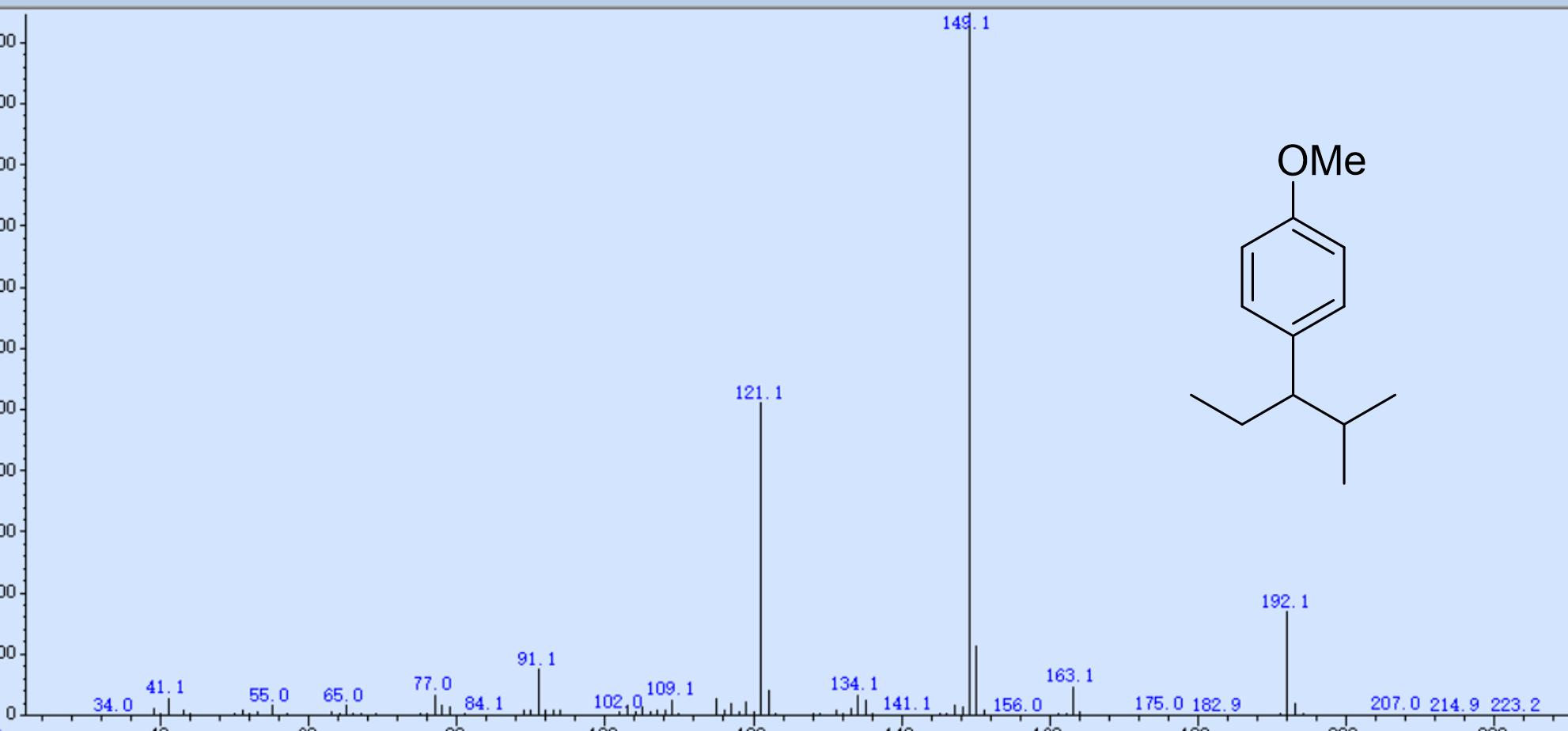


**Figure S25.** The HDO of C_12_ monomer and corresponding GC-MS analysis.

Conditions: 50 mg of substrate, 50 mg of catalyst, 10 mL cyclohexane, 250 ℃, 40 bar H_2_, 6 h.


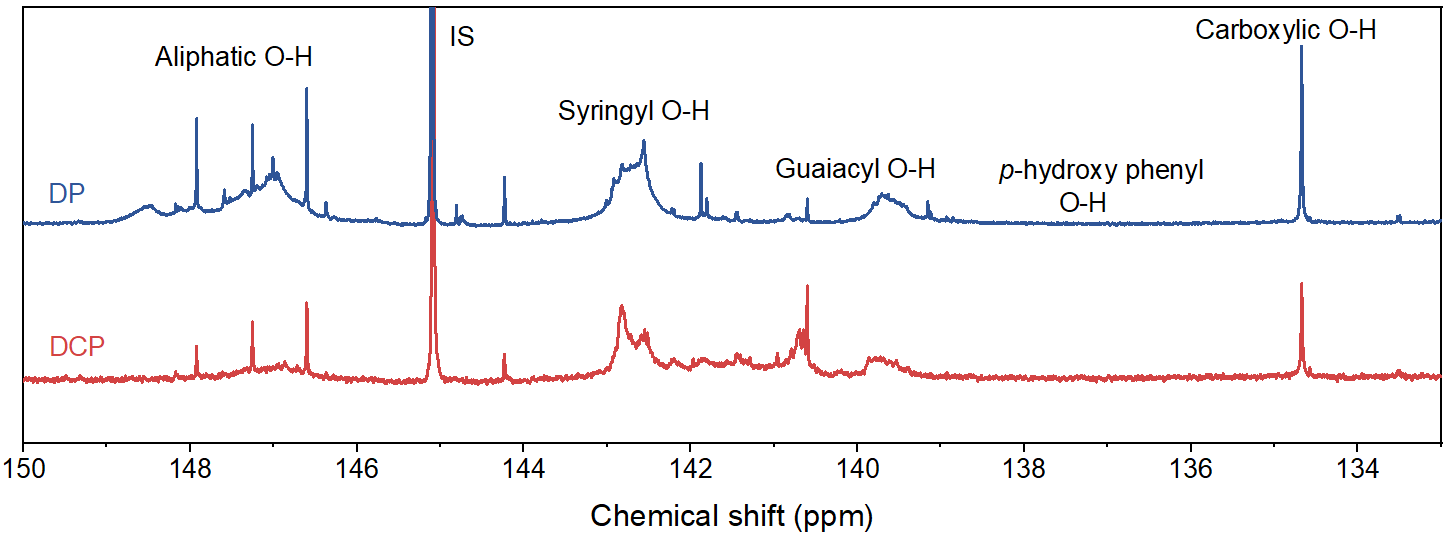


**Figure S26.** The detection of hydroxyl groups in lignin depolymerization products by ^31^P NMR.

**NMR and HRMS data of products**

Colorless oil. ^1^H NMR (400 MHz, CDCl_3_) δ 7.44 (d, *J* = 7.6 Hz, 2H), 7.33 (t, *J* = 7.6 Hz, 2H), 7.25 (t, *J* = 7.2 Hz, 1H), 2.28 (s, 1H), 2.20 (dd, *J* = 14.2, 7.3 Hz, 1H), 1.98 (dq, *J* = 14.2, 7.3 Hz, 1H), 1.27 (s, 3H), 1.06 (s, 3H), 0.73 (t, *J* = 7.4 Hz, 3H). ^13^C NMR (101 MHz, CDCl_3_) δ 141.73, 127.68, 127.29, 126.71, 81.29, 75.70, 26.93, 25.69, 24.60, 7.96. HRMS (ESI) *m/z* calcd for ([C_12_H_18_O_2_]+Na)^+^ [M+Na]^+^: 217.1204, found 217.1196.

Colorless oil. ^1^H NMR (400 MHz, CDCl_3_) δ 7.48 (d, *J* = 7.6 Hz, 2H), 7.32 (t, *J* = 7.5 Hz, 2H), 7.25 (t, *J* = 7.2 Hz, 1H), 2.41 (s, 2H), 1.63 (s, 3H), 1.25 (s, 3H), 1.06 (s, 3H).  ^13^C NMR (101 MHz, CDCl_3_) δ 144.64, 127.63, 126.89, 126.74, 78.57, 75.34, 25.29, 24.77, 24.32. HRMS (ESI) *m/z* calcd for ([C_11_H_16_O_2_]+Na)^+^ [M+Na]^+^: 203.1048, found 203.1040.

Colorless oil. ^1^H NMR (400 MHz, CDCl_3_) δ 7.35 (d, *J* = 8.8 Hz, 2H), 6.87 (d, *J* = 8.8 Hz, 2H), 3.81 (s, 3H), 2.16 (m, 2H), 1.96 (dd, *J* = 14.2, 7.2 Hz, 1H), 1.25 (m, 4H), 1.06 (s, 3H), 0.73 (t, *J* = 7.2 Hz, 3H).  ^13^C NMR (101 MHz, CDCl_3_) δ 158.32, 133.68, 128.43, 113.00, 81.04, 75.80, 55.19, 26.86, 25.65, 24.57, 7.96. HRMS (ESI) *m/z* calcd for ([C_13_H_20_O_3_]+Na)^+^ [M+Na]^+^: 247.1310, found 247.1307.

White solid. ^1^H NMR (400 MHz, CDCl_3_) δ 7.40 (d, *J* = 8.6 Hz, 2H), 7.34 (d, *J* = 8.6 Hz, 2H), 2.17 (s, 2H), 1.63 (s, 3H), 1.32 (s, 9H), 1.27 (s, 3H), 1.09 (s, 3H). ^13^C NMR (101 MHz, CDCl_3_) δ 149.70, 141.55, 126.41, 124.55, 78.47, 75.36, 34.38, 31.36, 25.35, 24.72, 24.36. HRMS (ESI) *m/z* calcd for ([C_15_H_24_O_2_]+Na)^+^ [M+Na]^+^: 259.1674, found 259.1675.

Yellow oil. ^1^H NMR (400 MHz, CDCl_3_) δ 7.48 (d, *J* = 7.6 Hz, 2H), 7.30 (t, *J* = 7.7 Hz, 2H), 7.22 (t, *J* = 7.3 Hz, 1H), 2.56 (dt, *J* = 13.4, 6.7 Hz, 1H), 2.27 (s, 2H), 1.45 (s, 3H), 1.20 (d, *J* = 6.7 Hz, 3H), 0.81 (s, 3H), 0.62 (d, *J* = 6.8 Hz, 3H). ^13^C NMR (101 MHz, CDCl_3_) δ 144.74, 127.73, 126.35, 125.93, 81.29, 76.40, 33.71, 28.63, 24.54, 19.14, 18.45. HRMS (ESI) *m/z* calcd for ([C_13_H_20_O_2_]+Na)^+^ [M+Na]^+^: 231.1361, found 231.1360.

White solid. ^1^H NMR (400 MHz, CDCl_3_) δ 7.51 (d, *J* = 7.6 Hz, 2H), 7.32 (t, *J* = 7.4 Hz, 2H), 7.24 (d, *J* = 7.8 Hz, 1H), 7.14 – 7.05 (m, 3H), 6.92 (dd, *J* = 6.5, 2.9 Hz, 2H), 3.49 (dd, *J* = 57.1, 13.6 Hz, 2H), 2.16 (s, 2H), 1.36 (s, 3H), 1.04 (s, 3H). ^13^C NMR (101 MHz, CDCl_3_) δ 142.70, 136.31, 130.76, 128.36, 127.65, 127.29, 126.95, 126.83, 80.67, 75.36, 40.47, 25.72, 23.98. HRMS (ESI) *m/z* calcd for ([C_17_H_20_O_2_]+Na)^+^ [M+Na]^+^: 279.1361, found 231.1353.

Light yellow oil. ^1^H NMR (400 MHz, CDCl_3_) δ 7.53 – 7.17 (m, 5H), 3.74 (dd, *J* = 59.3, 11.2 Hz, 2H), 2.26 (s, 2H), 1.92 – 1.71 (m, 2H), 0.76 (t, *J* = 7.5 Hz, 3H). ^13^C NMR (101 MHz, CDCl_3_) δ 143.21, 128.37, 127.01, 125.64, 77.52, 70.37, 31.14, 7.41.

Light yellow oil. ^1^H NMR (400 MHz, CDCl_3_) δ 7.44 (d, *J* = 7.1 Hz, 1H), 7.36 (dd, *J* = 14.2, 5.8 Hz, 3H), 7.25 (dd, *J* = 12.4, 8.2 Hz, 1H), 3.98 (dq, *J* = 39.4, 6.4 Hz, 1H), 2.22 – 2.07 (s, 1H), 2.06 – 1.88 (m, 2H), 1.84 (q, *J* = 7.4 Hz, 1H), 1.21 (d, *J* = 6.4 Hz, 1.5H), 0.93 (d, *J* = 6.4 Hz, 1.5H), 0.72 (dt, *J* = 9.0, 7.4 Hz, 3H). ^13^C NMR (101 MHz, CDCl_3_) δ 143.58, 142.24, 128.34, 128.06, 127.00, 126.66, 126.15, 125.85, 79.46, 79.20, 73.99, 31.25, 28.84, 17.91, 16.21, 7.65, 7.58. HRMS (ESI) *m/z* calcd for ([C_11_H_16_O_2_]+Na)^+^ [M+Na]^+^: 203.1048, found 203.1045.

Colorless oil. ^1^H NMR (400 MHz, DMSO) δ 7.35 (d, *J* = 8.8 Hz, 2H), 6.84 (d, *J* = 8.8 Hz, 2H), 4.68 (m, 2H), 3.72 (s, 3H), 3.35 (s, 2H), 1.36 (s, 3H).  ^13^C NMR (101 MHz, DMSO) δ 158.10, 139.92, 127.03, 113.34, 73.76, 71.04, 55.42, 26.56. HRMS (ESI) *m/z* calcd for ([C_10_H_14_O_3_]+Na)^+^ [M+Na]^+^: 205.0841, found 205.0837.

Colorless oil. ^1^H NMR (400 MHz, DMSO) δ 7.33 (dd, *J* = 14.1, 8.8 Hz, 2H), 6.83 (d, *J* = 8.8 Hz, 2H), 4.68 (s, 0.5H), 4.57 (s, 0.5H), 4.46 (d, *J* = 4.7 Hz, 0.5H), 4.37 (d, *J* = 6.0 Hz, 0.5H), 3.72 (s, 3H), 3.64 – 3.49 (m, 1H), 1.39 (d, *J* = 1.7 Hz, 2H), 0.81 (m, 2H).  ^13^C NMR (101 MHz, DMSO) δ 157.99, 157.86, 140.11, 138.98, 127.76, 127.09, 113.19, 113.00, 75.61, 75.44, 73.96, 73.40, 55.36, 26.42, 25.57, 18.30, 18.14. HRMS (ESI) *m/z* calcd for ([C_11_H_16_O_3_]+Na)^+^ [M+Na]^+^: 219.0997, found 219.0997.

Colorless oil. ^1^H NMR (400 MHz, CDCl_3_) δ 7.41 (d, *J* = 8.8 Hz, 2H), 6.86 (d, *J* = 8.8 Hz, 2H), 3.81 (s, 3H), 2.51 (s, 1H), 1.93 (s, 1H), 1.62 (s, 3H), 1.26 (s, 3H), 1.08 (s, 3H). ^13^C NMR (101 MHz, CDCl_3_) δ 158.48, 136.73, 127.86, 112.95, 78.30, 75.42, 55.24, 25.28, 24.78, 24.44. HRMS (ESI) *m/z* calcd for ([C_12_H_18_O_3_]+Na)^+^ [M+Na]^+^: 233.1154, found 233.1152.

Light yellow oil. ^1^H NMR (400 MHz, DMSO) δ 7.33 (dd, *J* = 15.0, 8.9 Hz, 2H), 6.82 (dd, *J* = 8.9, 2.9 Hz, 2H), 4.69 (s, 0.4H), 4.58 (s, 0.6H), 4.52 (d, *J* = 5.4 Hz, 0.4H), 4.29 (d, *J* = 7.0 Hz, 0.6H), 3.72 (s, 3H), 3.24 (m, 1H), 1.39 (d, *J* = 5.4 Hz, 3H), 0.77 (t, *J* = 7.0 Hz, 3H). ^13^C NMR (101 MHz, DMSO) δ 157.98, 157.83, 140.31, 139.15, 127.73, 127.08, 113.20, 112.99, 80.09, 79.41, 75.67, 75.55, 55.36, 26.72, 25.80, 24.33, 24.05, 11.79. HRMS (ESI) *m/z* calcd for ([C_12_H_18_O_3_]+Na)^+^ [M+Na]^+^: 233.1154, found 233.1154.

White powder. ^1^H NMR (400 MHz, CDCl_3_) δ 7.12 (dd, *J* = 14.8, 8.8 Hz, 4H), 6.77 (dd, *J* = 8.8, 5.0 Hz, 4H), 3.80 (d, *J* = 5.7 Hz, 6H), 2.28 (m, 2H), 1.51 (d, *J* = 34.9 Hz, 6H). ^13^C NMR (101 MHz, CDCl_3_) δ 158.55, 158.46, 136.04, 135.71, 128.56, 128.12, 112.57, 112.42, 78.73, 78.50, 55.22, 25.19, 25.03. HRMS (ESI) *m/z* calcd for ([C_18_H_22_O_4_]+Na)^+^ [M+Na]^+^: 325.1416, found 325.1415.

Yellow oil. ^1^H NMR (400 MHz, CDCl_3_) δ 7.05 (d, *J* = 1.8 Hz, 1H), 6.85 (dt, *J* = 8.3, 5.1 Hz, 2H), 3.89 (s, 3H), 2.25 – 1.87 (m, 4H), 1.26 (s, 4H), 1.07 (s, 3H), 0.74 (t, *J* = 7.3 Hz, 3H). ^13^C NMR (101 MHz, CDCl_3_) δ 146.01, 144.32, 133.70, 120.14, 113.56, 110.47, 81.15, 75.88, 55.98, 26.97, 25.66, 24.63, 7.96.

**NMR spectra of products**


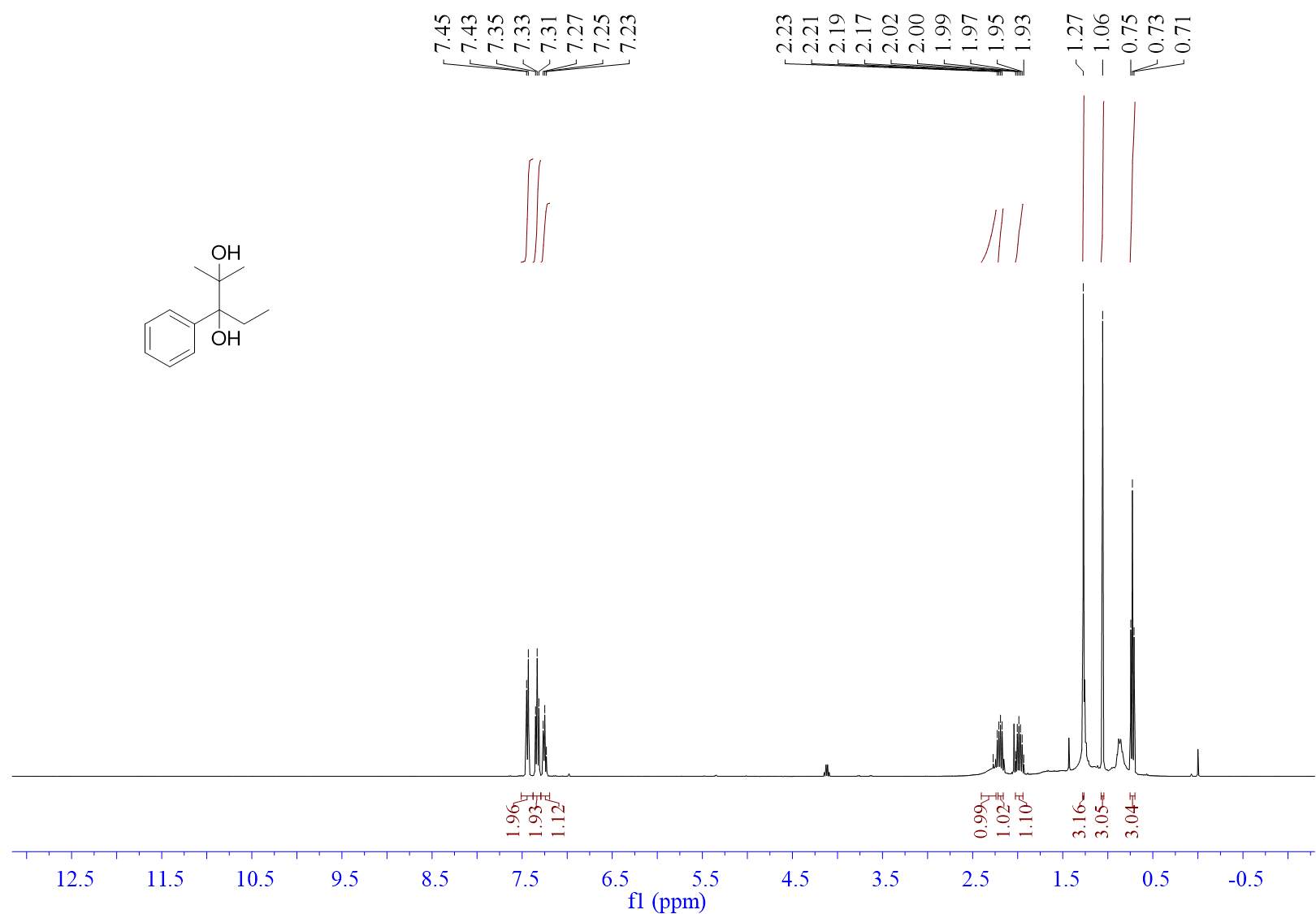


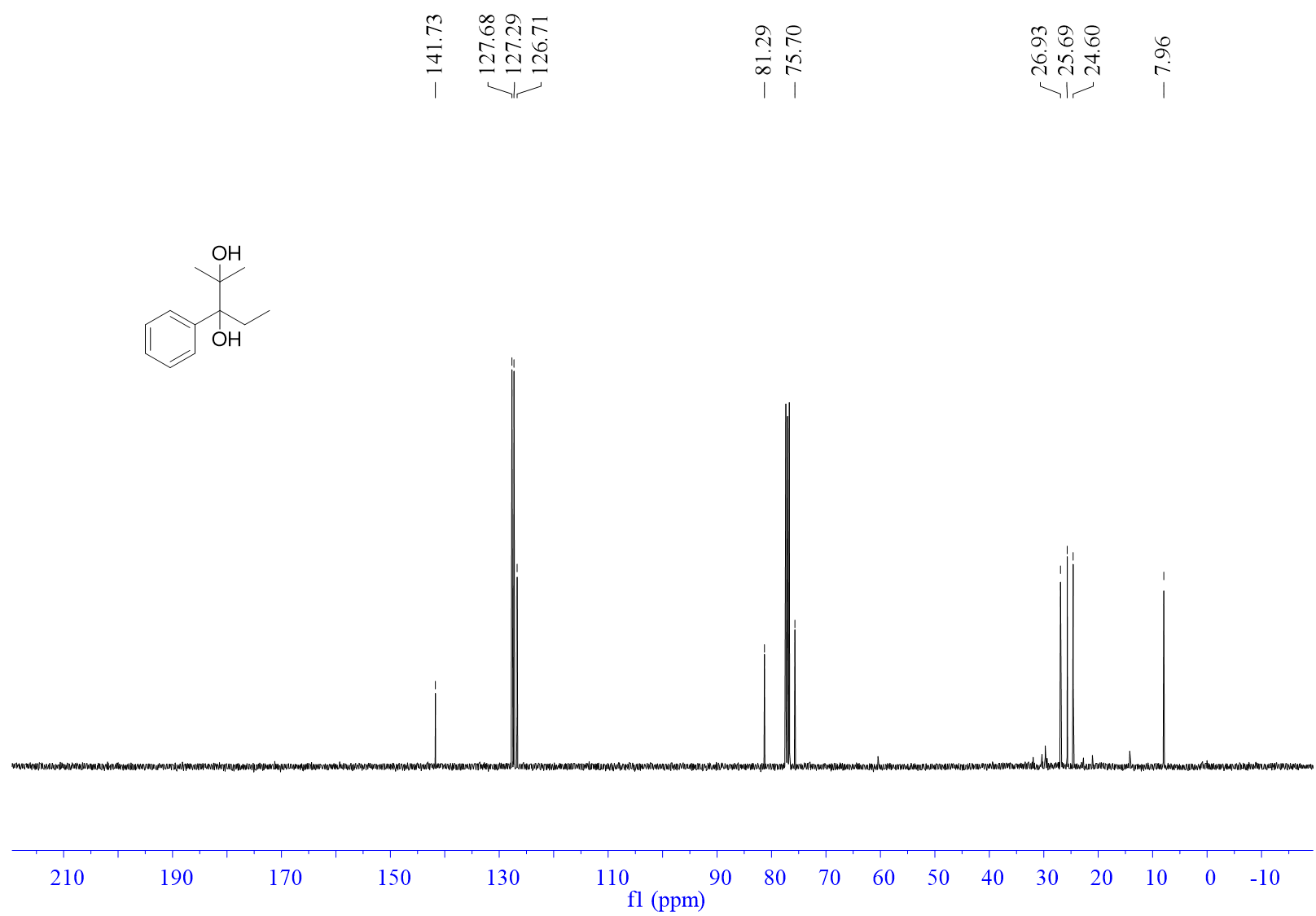


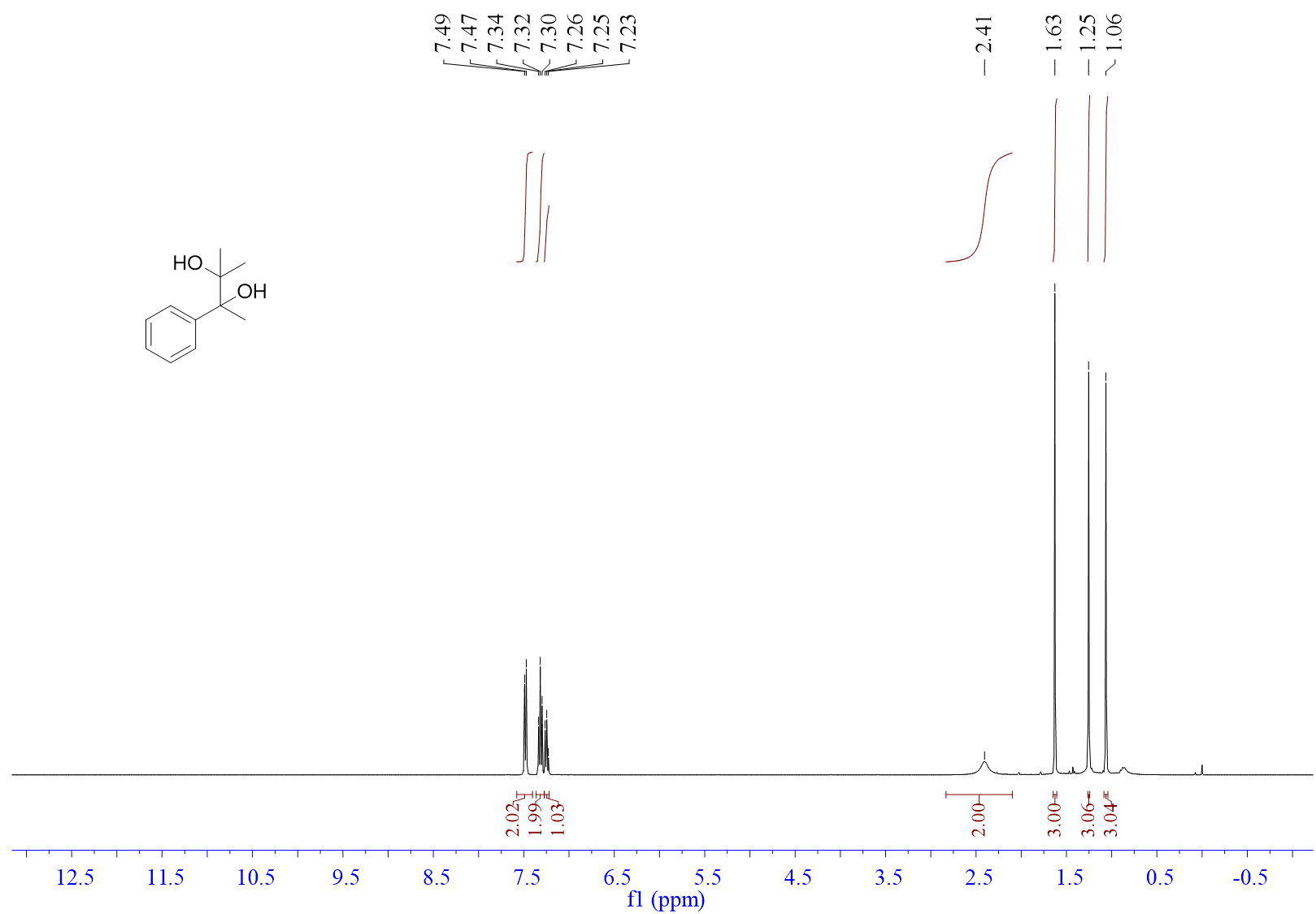


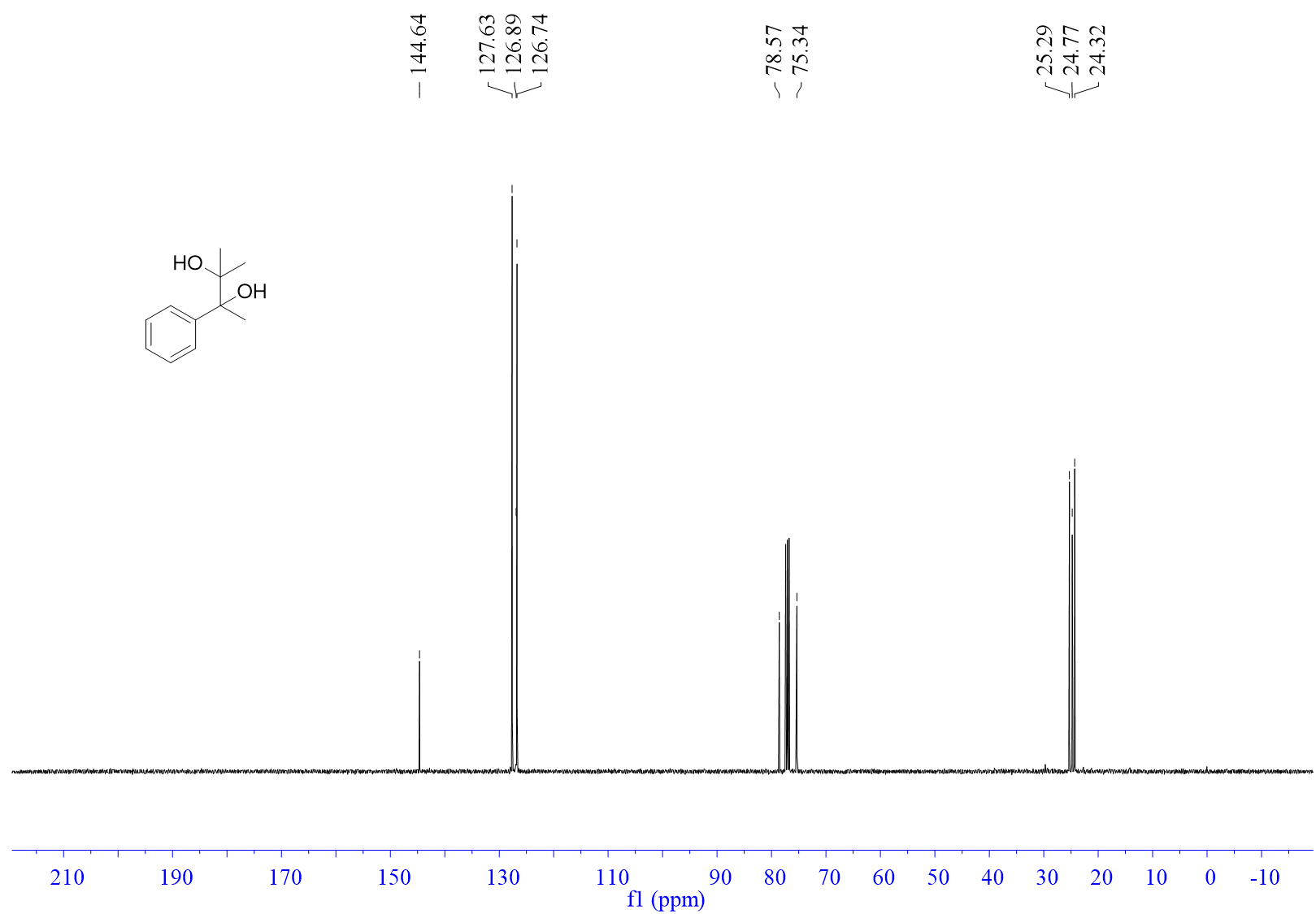


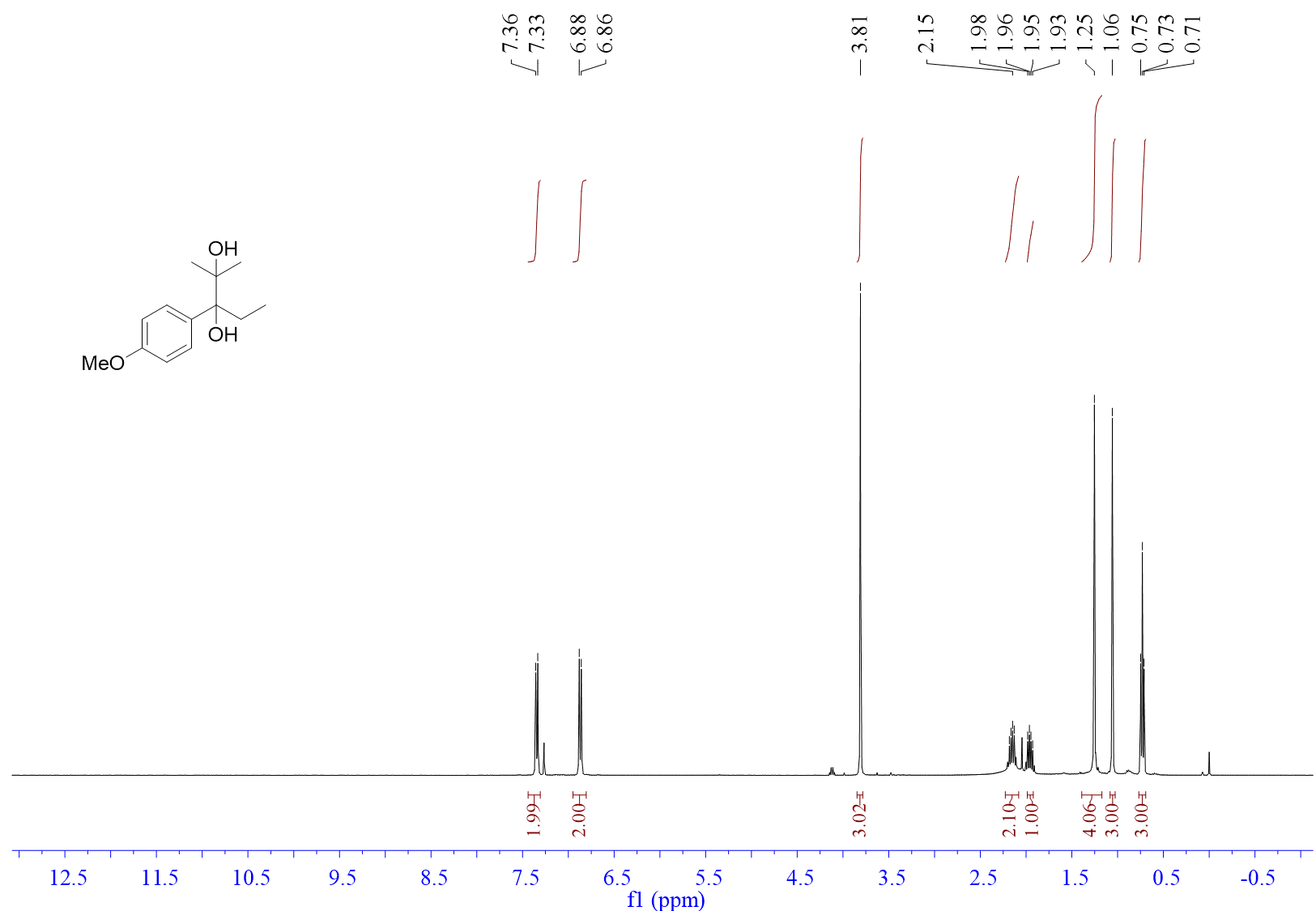


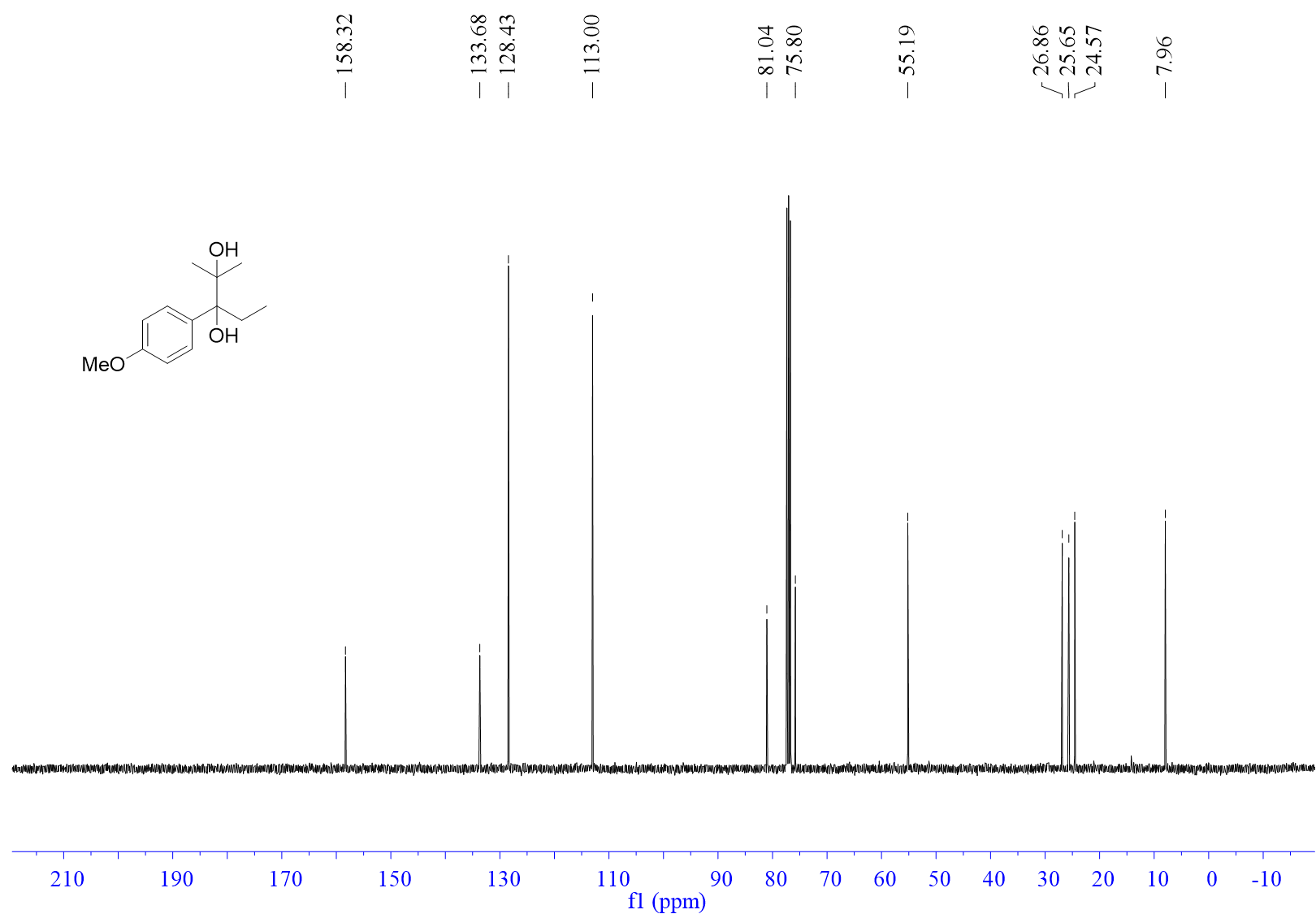


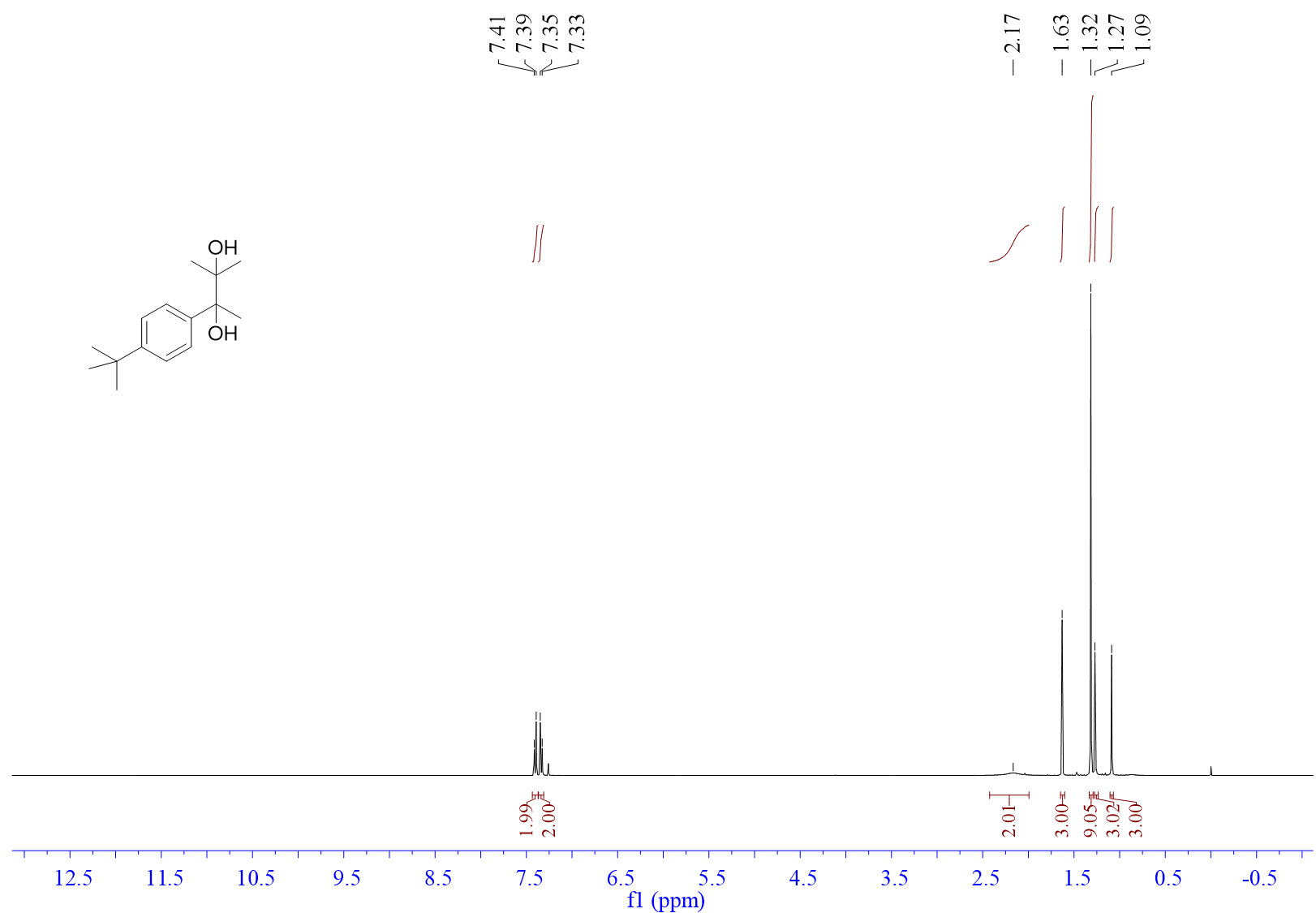


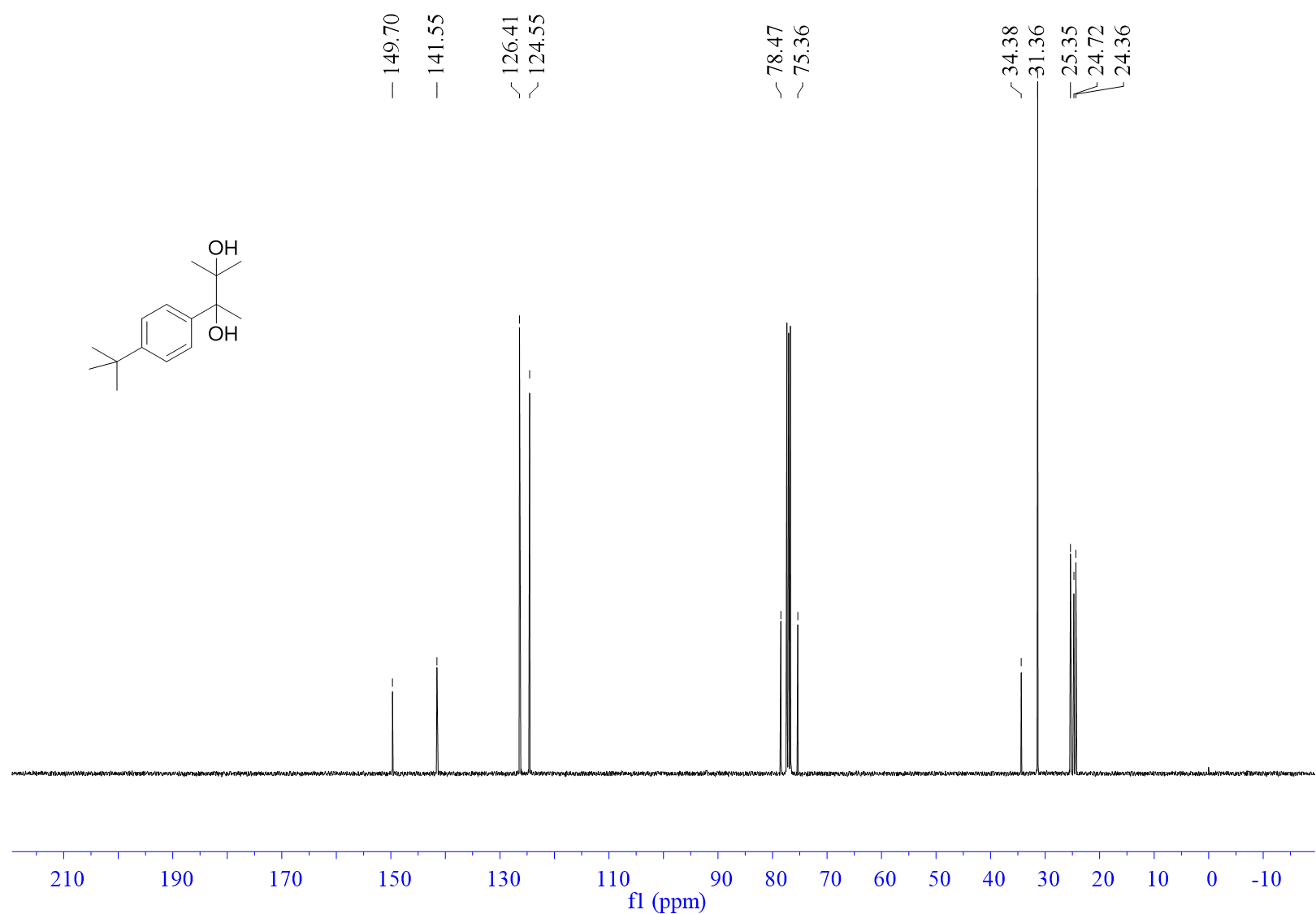


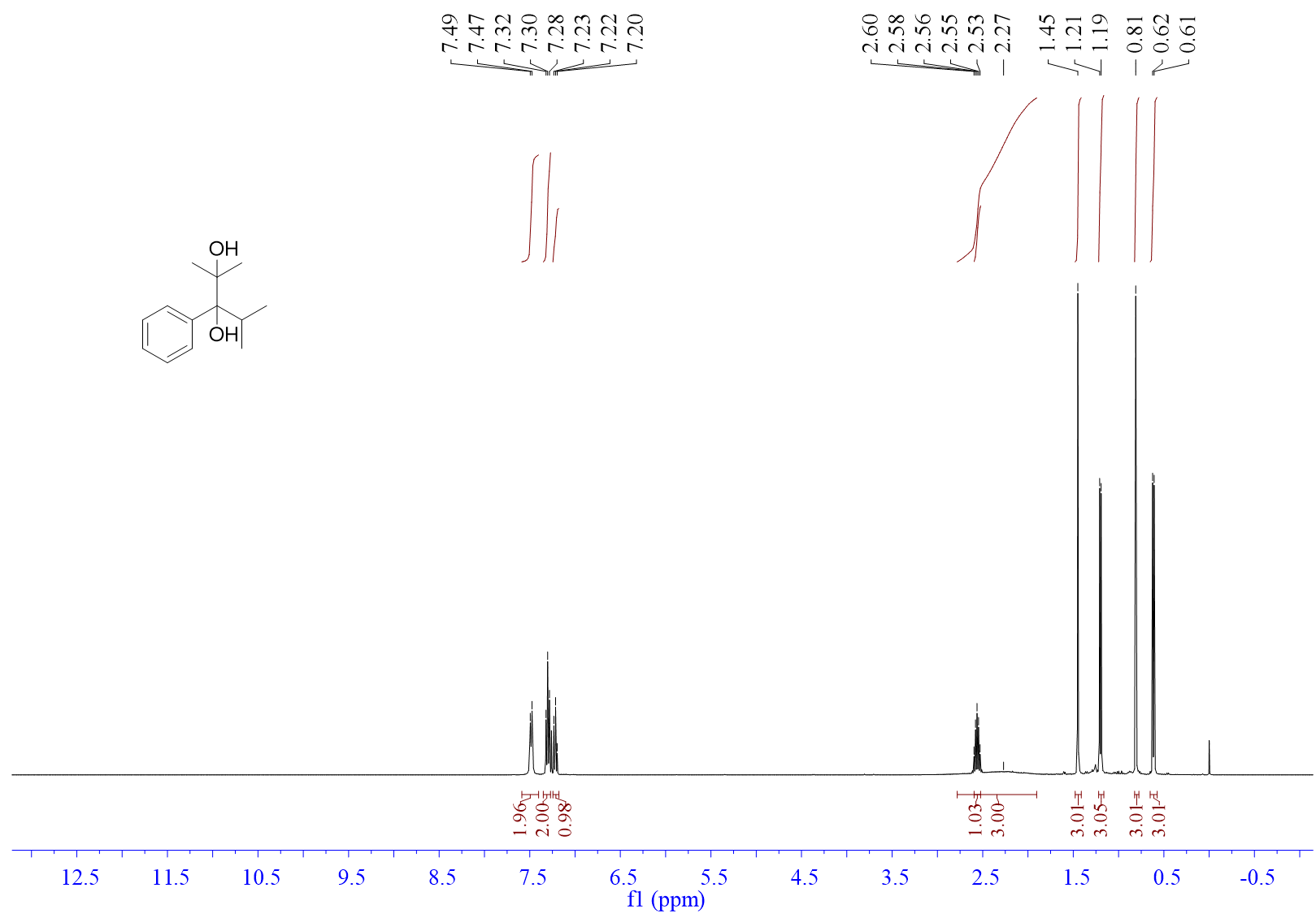


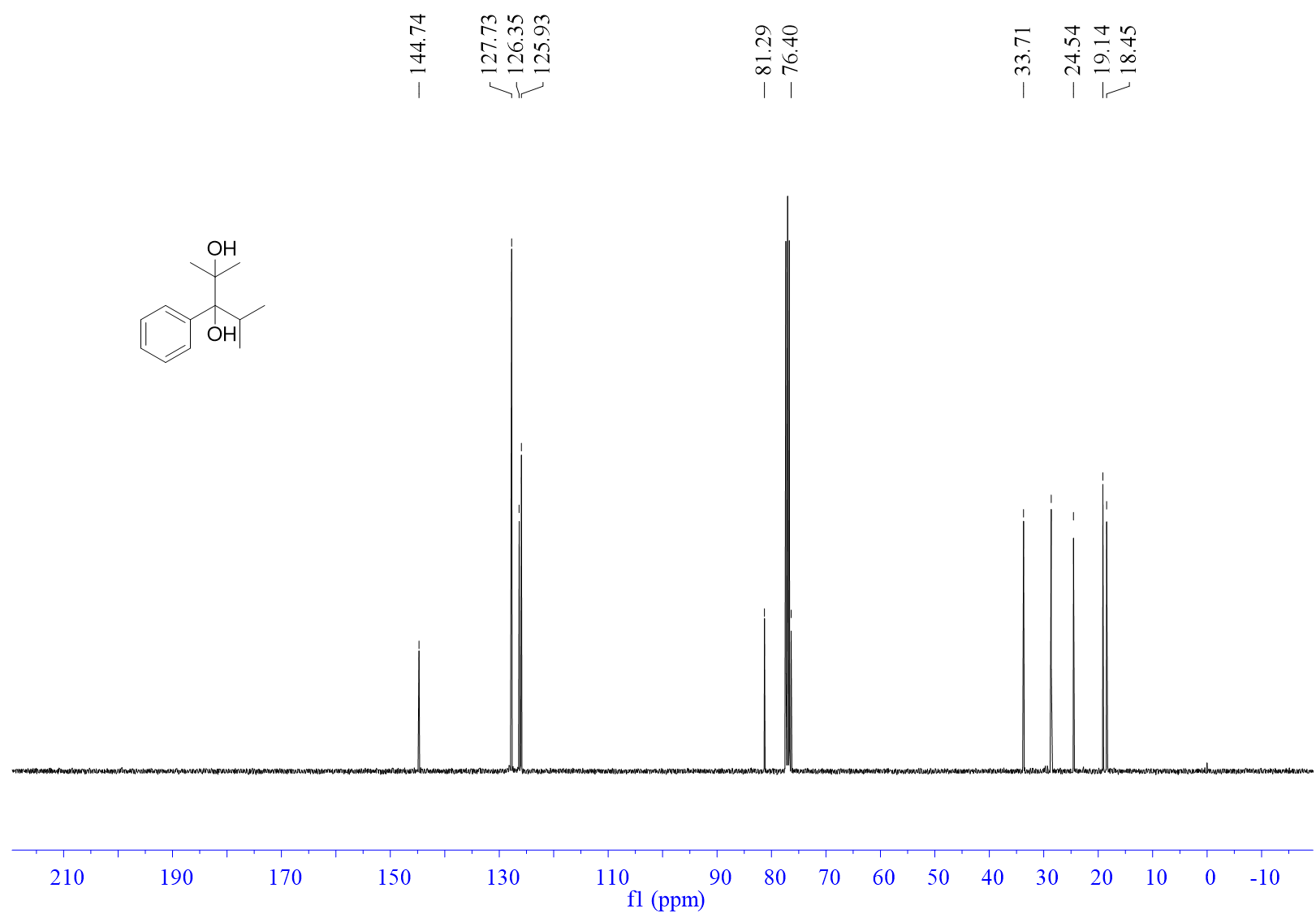


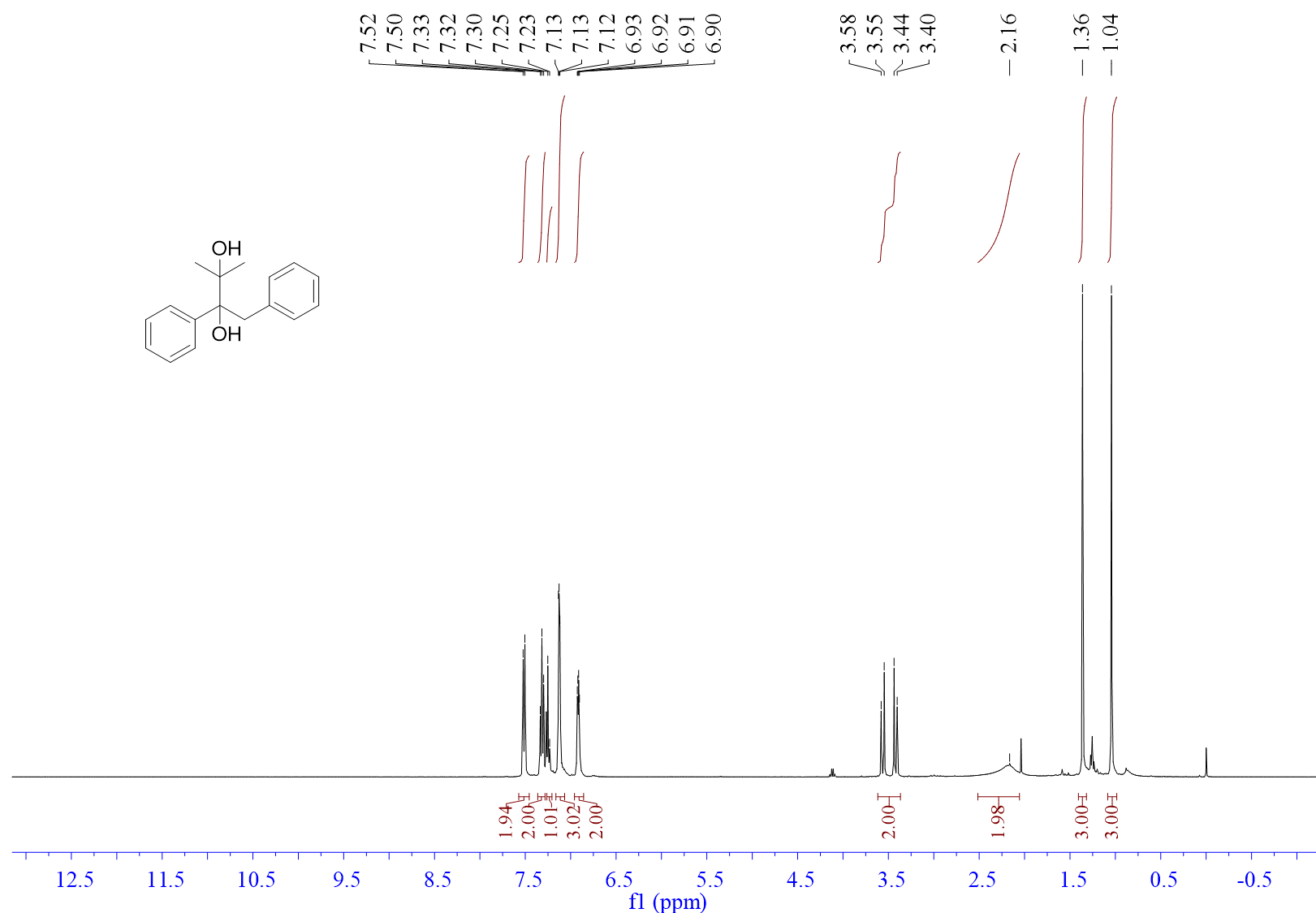


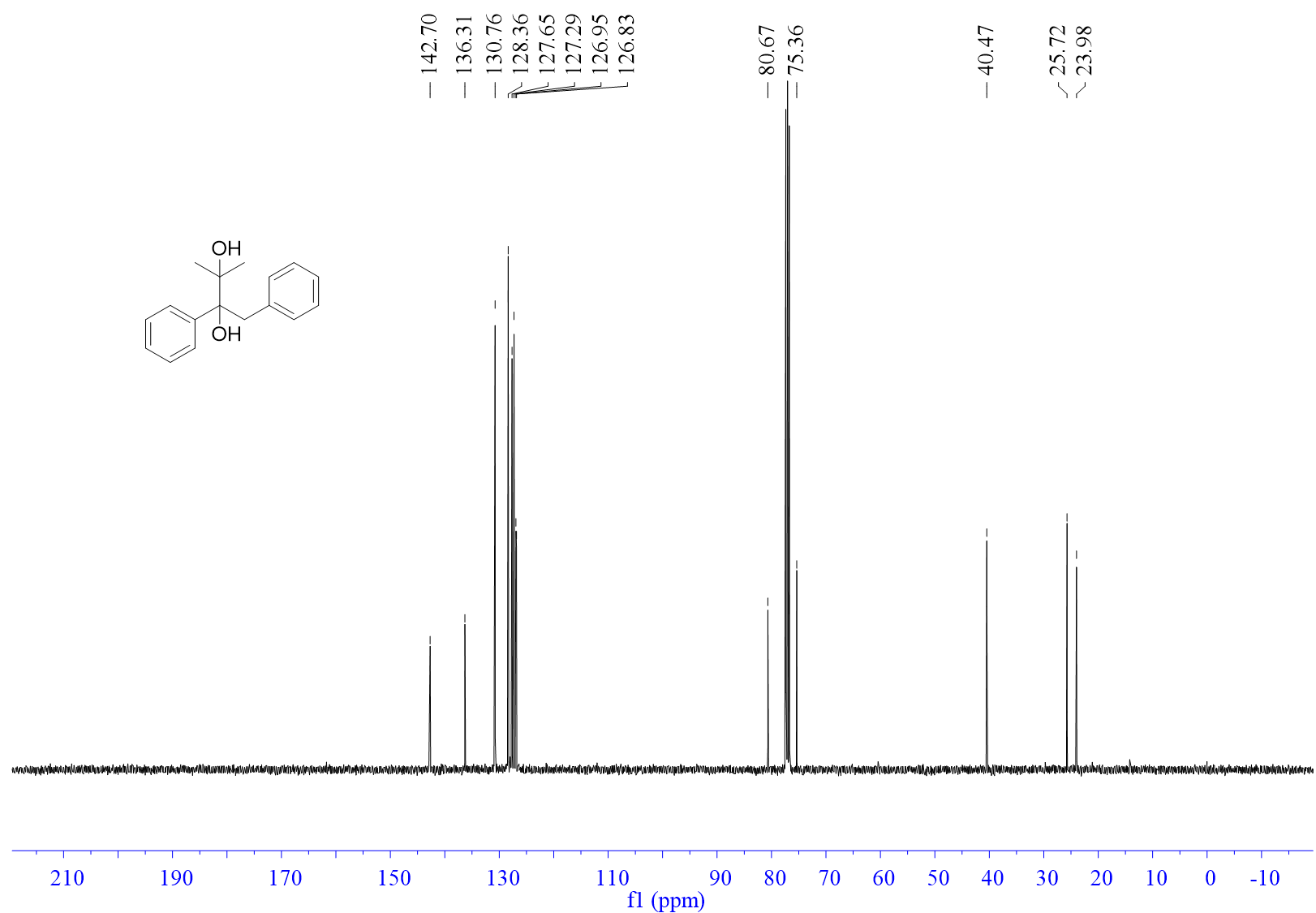


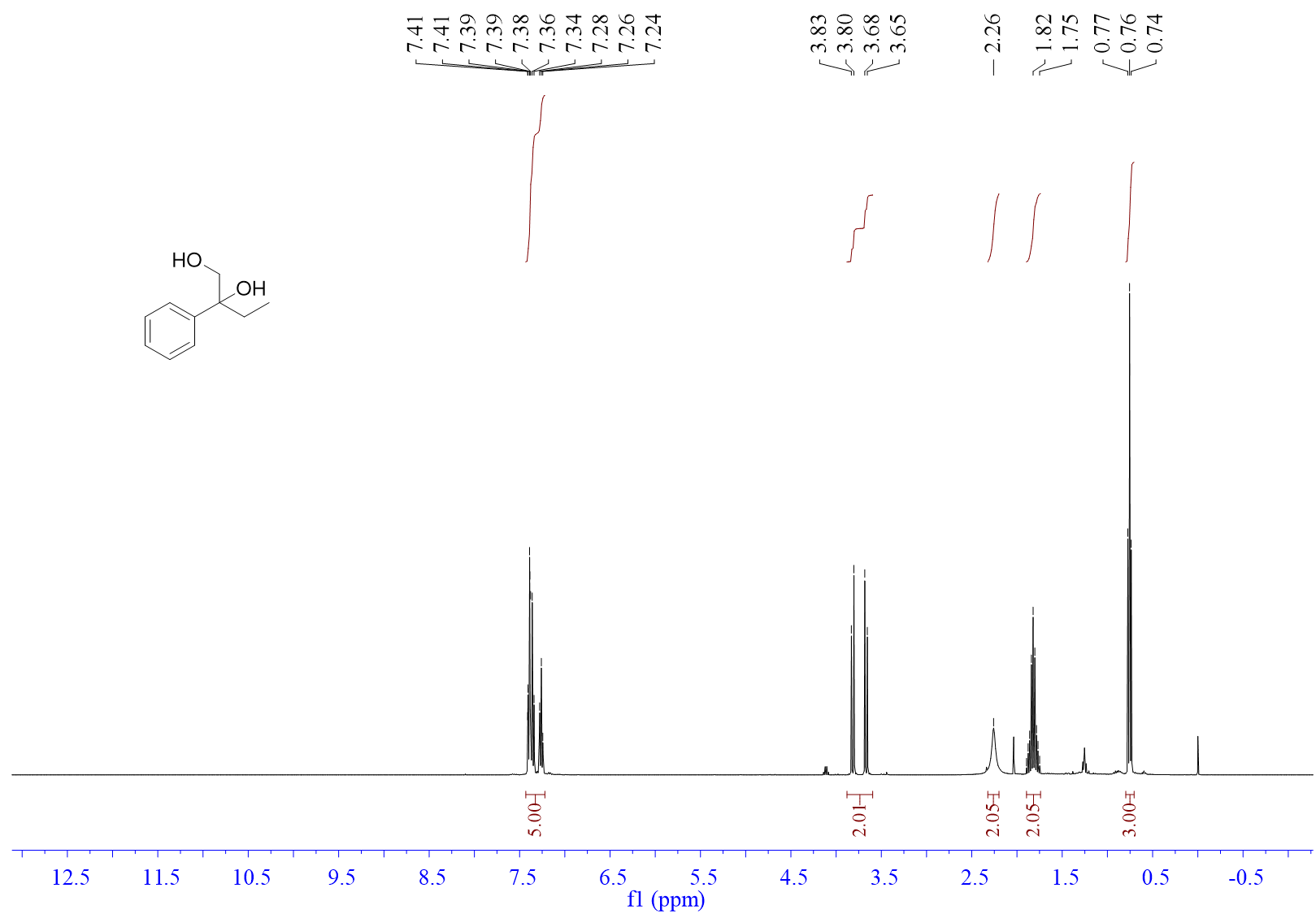


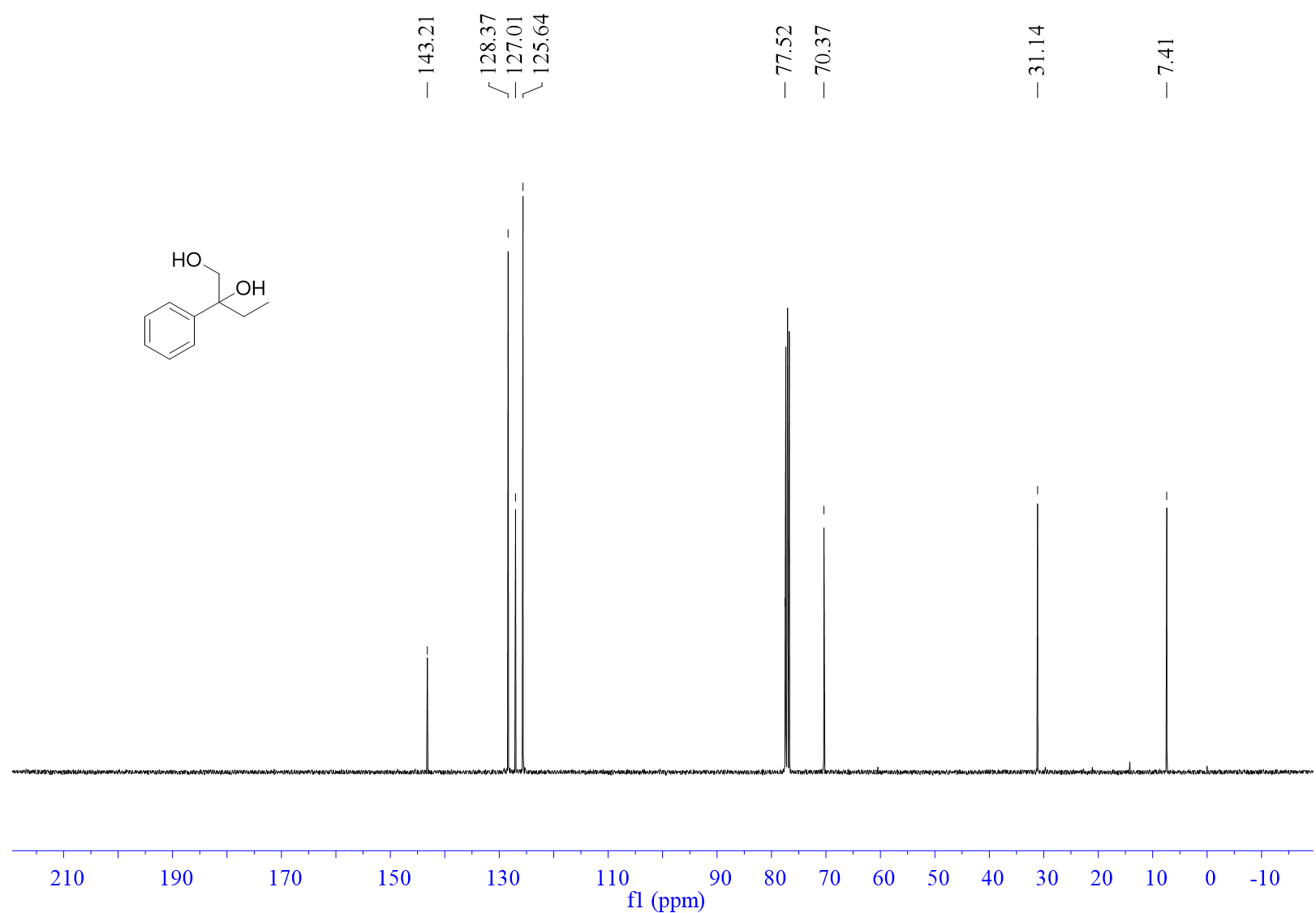


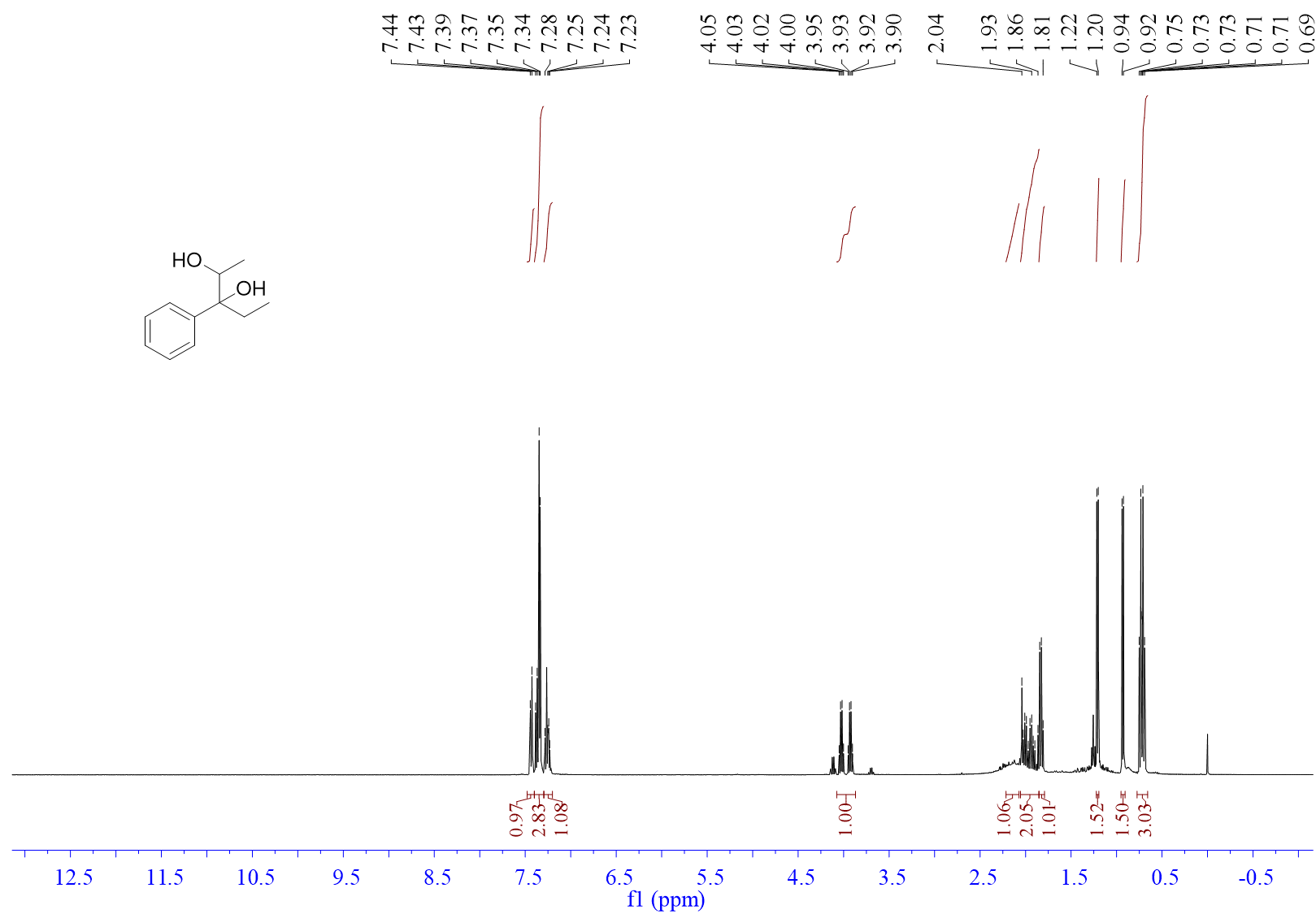

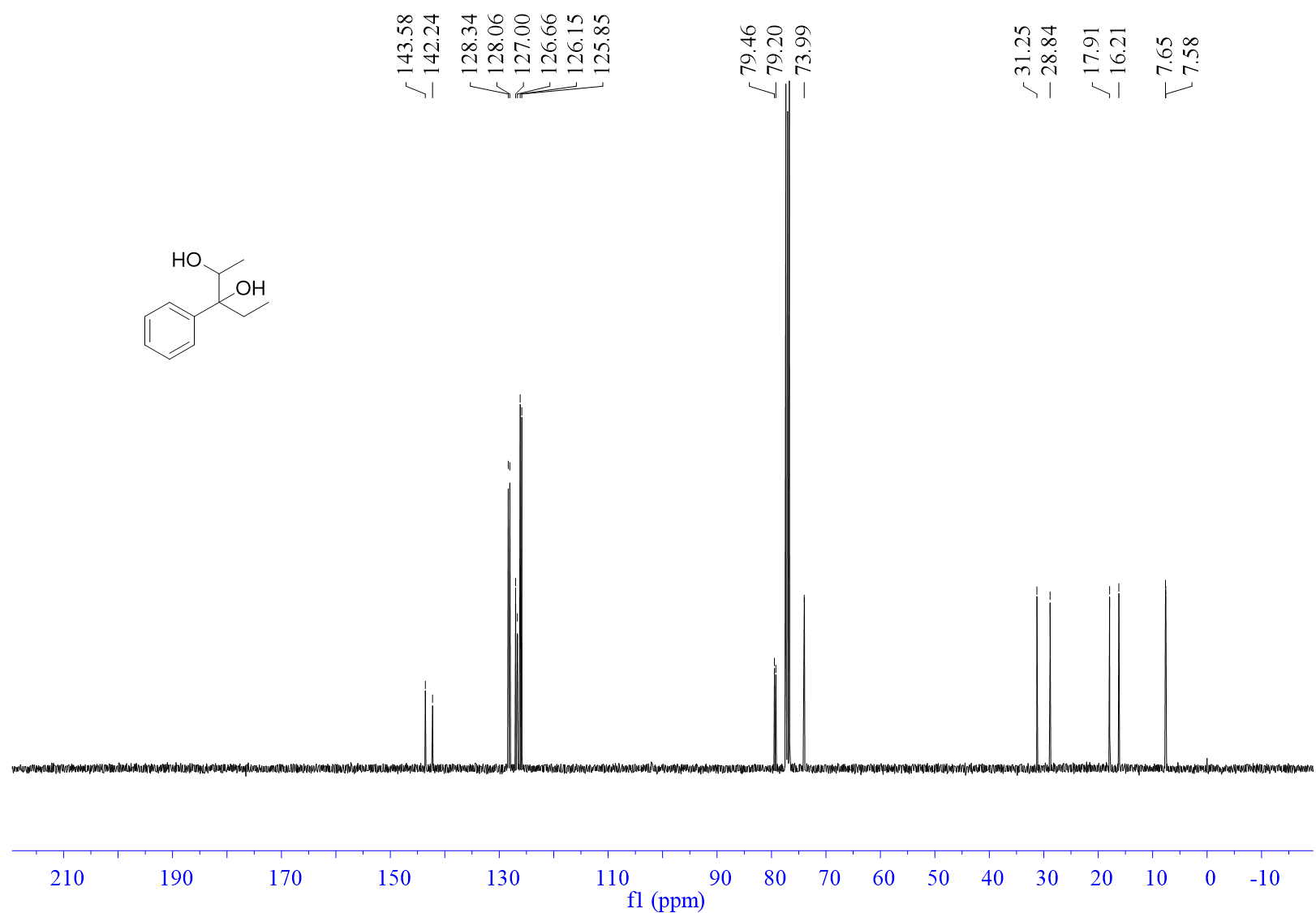


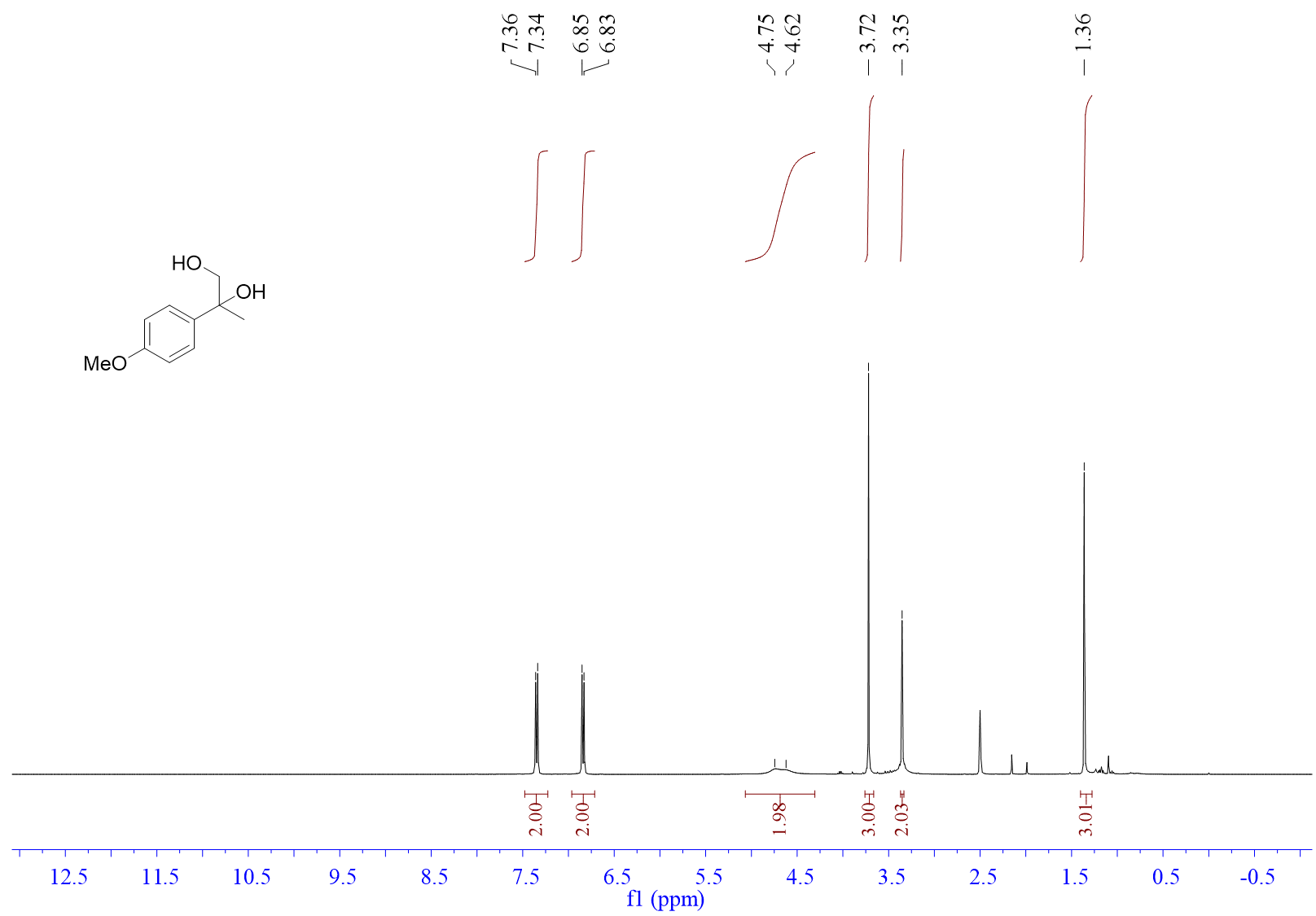


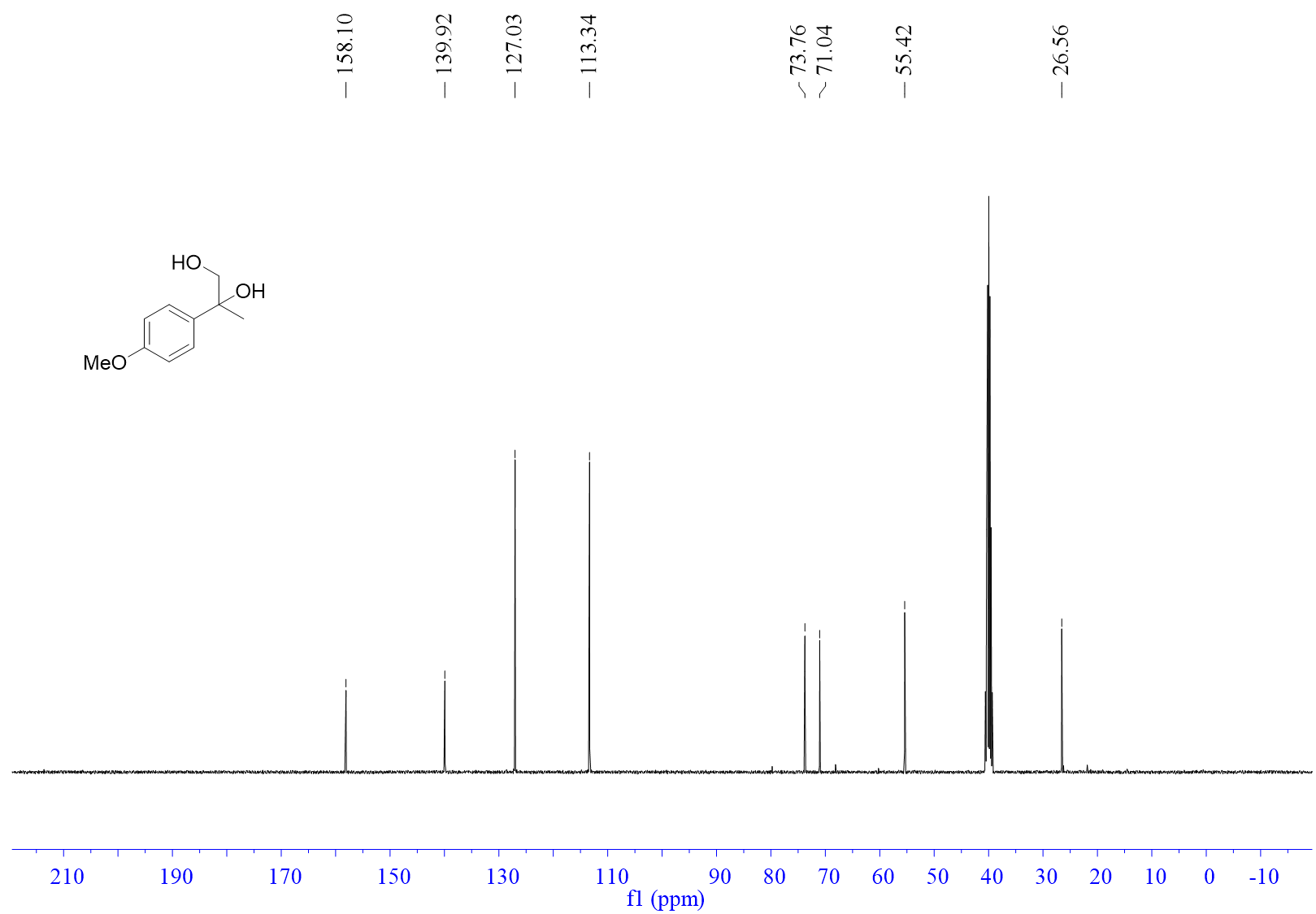


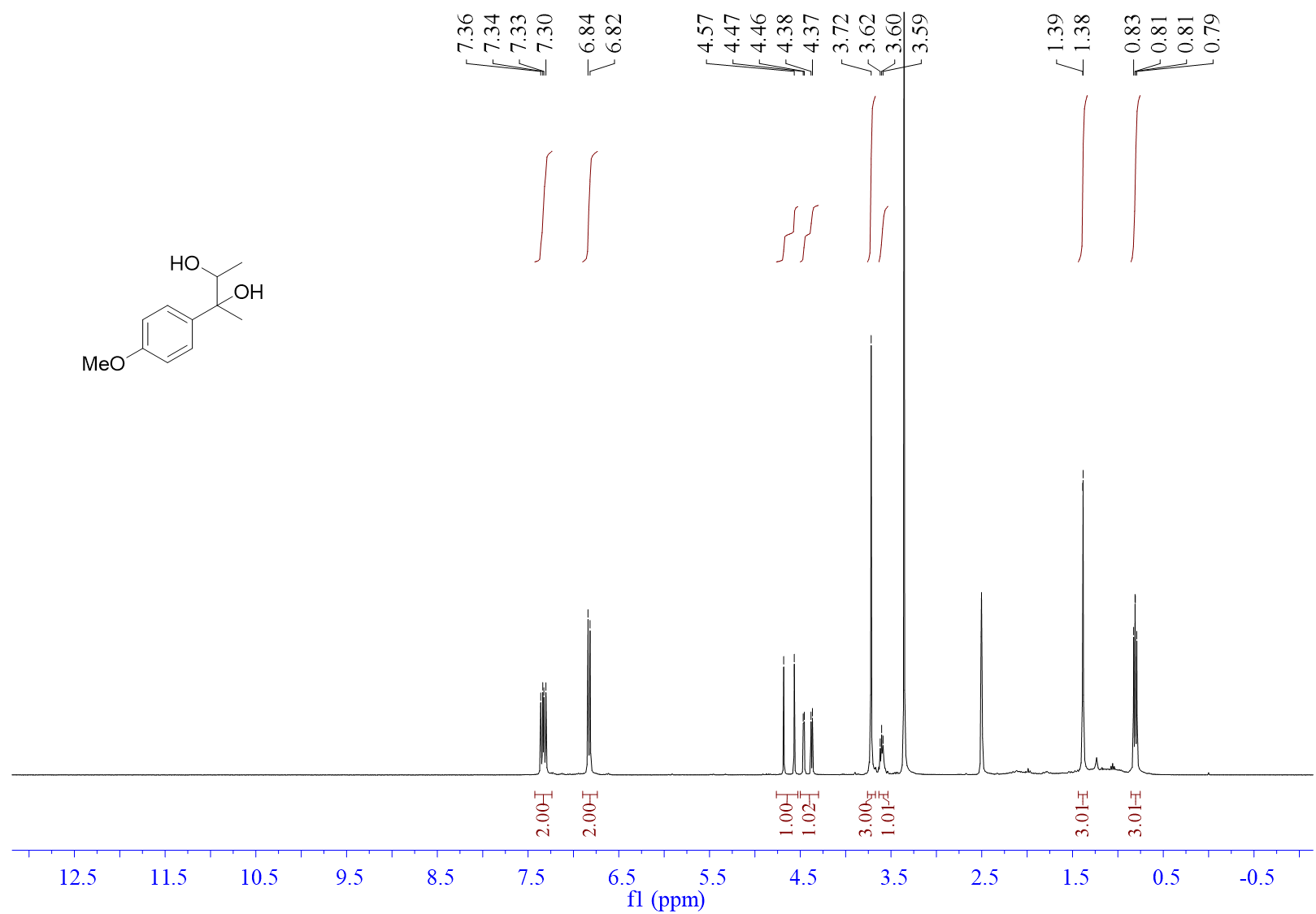


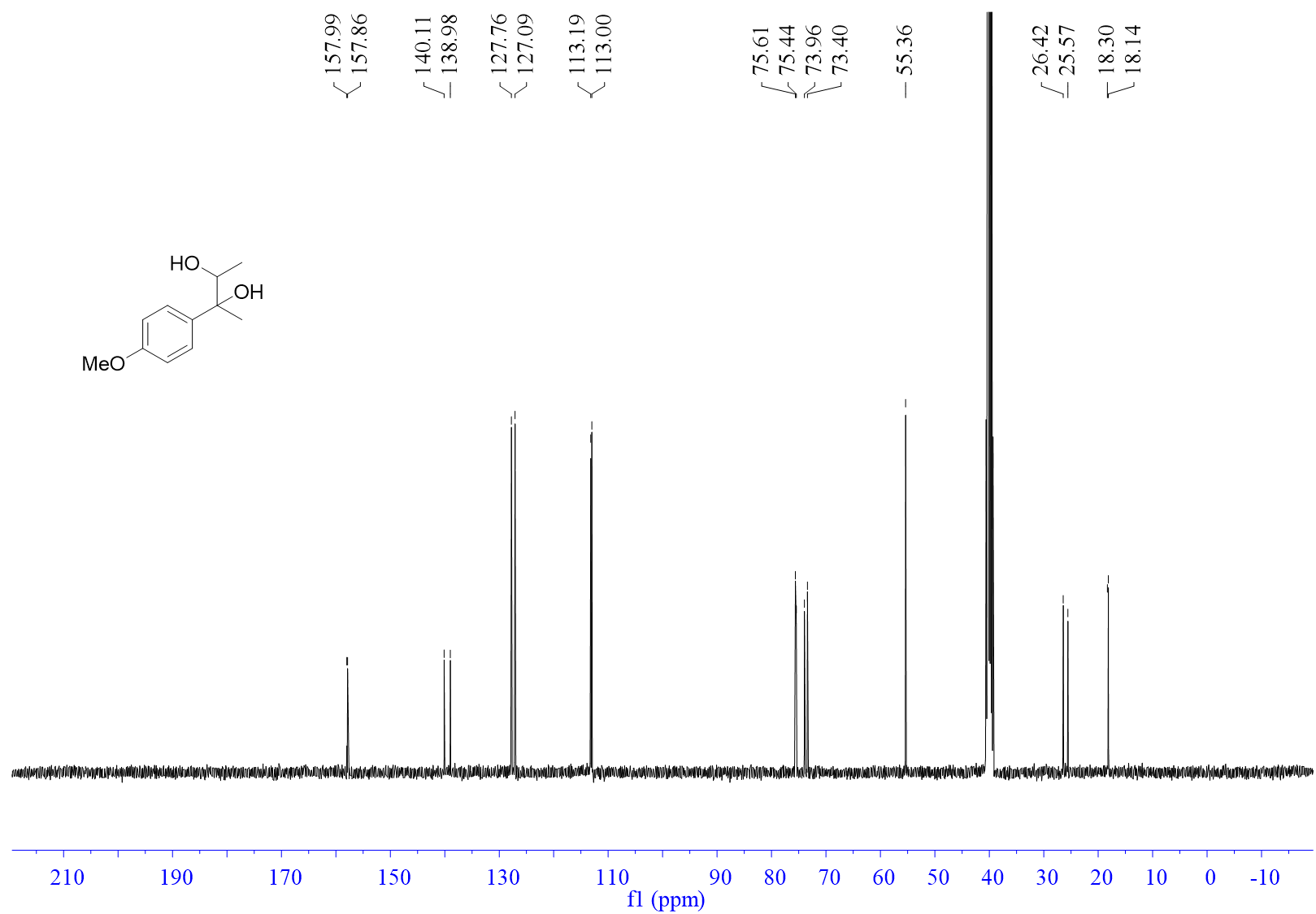


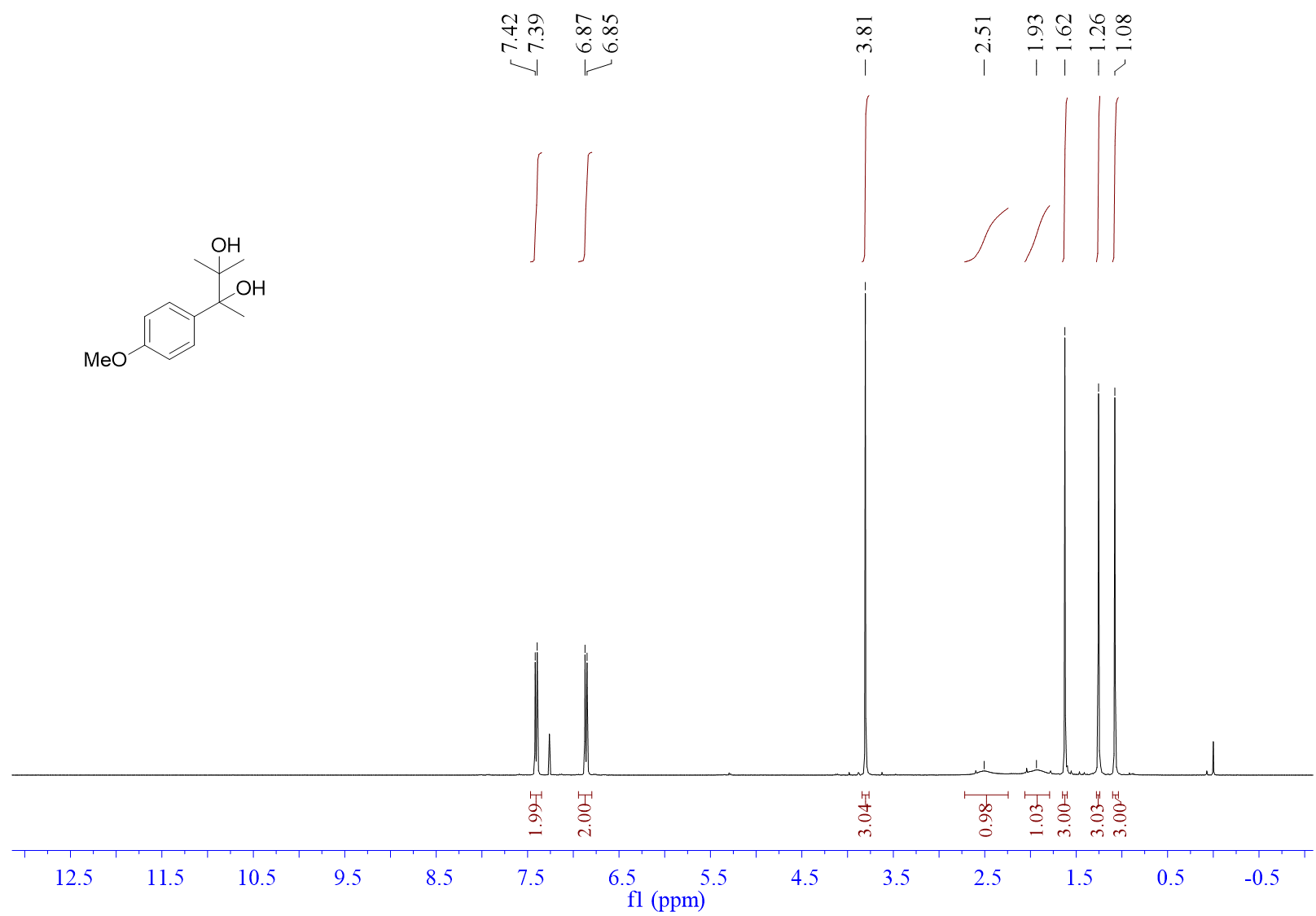


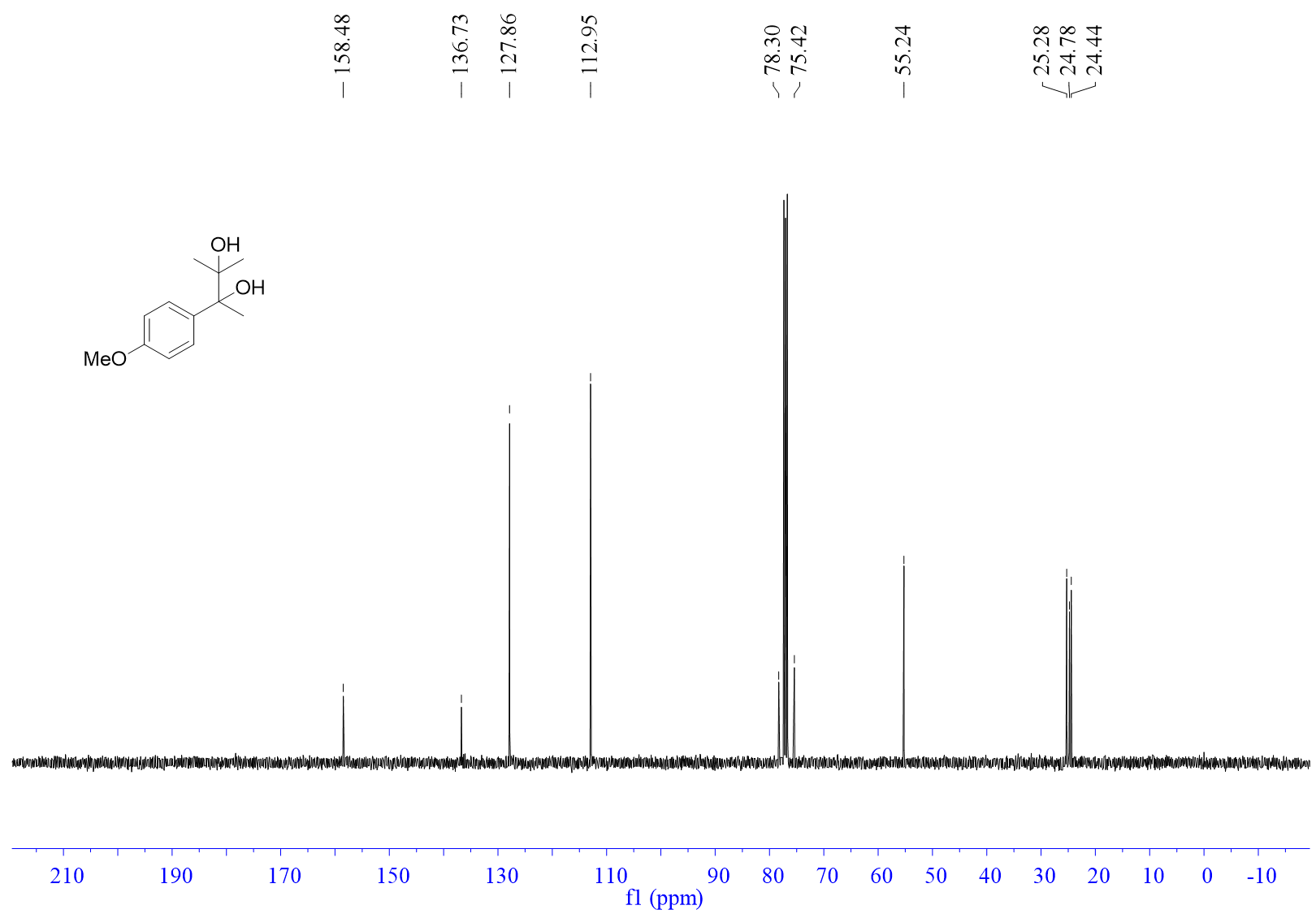


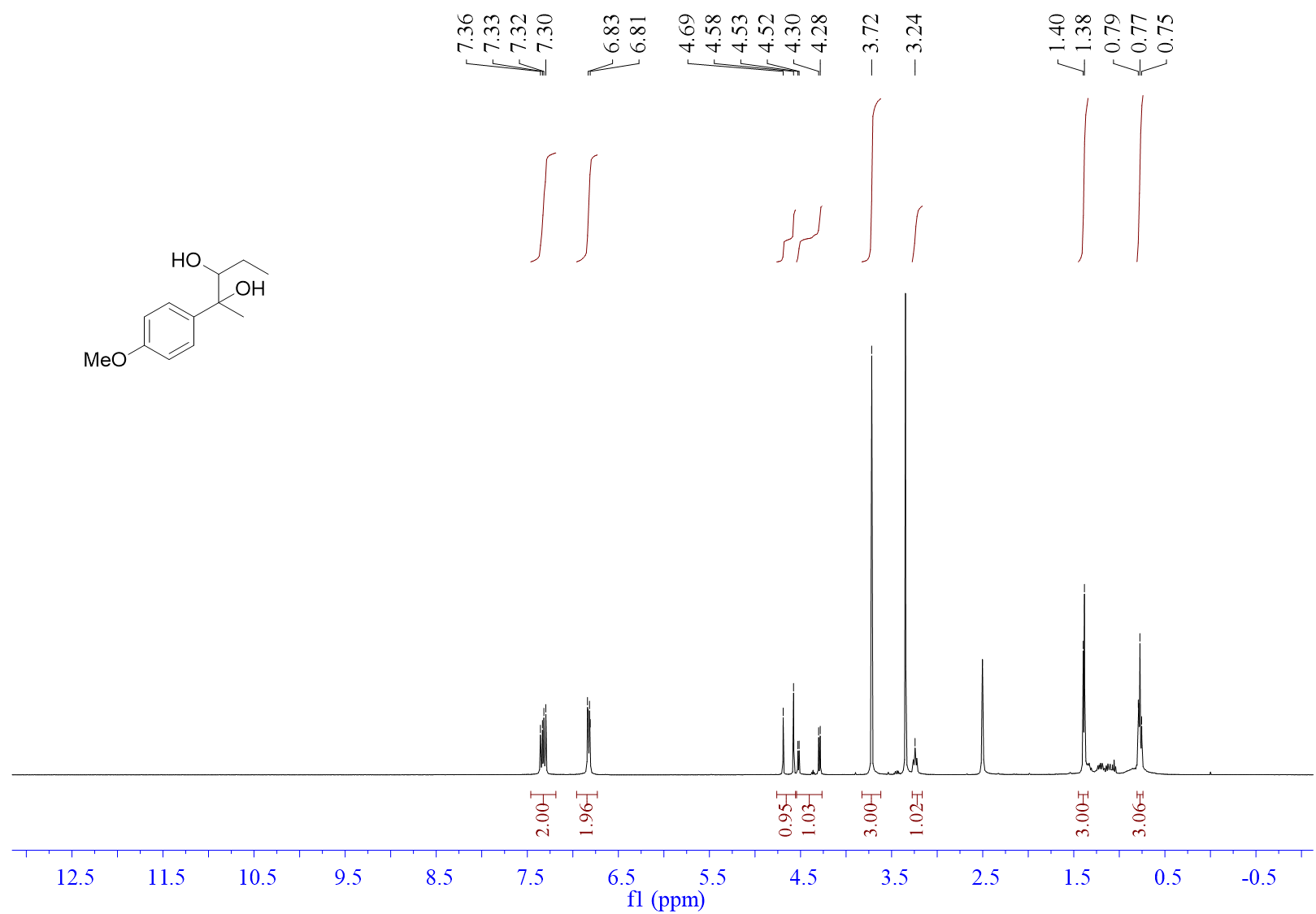


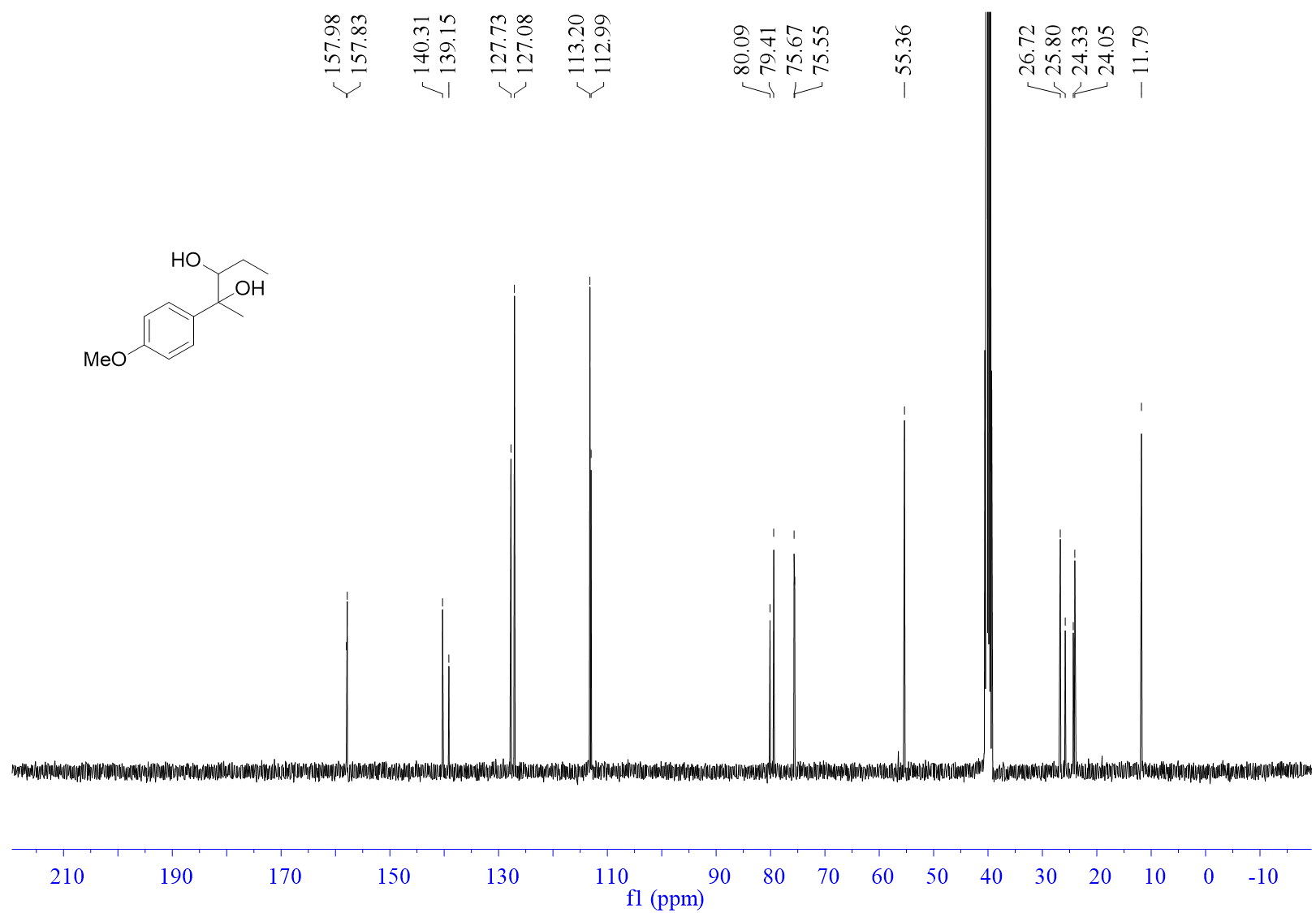


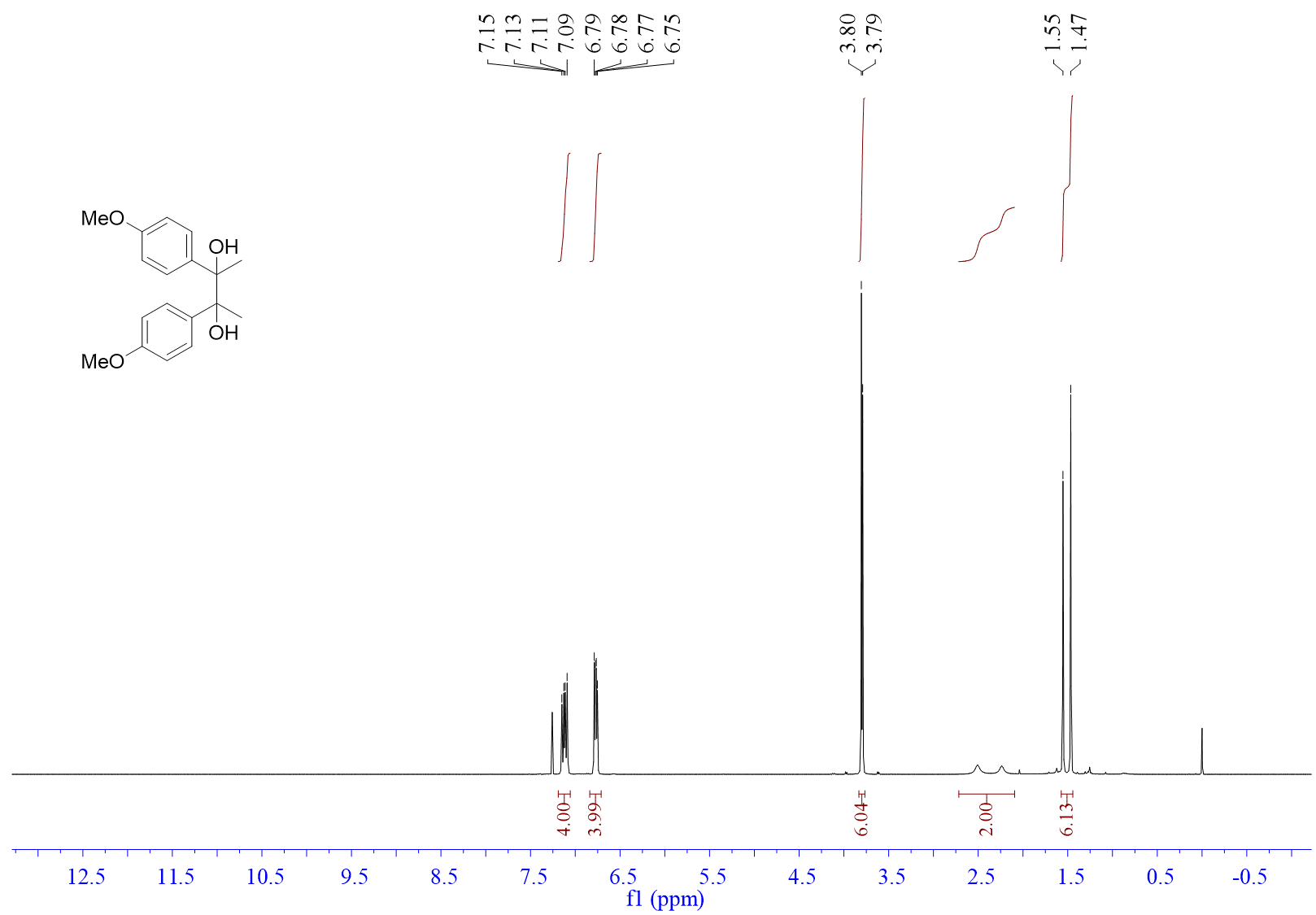


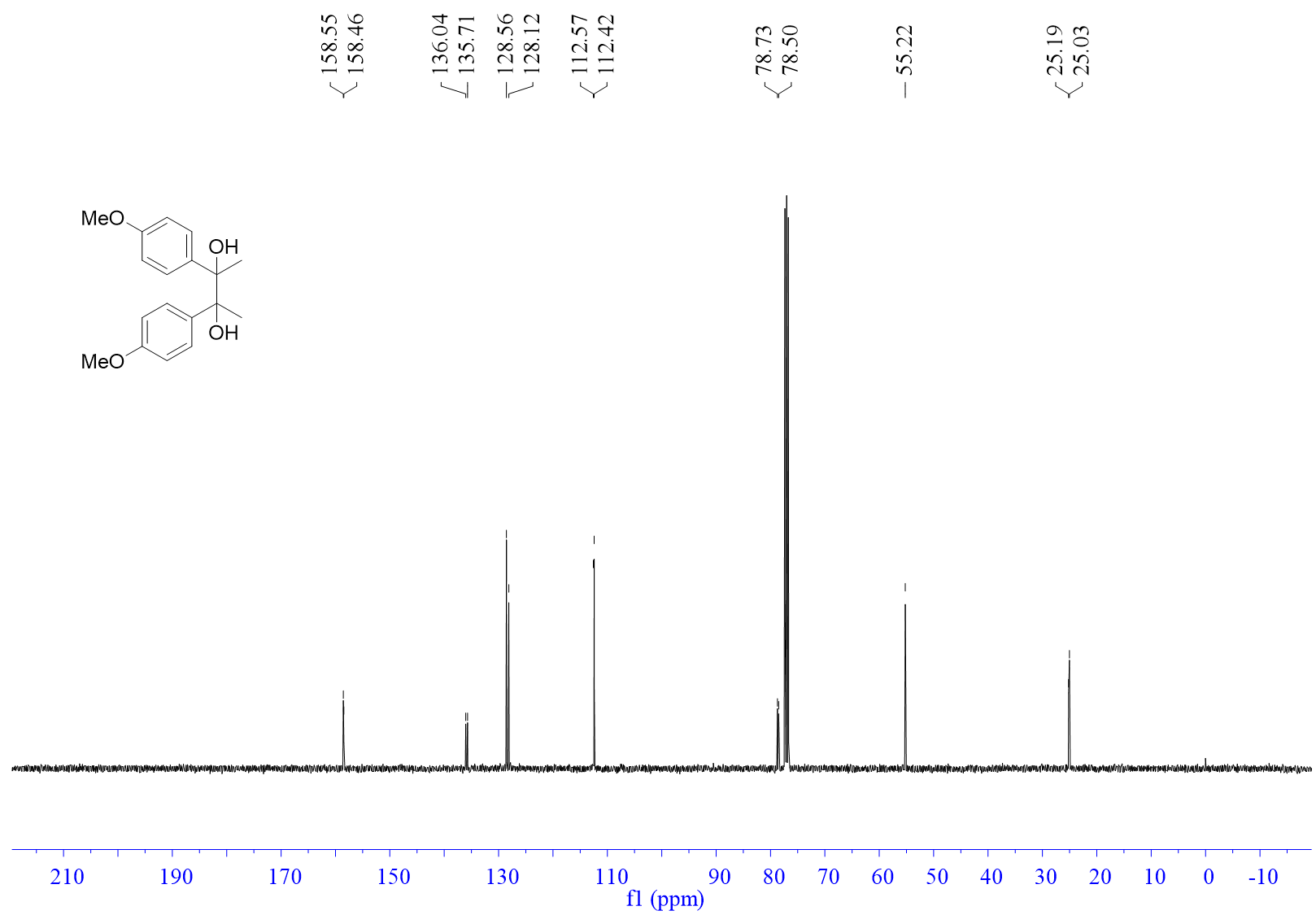


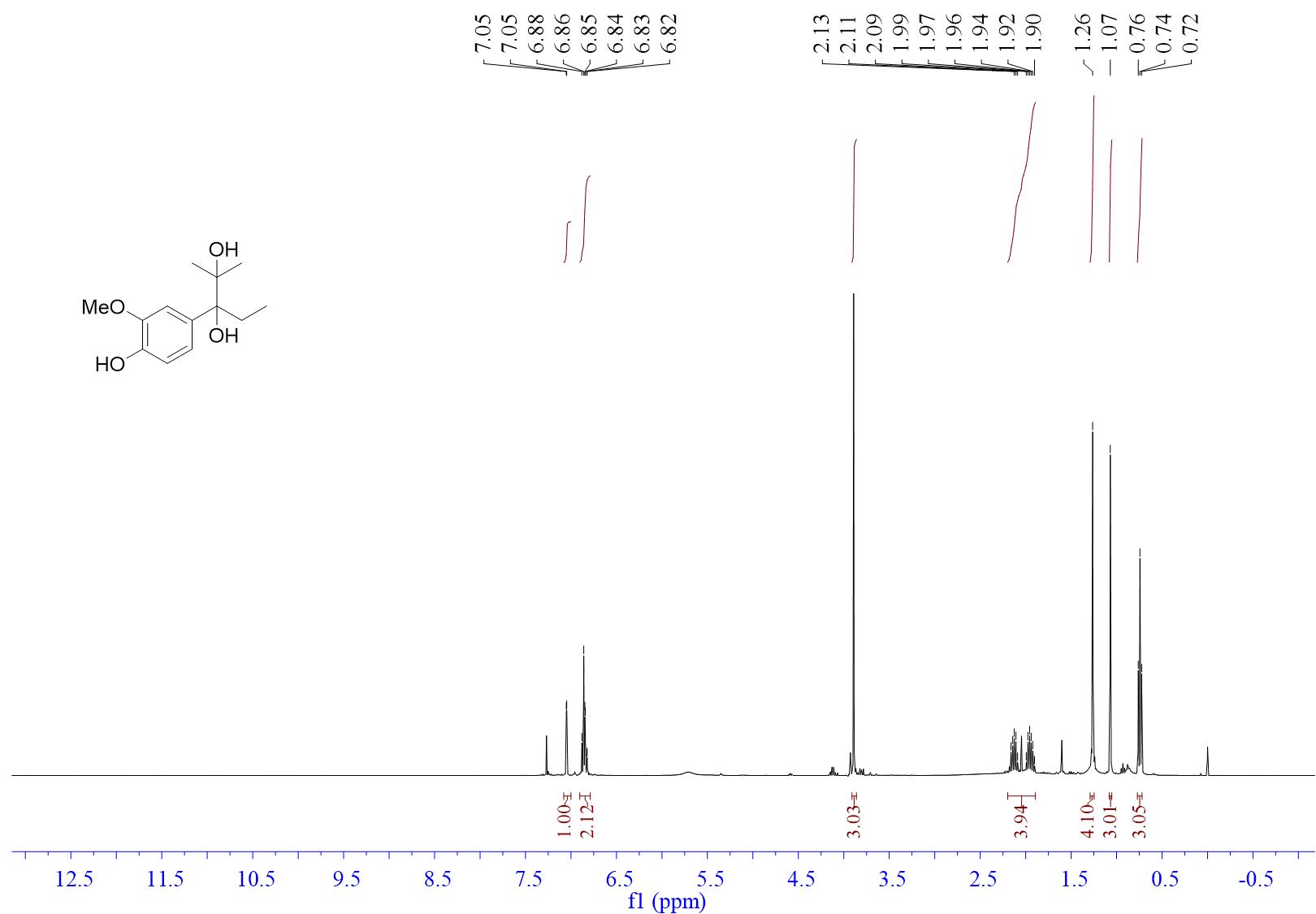


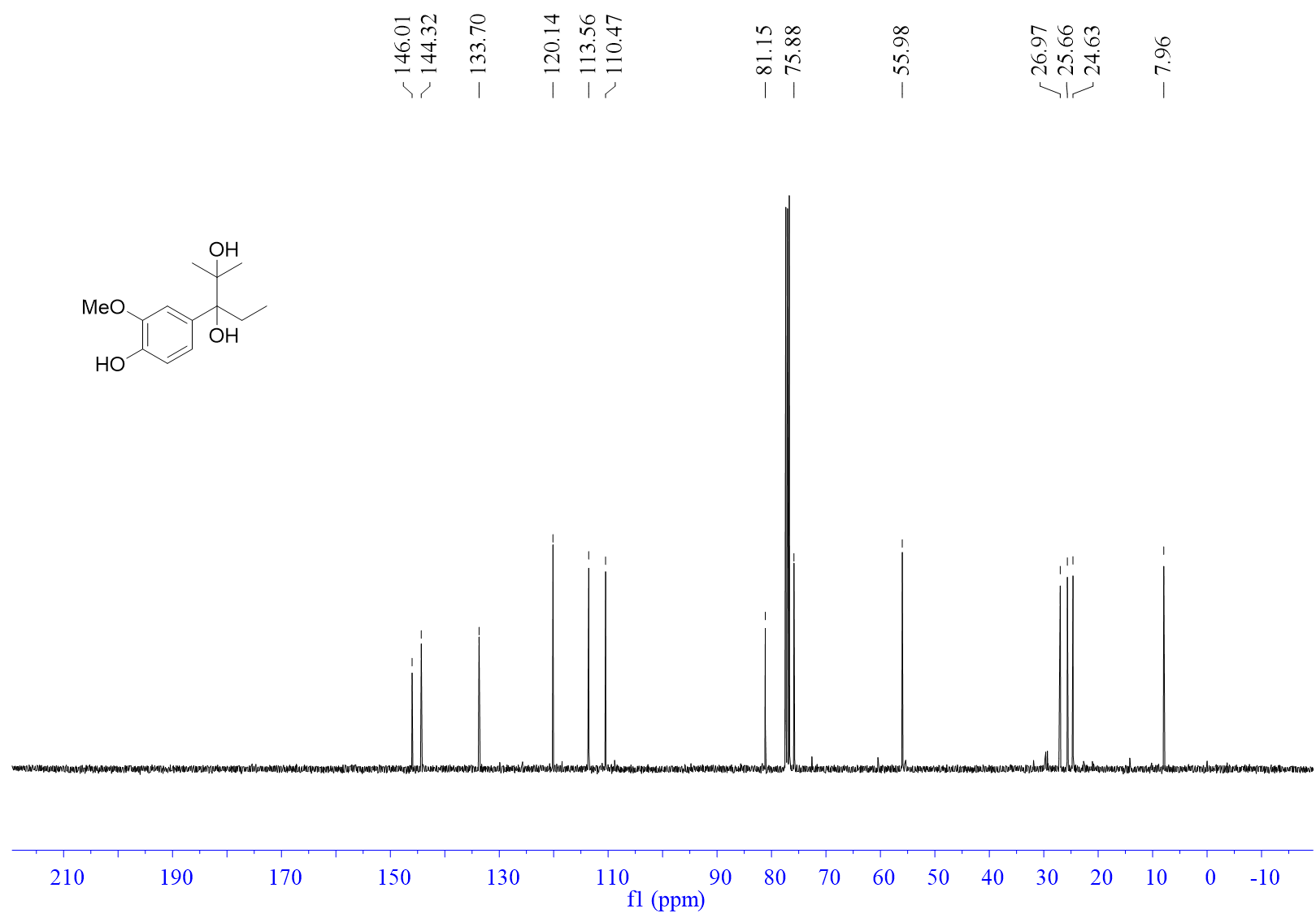


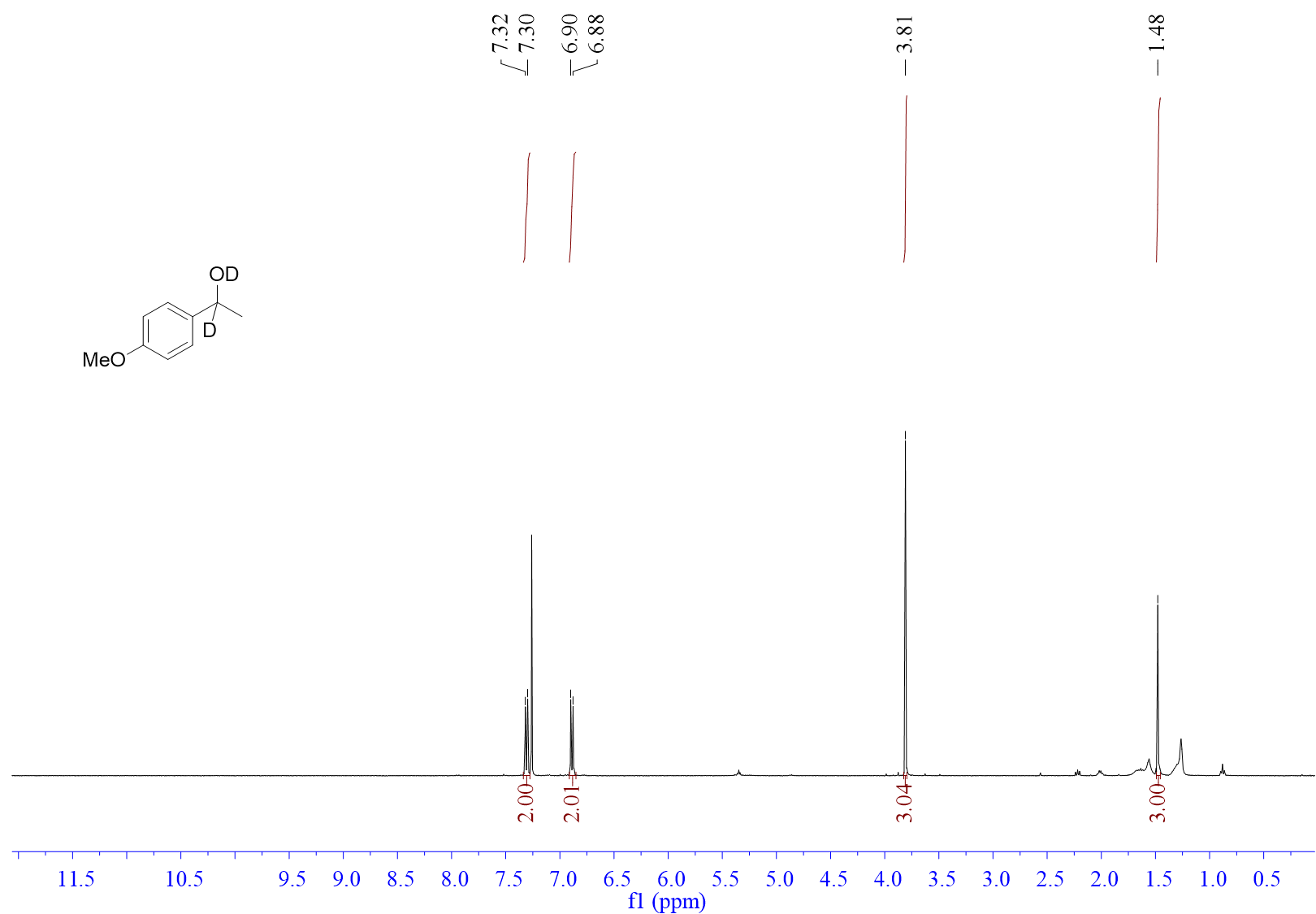


**References**

[1] H. Li, X. Sun, T. Li, Z. Zhao, H. Wang, X. Yang, C. Zhang, F. Wang, *Nat. Commun.* **2024**, *15*, 10176.

[2] W. Lan, M. T. Amiri, C. M. Hunston, J. S. Luterbacher, *Angew. Chem. Int. Ed.* **2018**, *57*, 1356.

[3] R. N. Carafa, J. J. S. Kosalka, B. V. Fernandes, U. Desai, D. A. Foucher, G. G. Sacripante, *Molecules* **2025**, *30*, 2604.
